# Framework for responsive financing of district hospitals of India

**DOI:** 10.3389/fpubh.2024.1398227

**Published:** 2024-10-16

**Authors:** Shankar Prinja, Gaurav Jyani, Aarti Goyal, Sameer Sharma, Tarandeep Kaur, Thiagarajan Sundararaman

**Affiliations:** ^1^Postgraduate Institute of Medical Education and Research, Chandigarh, India; ^2^Jawaharlal Institute of Postgraduate Medical Education and Research, Puducherry, India

**Keywords:** health financing, healthcare cost, blended payment, strategic purchasing, district hospitals, health budget, budget impact, provider payment

## Abstract

**Introduction:**

The current financing of public-sector district hospitals in India relies on historical budget allocations rather than actual utilization or healthcare needs. We utilized empirical data on healthcare delivery costs to develop the financing framework for these hospitals using a blended payment approach.

**Methods:**

The primary data on cost of delivering services in 27 district hospitals across nine states of India was analysed along with indicators influencing the demand and supply of health services. Payment for outpatient, inpatient, and indirect services was assessed using the risk adjusted global budget, case-based bundled payment, and per-bed-global budget, respectively. Risk adjustment weights were computed by regressing the cost of outpatient care with demand and supply side factors which are likely to influence the utilization or the prices. Budget impact analysis was conducted to assess the fiscal implications of this payment approach, accounting for current care standards and two scenarios: upgrading district hospitals to Indian Public Health Standards (IPHS) or medical colleges.

**Results:**

The average annual budget for a district hospital in India is estimated at ₹326 million (US$3.35 million), ranging from ₹66 million to ₹2.57 billion (US$0.8–31.13 million). Inpatient care comprises the largest portion (78%) of the budget. Upgrading to IPHS-compliant secondary hospitals or medical colleges would increase average budgets by 131 and 91.5%, respectively.

**Conclusion:**

Implementing a blended payment approach would align funding with healthcare needs, enhance provider performance, and support ongoing financing reforms aimed at strategic purchasing and universal health coverage.

## Introduction

Globally, district or regional level hospitals serve as the pivotal institutions for dispensing secondary healthcare services (1–3). In the absence of a robust gate-keeping mechanism, district hospitals additionally address substantial proportion of the requirements pertaining to primary level healthcare services (4, 5). Likewise, apart from supporting the primary level healthcare functions, district hospitals in India are meant to serve the backbone of the secondary level public healthcare delivery by providing comprehensive specialist care in a district (6, 7). As a result, district hospitals are not only the major consumers of health budget, but they also employ a sizable chunk of health workforce (8). Nevertheless, the current system of healthcare financing for the district hospitals is primarily passive, and they are financed through classic supply-side financing route, wherein the funds are transferred on the basis of historical or predetermined budgets, without much active consideration of efficiency or health needs of the population being served (8–11).

The existing passive provider payment mechanism should be reformed by incorporating learnings from strategic purchasing, for enhancing the health system goals of equity and efficiency (12). The global experience with different methods of provider payment [fee-for-service (FFS), capitation, case-based payments, global budget, per-diem payments, pay for performance (P4P), etc.] has shown their distinct benefits and limitations (13). For instance, global budget and capitation are expected to have a strong effect on cost containment, but may negatively impact productivity and quality. In contrast, case-based payments including diagnosis related group (DRG) based payments, FFS, and *per diem* payment methods encourage providers to deliver more and better services, but have no incentives for restraining costs, unless these forms of provider payment are applied within the framework of hard budgets or in the case of a tight regulatory environment.

In low- and middle- income countries (LMICs), the choice of provider payment mechanisms varies significantly depending on the structure of the health system, the level of economic development, and the specific healthcare challenges faced by each country (14, 15). Many LMICs in Sub-Saharan Africa have traditionally relied on fee-for-service payment mechanisms, where providers are reimbursed based on the quantity and type of services delivered (16). Countries, such as Thailand and the Philippines, have implemented capitation payment mechanisms, where providers receive a fixed amount per patient, regardless of the number of services provided (17). Countries like China have experimented with global budgeting, where healthcare providers receive a fixed budget to cover a range of services for a specific period (18). Rwanda and Zimbabwe have adopted performance-based financing models, where payments are tied to the achievement of specific health outcomes or quality benchmarks (19, 20). Some LMICs are beginning to explore blended payment models that combine elements of different payment mechanisms.

Global experience therefore suggests that the successful health financing models have attempted to use multiple payment methods (blended payment model), which are characterized by a layering of individual provider payment method (e.g., FFS, capitation, DRGs) and/or ‘add-on’ incentives that are applied to individual or multiple providers. The rationale behind using a blended payment model is that a combination of alternative methods can compensate for the weakness of a single provider payment scheme. For example, in China, the outpatient services are being paid by capitation, global budget and FFS, inpatient services are being paid by DRG, global budget and FFS, and public health services are being paid by FFS and global budget (21). In England, the outpatient services are being paid by fixed global budget which constitutes salary and capitation payments determined according to a refined weighted capitation rubric that takes into account the sex and age of the patients, the number of new patients, the morbidity profile of the population, rurality and the market forces factor, whereas the inpatient services are being paid by DRG and pay for performance (22). Thailand uses a combination of adjusted capitation and FFS for outpatient services; DRG, global budget, and FFS for inpatient services; and capitation and global budget for public health services (23). Such blended provider payment systems are being followed by many countries and they have consequently reported improvement in health system performance by improving equity, accessibility, affordability, and efficiency in delivering healthcare services (24).

Nevertheless, there is a lack of empirical evidence on effective financing mechanisms tailored to district hospitals in India which accounts for diverse healthcare needs of the local population and considers context-specific solutions (25, 26). Furthermore, blended payment models have not been explored so far as a means to enhance the efficiency and responsiveness of healthcare financing in Indian settings (27). As a result, there is an urgent need for a strategic framework that aligns financial payouts to the district hospitals with healthcare outcomes at the district hospital level, addressing the unique challenges posed by varying population health needs. As different district hospitals of India face varying healthcare demands and challenges, the blended payment framework will provide flexibility in adapting to local needs and consequently allow them to respond to changing healthcare priorities and emerging health threats effectively (28). Therefore, we undertook this study to develop a blended payment model for financing of district hospitals of India, and empirically estimate the level of payment for the district hospitals in India using this framework. We estimated the annual payments which 845 district hospitals of India will receive based on the current healthcare needs, infrastructure, as well as utilization patterns. Finally, we compute the financial pay-out in two additional scenarios, i.e., if the district hospitals are upgraded as per the recommendations of the Indian Public Health Standards (IPHS), and if the district hospitals are upgraded to medical colleges providing tertiary care services, as both these scenarios entail additional budgetary allocation to these hospitals (29). By strategically capitalizing on the proposed blended payment model, policymakers can address the challenges faced in resource-constrained settings, by effectively allocating resources to areas of greatest need, and promoting the optimal utilization of available resources.

## Methods

### Conceptual framework of the analysis

**Figure 1:**
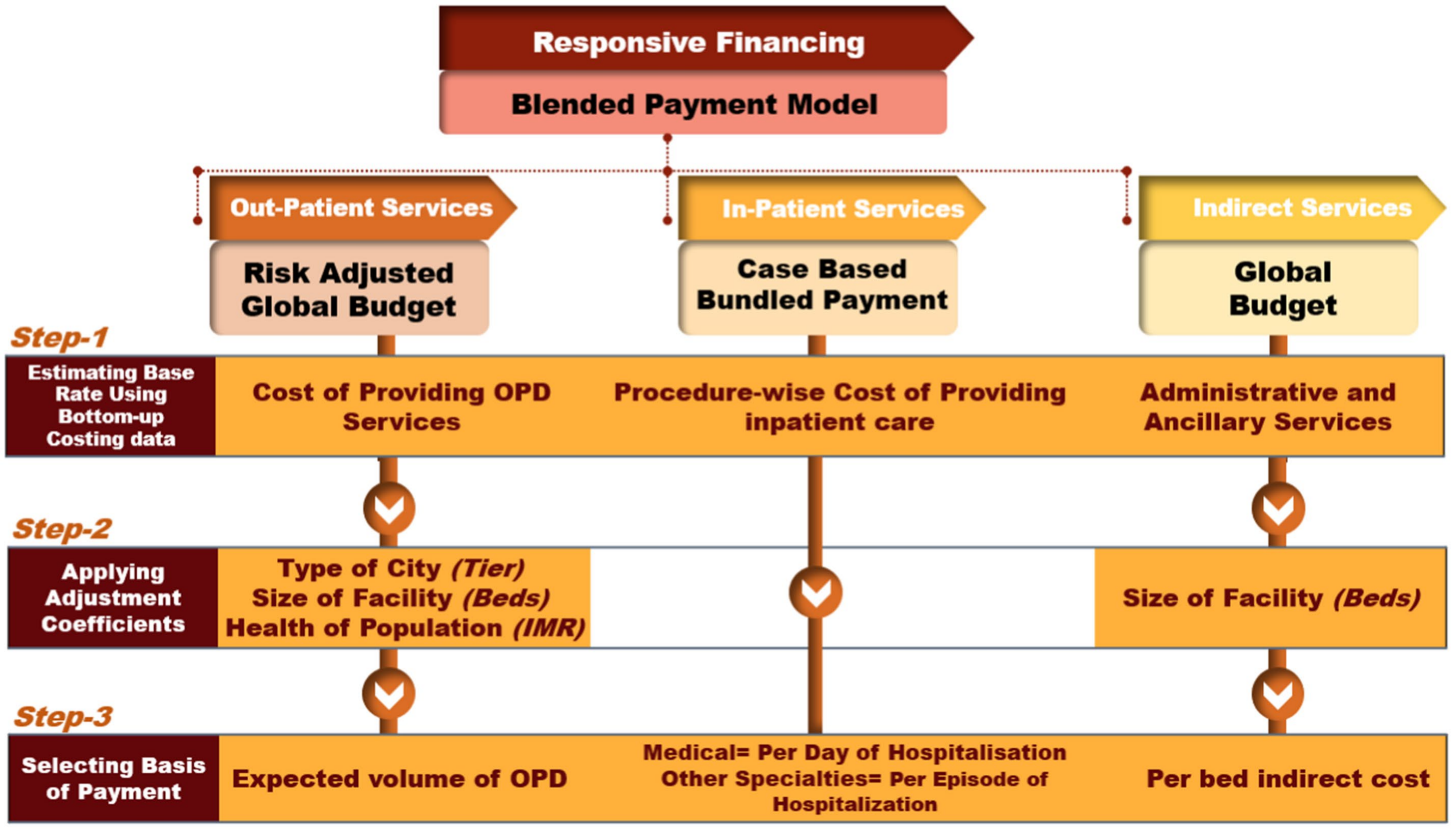
Conceptual framework for determining responsive healthcare financing of district hospitals.

We propose a conceptual framework, consisting of a mix of the provider payment methods to determine the requirements for responsive financing of the district hospitals. The structure of the proposed model has been determined based on the existing global evidence on the payment of healthcare services, availability of the required data in the Indian settings, findings of our analysis, and consistency with the existing health financing structure of the country. In the proposed blended payment model, the outpatient services are paid using a risk adjusted global budget, the inpatient services are paid using the case based bundled payments, and the indirect services are paid using the global budget which is estimated based on hospital size (Figure 1). The software package STATA version 13 (StataCorp, College Station, TX, USA) was used for the analyses.

### Estimation of budget for out-patient care

For determining the risk-adjusted global budget for outpatient care, the association between the cost of providing outpatient services and different factors influencing the healthcare needs and the resource requirement was assessed using multiple linear regression. For this, first we identified potential demand and supply side factors known to impact healthcare service demand and associated costs and select those for which valid and regularly generated data are available at the district level. Then we obtained cost of providing out-patient services in district hospital from cost of health services in India (CHSI) study data. The CHSI study provided estimates on cost of delivering health services from 27 district hospitals across nine Indian states (30, 31). The CHSI study was commissioned by the Department of Health Research, Ministry of Health and Family Welfare, Government of India, in 2018 to generate credible evidence on the cost of healthcare services using a standardized costing methodology (32). The period of data collection was 2018–20. The study employed a mixed methodology consisting of both bottom-up and top-down costing approaches. The selection of 27 district hospitals in CHSI study was guided by the consideration to the heterogeneity in the districts of India based on the geography, human development index (HDI), gross state domestic product (GSDP), and health workforce density. Therefore, the district hospitals within a given state were selected using stratified sampling. All the district hospitals of a state are stratified in three tertiles based on a composite index drawn from socioeconomic, demographic and healthcare utilization indicators, and one district hospital was randomly selected from each of the three tertiles (30). In CHSI, the estimation of costs for delivering outpatient, inpatient, and indirect services involved considering multiple categories of expenditures to ensure a thorough analysis. For all outpatient, inpatient, and indirect services, the cost estimation encompassed expenditures related to human resources (including salaries for medical staff such as doctors, nurses, and support personnel), the costs of medical equipment, the cost of drugs and consumables (medical and surgical supplies). Additionally, overheads such as utility costs for water and electricity, along with capital expenditures on land and buildings were also integral parts of the cost assessment.

Thereafter, to explore the relationship between the identified factors and outpatient care cost, we employ a multiple linear regression model using the ordinary least square (OLS) method. A list of potential demand and supply side factors which are reported to influence the demand for healthcare services, or the cost of services was prepared (29, 33–35). Thereafter, those factors for which valid data are generated regularly at the district level, and which are not amenable to reporting errors were chosen. For example, district-specific illness rate is likely to be an important determinant of outpatient care cost. However, a review of nationally representative surveys had suggested that the data on the illness rate is not available at the district level, and at a regular frequency (35). Furthermore, due to the absence of electronic health records, the computation of illness rate is based on the data on self-reported illness collected during household surveys, which does not imply a definitive clinical diagnosis and can be confounded by positional objectivity (36, 37). On the other hand, the district-wise data of IMR is available in India at a regular frequency (34). IMR is also considered a reliable indicator of population health (38–40). In view of this, we used district specific IMR as an indicator of population health need.

We also used the data on the size of the district hospital (number of beds), location of the district hospital (tier of the city determined by its total population) and extent of utilisation of public health services in a particular district to determine the pay-out to a district hospital (29, 35, 41). The association of the outpatient care cost was assessed with the location of district hospital (type of city), district population size, IMR, proportional utilization in public hospital, proportion of population under 5 years and above 60 years of age (%), and bed strength of district hospital [the total number of beds available in the inpatient wards of a hospital (41–43)]. To generate a sample of 100 health facilities, multivariate normal distribution (MVND) (44) was applied for simulating data for tier 1, tier 2 and tier 3, separately based on different characteristics of 27 district of CHSI dataset (30, 31). Consequently, we obtained a dataset comprising a total of 100 observations that were incorporated into our model. This dataset is composed of 7 observations pertaining to tier-1 cities, 29 observations associated with tier-2 cities, and 64 observations corresponding to tier-3 cities. In order to explore the relationship between these factors and outpatient cost, we applied multiple linear regression model using ordinary least square method. The model selection was based on characteristics of dependent variable, as well as the assumptions of model, i.e., normality of regress and error term, presence of homoscedasticity, and no multicollinearity. Normality of regress and error term for the model was checked using “Kolmogorov Smirnov Test” with insignificant *p*-values (0.854), and error term (0.701). The presence of homoscedasticity was checked using “Breusch-Pagan Test” with insignificant p-value (0.394), which failed to reject the null hypothesis of homoscedasticity. Thus, the assumptions of normality of regress and, the error term and presence of homoscedasticity were fulfilled for the model. There was no multicollinearity, with variance inflation value between 1 and 5. R^2^ and adjusted R^2^ scores were also used to evaluate the model’s performance. The detailed statistical analysis has been provided in Supplementary material S1.

### Estimation of budget for in-patient care and indirect services

For estimation of budgetary requirement for providing inpatient care, the case based bundled payments were determined using the cost of health benefit packages (HBPs) as defined under India’s national insurance program- PM-JAY. We used the annual (2019) data on health insurance claims for various health benefit packages at the district hospital level under India’s largest publicly funded national insurance program (*Ayushman Bharat Pradhan Mantri Jan Arogya Yojana*: AB PM-JAY) to obtain a distribution of the outpatient and inpatient services by the type of morbidity (45). The detailed methodology used in determining the cost of HBPs has been reported separately (30, 31). Thirdly, the global budget for providing the indirect services was determined using the primary data on cost of providing these services in the 27 district hospitals. The indirect services included costs for administration, patient registration, governance, biomedical waste management, laundry, and dietetics. These services are vital for supporting the hospital’s infrastructure and ensuring a conducive environment for healthcare delivery. As the budget requirement of carrying out the indirect services corresponds with the size of the hospital, bed strength was used as the indicator to determine the indirect services budget to a district hospital.

All costs are reported in Indian National Rupee (₹) and US$ using the average conversion of US$ 1 = ₹ 82.54 in January 2024 (46).

### Budget impact assessment

#### District hospitals operating at existing standards

To evaluate the financial consequences of implementing this responsive resource allocation approach for financing India’s district hospitals, we calculated the total annual payout for all the country’s district hospitals. The computation of payout for outpatient care for a particular district hospital was derived from the coefficients produced in our regression analysis and district-specific parameters. The payout for inpatient services was determined using case-based bundled payments, which were derived from the CHSI study. The estimation of payout for indirect service was done using bed-size of the hospital and per-bed annual cost of providing indirect services, estimated using CHSI dataset.

The budgetary requirement for providing inpatient care was assessed at the specialty level, and computed as a product of the cost of delivering the services under HBPs as defined under *PM-JAY*, and the number of cases corresponding to each of the HBP in the preceding year (31). However, the district hospitals also provide inpatient care for procedures which may not be part of the *PM-JAY* health benefit package. For such diseases/ procedures which are not covered under *PM-JAY*, the cost of providing inpatient care was assessed using the specialty-specific weighted average cost and volume of non-PM*-JAY* admissions (details in Supplementary material S1).

The estimation of HBP cost was done using a health system’s/payer’s perspective, wherein the economic cost of providing the health services was calculated considering full recurrent cost and 20% of the capital cost. To estimate the total number of admissions that are likely to take place annually in a given specialty of a district hospital, we first obtained the number of hospital beds available in each of the district hospital from National Health Profile of India (41). Thereafter, using the CHSI data on speciality-wise distribution of beds and the average length of stay within each specialty, we derived the expected number of annual hospitalizations in each specialty for all the district hospitals, assuming the bed occupancy rate as observed in the CHSI data. We divided the number of admissions in each specialty between *PM-JAY* and non-*PM-JAY* hospitalisations as observed in the CHSI study sample and applied it to all the district hospitals.

The global budget for providing the indirect services was determined using the primary data on cost of proving these services in the 27 district hospitals. As the budget requirement of carrying out the indirect services corresponds with the size of the hospital, bed strength was used as the indicator of the size of the hospital while determining the total budget of providing the indirect services in a district hospital.

In addition to the base case, we conducted two distinct sensitivity analyses as part of the budget impact assessment. In the first sensitivity analysis, we evaluated the budgetary impact under a scenario where district hospitals are upgraded to meet the Indian Public Health Standards (IPHS). The second sensitivity analysis assessed the financial implications of upgrading district hospitals to the status of medical colleges. The details of these sensitivity analyses are provided below.

#### District hospitals upgraded to IPHS standards

In the scenario where the district hospitals are upgraded to the IPHS recommendations, there will be an augmentation of several resources which include human resources, infrastructure (capital), drugs, and consumables. Thereby, we also estimated the financial pay-out in a scenario where the district hospitals are upgraded as per the recommendations of the IPHS (29). While calculating the annual pay-out to a district hospital, we have factored in all the recurrent cost, and 20% of the capital cost.

Using the data of the current staffing pattern of 27 district hospitals, we first assessed the extent of shortfall of healthcare personnel at district hospitals. The shortfall of staff was assessed at the specialty level and for three categories of healthcare personnel, i.e., doctors, paramedical staff, and support staff. Thereafter, using the existing salary structure of the healthcare personnel, the additional pay-out required to upgrade the district hospitals to the IPHS staffing requirements was calculated.

In a district hospital, drugs and consumables are primarily utilized in providing inpatient care. The increased utilization of drugs and consumables due to the upgradation of the health facility is automatically factored in the proposed blended payment model, as the payment for inpatient services has been determined on the basis of volume of services delivered in the impatient departments. Moreover, as IPHS norms only specifies the type of drugs, consumables and equipment needs to be available at a health facility, and not their quantity, the present approach offered the most plausible method to estimate the pay-out in case of upgrading the facility as per the IPHS norms. As capital upgradation is a gradual process, rather than proposing the entire shortfall cost for capital upgradation forthwith, we proposed it at the annual rate of one-fifth of the existing total capital cost, as the utilisation of outlay for capital expenditure takes time.

#### District hospitals upgraded to medical colleges

In order to address the growing healthcare demands and the shortage of health workforce in the country, the government of India is establishing additional medical colleges which are being attached with the existing district hospitals (47). Consequently, certain district hospitals are being upgraded for affiliation with these medical colleges. The process of upgrading a district hospital to a medical college involves the allocation of additional resources, including personnel, capital, infrastructure, medications, and consumables. In light of this, we have conducted a scenario analysis to estimate the financial implications associated with upgrading district hospitals to medical colleges. The framework presented in this scenario analysis can be utilized to determine the financial payout for those district hospitals that have been identified for the upgradation to medical colleges. For this part of analysis, we reanalyzed the data of CHSI study pertaining to cost of providing healthcare services in 11 public sector tertiary care health facilities (31, 32).

We further note that in certain states, the upgradation to medical colleges overlaps with efforts to bring down super-specialty tertiary care to the district level. Thus cardiology, neurology, nephrology services are provided in district hospitals, even though they are not formally designated as medical colleges. For this study we consider such upgradations also under the medical college upgradation category.

In this scenario, we assessed the payment required for outpatient services using a risk-adjusted global budget model. This involved regressing the cost of care in these tertiary hospitals against potential demand and supply-side factors, consistent with the base-case analysis. Supplementary material S1 provides the additional details of the statistical model used for estimation of responsive resource allocation for outpatient care services for a district hospital which is being upgraded to medical college. The payment for inpatient services was determined using a case-based bundled payment approach, with costs derived from health benefit packages provided in public sector tertiary hospitals. Furthermore, the determination of the global budget for indirect services in the hospitals identified for upgrade to medical colleges was performed using primary data on the costs associated with these services in the 11 tertiary hospitals. The bed strength of each hospital was used as a proxy for hospital size, informing the calculation of the financial outlay for indirect services.

### Comparison with existing annual pay-out to district hospitals

The pay-out to different district hospitals estimated as per the proposed financing norms was compared to the existing government health expenditure at district hospitals in India. Two sets of comparison were made to assess the validity of the pay-out using the proposed financing norms. Firstly, the mean proposed annual pay-out to a district hospital in India was compared to the mean government health expenditure at a district hospital. The estimates of current government expenditure were calculated using the data of National Health Accounts of India (2018–19) on government expenditure on secondary care, and the National Health Profile of India (8, 41). We also compared the proportional allocation of total pay-out to district hospitals for outpatient, inpatient, and indirect services as estimated using the proposed financing framework with the actual distribution of allocation as reported in the National Health Accounts of India (8). The National Health Accounts for year 2018–19 was chosen for the comparison as it corresponds with the year of cost data collection in CHSI study, which is used for the proposed financing reforms (30). Secondly, we also obtained the health accounts data on fund flows to secondary hospitals in one of the South Indian state, i.e., Tamil Nadu, and then compared the actual and proposed pay-out to each of the district hospital in the state of Tamil Nadu in year 2017–18 (48).

The state of Uttarakhand in India has developed a model of strategic purchasing of healthcare services by contracting out the district hospitals to private providers in some of the districts (49). The rates of payment under this model were derived through a process of competitive bidding and tendering. Under this model, the health department of Uttarakhand handed over the hospital building and all its equipment to the contracted private partner. Besides this, the government was obligated to provide the drugs and consumables by procuring them in the centralized manner. The entire government funded manpower which was previously stationed at the hospital was removed, and the private service provider was asked to bring in its own human resources and run the hospital. We obtained the annual pay-out made by the government to the private provider for contracting out of district hospital in Baurari district of Tehri Cluster of Uttarakhand in the year 2020–21 and compared it with the pay-out estimated using the proposed blended payment model for this district hospital.

## Results

### Allocation of budget for out-patient care

We found a statistically significant association of cost of providing outpatient care (dependent variable) with type of city where hospital is located, size of the hospital, and IMR (independent variables) (Table 1). The results demonstrated that tier 2 and tier 3 cities have lower cost with significant magnitude of difference (*p* value = 0.002, *p* value<0.001, respectively). An increase in bed strength (*p* < 0.001) and infant mortality rate (*p* < 0.001) increased the cost for outpatient care. Based on this multiple linear regression model, annual financial payout for providing outpatient care in different district hospitals of India was assessed using district specific indicators (Supplementary material S2).

**Table 1:** Model for estimation of responsive resource allocation for outpatient care services at a district hospital.

		Unstandardized coefficient	SE	95% CIs
Intercept		75400000*	32,900,000	(10,000,000, 141,000,000)
Type of city (Ref. Tier 1)	Tier 2	−34300000**	10,700,000	(−55,500,000, −13,200,000)
	Tier 3	−51400000**	11,000,000	(−73,300,000, −29,500,000)
Population of the district		60,858	126,738	(−190,854, 312,570)
Bed strength of district hospital		89121**	16,692	(55,970, 122,272)
Infant mortality rate		565977**	171,385	(225,591, 906,363)
Population under 5 and above 60 years of age (%)		−1,673,450	1,227,806	(−4,111,979, 765,079)
Utilization of Public health services (%)		−20,079	166,842	(−351,443, 311,284)
$R^{2}$		0.5603		
Adjusted $R^{2}$		0.5268		

All coefficient values in ₹; SE = Standard error; CI = Confidence interval; * *p* value is significant when ≤ 0.1; ** *p* value is significant when ≤ 0.05.

### Allocation of budget for in-patient care and indirect services

The cost for all the procedures specified in the *PM-JAY* has been provided in Supplementary material S3. The specialty-wise weighted cost for non- *PM-JAY* cases has been provided in Table 2. The average annual cost per bed for indirect services was estimated as ₹ 1,63,969 (US$ 1986).

**Table 2:** Specialty-wise weighted costs for non- *PM-JAY* cases.

Department	Cost per hospitalization* ₹ (US$)
Paediatrics	10,415 (126.15)
General Medicine	10,481 (126.95)
Obstetrics and gynaecology	13,286 (160.93)
Ear Nose and Throat	14,104 (170.84)
General Surgery	13,011 (157.6)
Ophthalmology	9,867 (119.52)
Orthopedics	13,925 (168.67)
Overall	12,156 (147.24)

^*^Inclusive of full recurrent cost and 20% of fixed cost.

### Budget impact of using responsive resource allocation at the national level

#### District hospitals operating at existing standards

**Figure 2:**
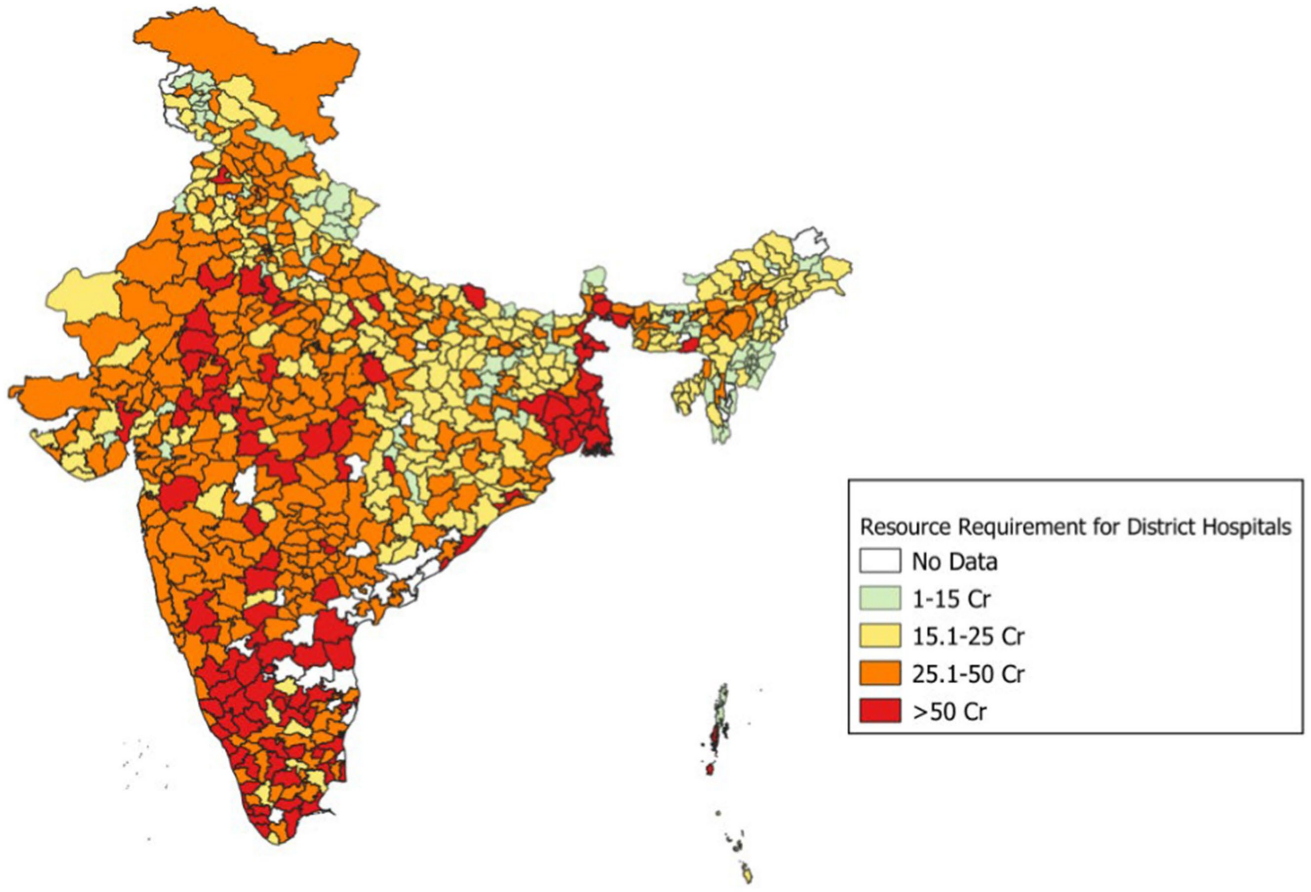
Estimated budget requirement using blended payment reforms in different district hospitals of India.

**Figure 3:**
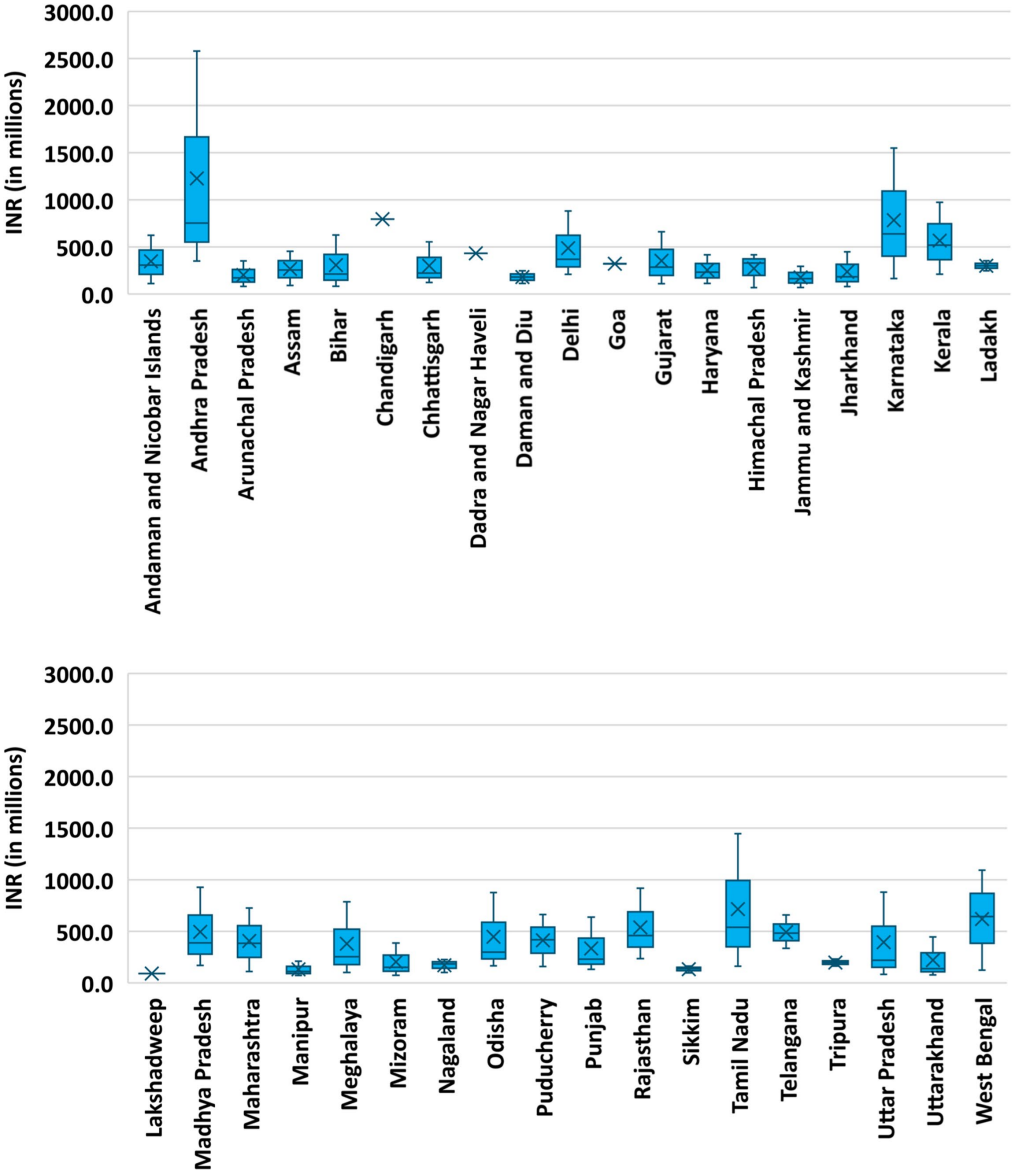
Estimated intra-state variation in budget requirement for district hospitals using blended payment reforms.

The mean annual pay-out for a district hospital in India using the proposed blended payment method is estimated to be ₹ 326 million (US$ 3.35 million), with a range of ₹ 66 million to ₹ 2.57 billion (US$ 0.8–31.13 million) (39 times). The largest share of the district hospital budget is attributed to inpatient care, with the mean annual budget of ₹ 221 million (US$ 2.68 million) with a range of ₹ 178 million to 2.01 billion (US$ 2.16–24.35 million) (11 times). The mean cost of providing outpatient and indirect services is estimated to be ₹ 68 (33–224) million [US$ 0.82 (0.4–2.71) (7 times) million], and ₹ 36 (2.95–334) million [US$ 0.44 (0.04–4.05) million] (101 times), respectively (Figures 2, 3). Figure 2 shows the estimated budget requirement for providing healthcare services in different district hospitals of India. Figure 3 describes the intra-state variation in the overall estimated pay-out to the different district hospitals within a state/ union territory of India. Our analysis shows that the average annual pay-out to a district hospital varies from ₹ 66 million (US$ 0.8 million) in Himachal Pradesh to ₹ 2.57 billion (US$ 31.13 million) in Andhra Pradesh. Similarly, the range of district hospital pay-out may vary by as low as 1:1.02 in Goa to 1:10.65 in Uttar Pradesh.

#### District hospitals upgraded to IPHS standards

If the district hospitals are staffed as per the recommendations of IPHS, the mean annual pay-out for a district hospital in India using the proposed blended payment method is estimated to be ₹ 751 million (range = 218 million to 5.42 billion) [US$ 9.1 (2.64–65.65) million]. In this scenario also, the largest share of the district hospital budget is attributed to inpatient care, with the mean annual budget of ₹ 508 million (range = 41 million to 4.63 billion) [US$ 6.15 (0.5–56.08) million]. The mean cost of providing outpatient and indirect services is estimated to be ₹ 206 million (range = 156 million to 452 million) [US$ 2.5 (1.89–5.47) million], and 36.7 million (range = 2.95 million to 335 million) [US$ 0.44 (0.036–4.06) million], respectively.

#### District hospitals upgraded to medical colleges

When the district hospitals are upgraded to medical colleges, the mean annual pay-out increases by 91.5%. The financial pay-out for providing outpatient care for a hospital which is being upgraded to a medical college is estimated based on the multiple linear regression model using district specific indicators (Supplementary material S1).

The results of scenario analysis suggest that when the district hospitals are upgraded to medical colleges, the mean annual pay-out for inpatient services increases by 123%. Likewise, per bed annual global budget for providing indirect services also increases from ₹ 1,63,969 (US$ 1986) to ₹ 2,94,441 (US$ 3,566) in this scenario. The total annual financial pay-out for individual district hospitals in the scenario where they would be upgraded to medical college has been calculated using district specific indicators and has been provided in Supplementary material S4.

### Comparison with existing pay-out

As per the simulated blended payment method, the mean annual pay-out for a district hospital in India was estimated to be ₹ 326 million (US$ 3.95 million). Using the current financing framework, mean annual pay-out to a district hospital in India is 321.8 million (US$ 3.9 million). Likewise, the National Health Accounts estimates suggest that in a district hospital, the share of expenditure on outpatient, inpatient, and indirect services in the total pay-out is 19, 58, and 23%, respectively. If the costs of care in district hospitals are estimated as per the simulated blended payment method, the share of expenditure on outpatient, inpatient, and indirect services in the total pay-out will be 21, 63, and 16%, respectively.

**Figure 4:**
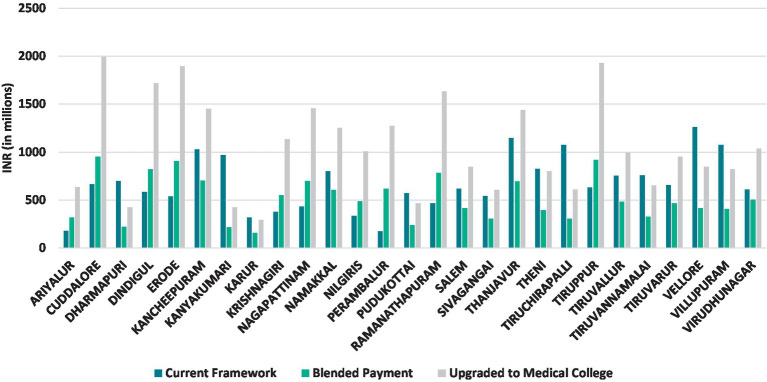
Comparison of the current annual pay-out and proposed annual pay-out to district hospitals in Tamil Nadu.

Furthermore, a comparison of the current and estimated annual pay-out to each of the district hospital in the state of Tamil Nadu showed that the estimated pay-out using blended payment framework varied from −77 to 83% for individual district hospitals (with the exception of Perambalur, where the variation was 2.6 times higher using blended payment framework). The district-wise comparison is presented in Figure 4. It implies that the state is under-funding most hospitals and since the simulation is based on costs of care, the under-funding must be leading to loss of quality, which would be a matter of concern. Some district hospitals whose current payments have a higher pay-out could be checked for potential increase in efficiency, though it is most likely that the composition and volume of services provided is more in line with medical college hospitals. Thus, such comparisons using blended payment reform will lead to significant improvements in the budgets provided to individual district hospitals, as it becomes more responsive to the health care needs of the population being served and the performance of the healthcare providers.

Another interesting comparison is the annual pay-out made by the government to the private providers for contracting out of district hospital in Baurari district of Uttarakhand is ₹ 140.03 million (US$ 1.7 million). By our approach, the mean annual pay-out to the district hospital of Baurari, for the set of resources which are proposed to be paid as per contract, was estimated to be ₹ 127.8 (US$ 1.55 million) million. This implies that our simulated budget is not very different from a scenario where the payment has been derived from explicit negotiation with a limited set of providers, and rather there is scope for better price negotiations.

## Discussion

Healthcare systems globally are exploring innovative approaches to encourage the provision of high-value care (50). Creating a harmonious blend of payment methods, strategically devising them, and establishing appropriate implementation arrangements are pivotal to maximize the advantages that health systems can reap by setting appropriate incentives through a provider payment system. In this light, our paper describes the development of a framework for allocation of payments for the district hospitals in India, which will be responsive to the healthcare needs and likely to incentivize and support service delivery and efficiency.

The blended payment framework utilized in our paper estimates mean annual government expenditure of ₹ 326 million (US$ 3.95 million) in a district hospital, which ranges from ₹ 66 million to ₹ 2.57 billion (US$ 0.8–31.13 million). The share of outpatient, inpatient, and indirect services in the total pay-out to a district hospital would be 21, 63, and 16%, respectively. These findings are consistent with the estimates of National Health Accounts of India, wherein the mean annual budget allocation to a district hospital has been reported as ₹ 322 million (US$ 3.9 million) (8). This comparison suggests that the implementation of the proposed framework would not change the overall pay-out made to all district hospitals. Instead, it aims to redistribute the existing funds among different district hospitals more efficiently, ensuring that resources are allocated based on the specific health needs of population and service delivery requirements of each hospital. The analysis showed that the proposed model redistributes existing funds more effectively, leading to a more rational and equitable allocation of resources across varying healthcare contexts, ensuring that each hospital’s operational needs are met without increasing the total financial burden on public finances.

Our analysis found that an increase in bed strength and IMR increased the cost for outpatient care. The IMR is used as a proxy indicator of healthcare demand and the overall burden on the healthcare system in our analysis. Higher IMR often reflects underlying issues such as poor maternal and child health services and inadequate access to quality healthcare. These factors contribute to increased healthcare needs, particularly in the outpatient setting, where early and frequent medical intervention is critical for addressing preventable causes of infant mortality. As infant mortality rate rises, district hospitals may experience a surge in outpatient visits, driven by an increased demand for pediatric care, maternal health services, and other preventive and curative interventions aimed at reducing infant deaths. This escalation in demand inevitably leads to higher outpatient costs as hospitals are required to allocate more resources—such as human resources and infrastructural capacity to meet the needs of the population. Similarly, the bed-strength in hospitals is an indicator of the hospital’s size and its ability to manage patient load. Larger hospitals with more beds have higher operational costs. As bed strength increases, hospitals are often equipped with greater staffing levels, which can drive up the overall costs associated with outpatient care.

Our study is the first attempt to suggest a framework backed by empirical evidence for reforming the financing of district hospitals. By analysing the nationally representative real-world data and utilizing robust scientific methodology, this study offers a novel contribution towards the development of effective strategies for enhancing vertical equity in financing of district hospitals (30, 51, 52). Moreover, as this study provides the norms for financing of outpatient, inpatient, and indirect services, the evidence-based framework proposed in the study can also be used in budgeting of health services. To ensure the robustness of the framework for future applications, we selected explanatory variables that are regularly updated at the district level. By leveraging the Management Information System (MIS) for continuous data updates and adjusting the cost of services with the GDP deflator, the model provides a dynamic and responsive mechanism for estimating future budgets. This approach allows the framework to adapt to evolving healthcare demands and economic conditions, ensuring its long-term applicability.

Nevertheless, the implementation of the suggested financing framework does face specific challenges that need to be acknowledged. These obstacles may include factors such as complex regulatory requirements, the need for substantial organizational and structural changes within the healthcare system, resistance from stakeholders, and budgetary constraints. Overcoming these challenges will require careful planning, effective communication, stakeholder engagement, and phased implementation to ensure successful adoption and integration of the proposed financing framework. A shift towards the proposed blended payment mechanism will require a careful consideration of the current health financing flows to the district hospitals, as these hospitals get their funds through multiple channels of treasury route and non-treasury route (26, 53). In this regard, it would be important to consider retaining certain financing channels where the centralized financing is more efficient, for example, centralized procurement of drugs and medical supplies. Furthermore, as the government has started to use a system of case-based bundled prices for paying inpatient care delivered by empaneled providers under government funded insurance schemes, the mechanism proposed in the study for paying inpatient care is aligned with existing financing reforms under these demand-side financing systems.

### Policy implications

The findings of this study have significant implications for healthcare policy in the context of financing district hospitals in India. The current system of healthcare financing for district hospitals is predominantly passive, with funds allocated through classic supply-side financing mechanisms. These mechanisms rely on historical or predetermined budgets, often without active consideration of the efficiency or the specific health needs of the population being served. This approach limits the ability of district hospitals to respond effectively to varying healthcare demands and emerging health challenges across different regions. The present study underscores the need for a context-specific approach to health financing. The proposed responsive financing framework, which incorporates a blended payment model, represents a strategic shift towards a more dynamic and needs-based approach to funding. By allowing for the integration of risk-adjusted global budgets and case-based bundled payments, the framework offers flexibility in adapting to local needs, enabling district hospitals to better align resources with the specific demands of their patient populations. This flexibility is important for addressing the diverse challenges faced by different district hospitals, thereby ensuring that financial resources are utilized more effectively and efficiently.

Policymakers should consider adopting this blended payment framework as part of broader health financing reforms aimed at strengthening the public healthcare system. The model’s ability to account for factors influencing healthcare demand in determining financial payouts ensures that the financing system is more responsive and adaptable to changing healthcare priorities. This adaptability not only promotes financial sustainability but also incentivizes hospitals to optimize their service delivery, focusing on both efficiency and quality of care.

In order to effectively implement the proposed financing mechanism, it will be imperative to conduct extensive stakeholder consultations. This broader engagement will ensure that the perspectives and insights of various stakeholders, including healthcare providers, administrators, policymakers, and researchers, are considered and incorporated into the implementation process, which will help to address potential concerns, identify practical solutions, and enhance the overall effectiveness and sustainability of the new financing mechanism. Additionally, post-implementation monitoring of the incentives and disincentives within the health system will be essential. Regular and systematic evaluation will enable policymakers and administrators to assess the impact of the new financing mechanism on various aspects of the health system, including quality of care, access to services, cost-effectiveness, and patient outcomes. By closely monitoring these incentives and disincentives, any unintended consequences or areas requiring adjustment can be identified promptly. This monitoring process will facilitate evidence-based decision-making, allowing for ongoing refinement and improvement of the financing mechanism to ensure its alignment with the evolving needs and goals of the health system.

The study findings nudge the shift towards a more value-based care model for financing of healthcare services (54, 55). The ‘value’ offered in the current financing framework is the ‘fairness’ in terms of satisfying the need principle as well as incentivizing performance. This is justified since the payment of outpatient care is linked to the indicators of need, while the payment of inpatient care is based on quantity of services delivered. The financing reforms should be further fine-tuned with better data to incorporate the indicators of quality and overall aspirations of UHC. Given the current experiments of the state governments towards contracting-out the service delivery in district hospitals to the private sector, our study provides empirical estimates to set the norms for provider payment which could become the basis for subsequent contracting of private providers on similar terms to provide much needed supplementary capacity to the public hospitals (56).

### Limitations

There are certain limitations in our analysis. Firstly, the data from 27 district hospitals was simulated using bootstrapping to increase the overall sample utilized for analysis to develop the risk-adjusted global budget for financing the outpatient care. However, bootstrapping is a valid and widely practiced approach to generate robust statistical inferences, even when the original sample size is limited (57). Further, the 27 district hospitals were selected from nine states of India using a scientifically sound criteria and by using indicators of geography, human development index, gross state domestic product, health workforce density, socioeconomic, demographic and healthcare utilization indicators. In spite of that, primary data on cost of delivering health services from an increased number of district hospitals would help to strengthen the proposed financing framework. Moreover, it is worthwhile to mention that the share of cost of outpatient services contributes to around 20% of the total district hospital budget, hence, it is not likely to introduce a significant uncertainty in the overall estimation. Another data limitation of the analysis pertains to the non-availability of disease-wise breakdown of non-*PM-JAY* admissions to the district hospitals. Greater penetration of the digital health records as proposed under the *Ayushman Bharat Digital Mission* (ABDM), as well as standardization of morbidity estimation through adoption of International Classification of Diseases (ICD-11), and its linkage to information on healthcare costs/ claims through a unified health claims exchange under the ABDM will help mitigate this limitation and further enhance the robustness of findings.

It is important to recognize that globally several countries resort to capitation as a method of payment for outpatient care (58). However, in our study, we did not find a significant relationship between the cost of providing outpatient care and the population being served. This finding is likely to be explained by the underlying context of healthcare delivery structure and financing, where norms for payment are not linked to the population size being served. Although the establishment of primary healthcare facilities is typically linked to the population size, there is no such linkage between the population and the norms for setting up district hospitals. Secondly, there is a wide variation between the population catered by different district hospitals, and there is no commensurate difference in the budgeting models. Therefore, with the introduction of the proposed payment reforms and better data availability such a relationship can be subsequently determined, and a more refined risk-adjusted capitation-based payment model can be evolved- that pays for the outpatient costs of all public health facilities in the districts, and not only the district hospital, which is the current practice in Thailand. It is also important to recognize that the reforms in financing may need to be accompanied by governance reforms towards more meaningful provider autonomy and decision space in public facilities, and innovations in organization of service delivery in order to be able to make use of the budgets in an efficient manner.

The Indian healthcare system is characterized by a unique set of features, including its public health infrastructure, the distribution of healthcare resources, the existing governance structures, and the specific challenges related to healthcare delivery (8, 41). These factors significantly influence the design and implementation of the proposed financing framework. While the framework presents a robust provider payment model tailored to India’s needs, its direct applicability to other LMICs may be constrained by the substantial variations in health systems across different countries. Therefore, the findings of the study should be interpreted and utilized in a manner that is both accurate and beneficial to the broader global health community. Each LMIC possesses distinct demand and supply side indicators, healthcare infrastructure, financing models, governance mechanisms, levels of resource availability, and socio-economic contexts that require careful consideration when adapting the framework to their specific circumstances.

To address these challenges, it is essential to underscore the importance of further research aimed at exploring how the principles of the responsive financing framework can be tailored to the diverse contexts of other LMICs. Such research could involve a combination of case studies, comparative analyses, and pilot implementations in various settings. Through these approaches, it would be possible to identify the necessary modifications to the framework, ensuring that it is appropriately adapted to local needs and conditions. Additionally, these studies would help validate the framework’s effectiveness and feasibility beyond the Indian context, providing valuable insights for policymakers and healthcare planners in other LMICs.

## Conclusion

The findings of our study provide an important foundation for reforming the financing mechanisms of district hospitals in India. Our research offers the first empirical evidence from a LMIC setting to outline the intricacies of blended payment reforms. However, we acknowledge that the implications of these findings are most immediately applicable within the specific context of India. The proposed method, by estimating the level of payment for each district hospital, sheds light on the complex challenges of financing healthcare in resource-constrained settings. We recognize that the broader application of this framework, particularly in contributing to UHC and comprehensive health system financing reforms, requires careful consideration of its limitations and the need for further validation.

Our study provides valuable insights into strategic purchasing of healthcare services, which can inform policymakers in making data-driven decisions for the procurement and contracting of healthcare services. By leveraging the proposed blended payment model, there is potential for improved resource allocation, enhanced service delivery, greater provider incentives, and ultimately better health outcomes for the populations served by district hospitals. We emphasize that while these findings are promising, their direct influence on UHC and similar systemic goals may be limited without exploring the proposed framework’s adaptability and effectiveness in different settings.

In conclusion, the study contributes significantly to the understanding of responsive financing mechanisms for district hospitals in India. It offers a model that can guide stakeholders involved in healthcare financing. Further studies are essential to assess its broader applicability and to refine the approach for different contexts, particularly in other Low- and Middle-Income Countries (LMICs). By presenting our conclusions with these considerations, we aim to propose a balanced perspective that reflects the study’s contributions while acknowledging the need for ongoing research and validation.

## Data Availability

The original contributions presented in the study are included in the article/Supplementary material, further inquiries can be directed to the corresponding author.
