# Supplementary material for: Framework for responsive financing of district hospitals of India
Source: Front Public Health. 2024 Oct 16;12:1398227. doi: 10.3389/fpubh.2024.1398227 (PMC11521915; doi:10.3389/fpubh.2024.1398227)
Supplement: Supplementary file 1 [file Data_Sheet_1.PDF]

Supplementary Material-1

Additional Details of Statistical Analysis

Data Analysis

Additional details of statistical analysis for estimation of budget for out-patient care

For determining the risk-adjusted global budget for outpatient care, the association between the cost of providing outpatient services and different factors influencing the healthcare needs and the resource requirement was assessed using multiple linear regression. A list of potential demand and supply side factors which are reported to influence the demand for healthcare services, or the cost of services was prepared.[17, 25-27] Finally, those factors for which valid data are generated regularly at the district level, and which are not amenable to reporting errors were chosen. For example, district-specific illness rate is likely to be an important determinant of outpatient care cost. However, a review of nationally representative surveys had suggested that the data on the illness rate is not available at the district level, and at a regular frequency.[27] Furthermore, due to the absence of electronic health records, the computation of illness rate is based on the data on self-reported illness collected during household surveys, which does not imply a definitive clinical diagnosis and can be confounded by positional objectivity.[28, 29] On the other hand, the district-wise data of IMR is available in India at a regular frequency.[25] IMR is also considered a reliable indicator of population health.[30, 31] In view of this, we used district specific IMR as an indicator of population health need.

Finally, the association of the outpatient care cost was assessed with the location of district hospital (type of city), district population size, IMR, proportional utilization in public hospital, proportion of population under 5 years and above 60 years of age (%), and bed strength of district hospital. To generate a sample of 100 health facilities, multivariate normal distribution (MVND)[32] was applied for simulating data for tier 1, tier 2 and tier 3, separately based on different characteristics of 27 district of CHSI dataset.[18, 33] All the parameters except type of city represented as continuous variables (outpatient cost, district population, infant mortality rate, utilization in public hospital, proportion of population under 5 years and above 60 years, and bed strength of the district hospital) followed normal distribution, so

MVND was used to simulate data using a vector of means of the individual covariates, and matrix consisting of the variances of the covariates along the main diagonal, and the covariance between each pair of covariates in the other matrix positions. As MVND defines the individual covariate distributions as well as maintains the systematic relationship between the covariates, thus, the simulated results approximate the original dataset of 27 district hospitals.

In order to explore the relationship between these factors and outpatient cost, we applied multiple linear regression model using ordinary least square method using equation 1. The multiple linear model is assumed to be:

$$Y = b_0 + b_1X_1 + b_2X_2 + \cdots + b_iX_i + e \qquad (1)$$

where Y is the outcome variable, X_i is the value of the ith predictor, and e is the error. Since the present analysis was using a perspective of developing norms for financing payments to be made to district hospitals, we included the full valuation of recurrent resources, as well as 20% of the capital costs. This was done since the majority of capital infrastructure in a district hospital exists already, and its expenditure in not repeated on an annual basis. However, 20% of capital cost was included to allow for maintenance of the existing capital infrastructure as well as its likely expansion. Normality of regress and error term for the model was checked using "Kolmogorov Smirnov Test" with insignificant p-values (0.854), and error term (0.701). The presence of homoscedasticity was checked using "Breusch-Pagan Test" with insignificant p-value (0.394), which failed to reject the null hypothesis of homoscedasticity. Thus, the assumptions of normality of regress and, the error term and presence of homoscedasticity were fulfilled for the model. There was no multicollinearity, with variance inflation value between 1 and 5.

Additional details of statistical analysis for budget impact assessment

The budgetary requirement for providing inpatient care was assessed at the specialty level, and computed as a product of the cost of delivering the services under HBPs as defined under *PM-JAY,* and the number of cases corresponding to each of the HBP in the preceding year.[33] However, the district hospitals also provide inpatient care for procedures which may not be part of the *PM-JAY* health benefit package. For such diseases/ procedures which are not covered under *PM-JAY*, the cost of providing inpatient care was assessed using the specialty-specific weighted average cost and volume of non-PM-*JAY* admissions, as explained in the equation below:

$$\textit{Estimated inpatient care payment} = \begin{cases} I_1 = \sum_{i=1}^{n}\sum_{j=1}^{m_i} c_{ij}\, a_{ij}\,, \textit{if package is included in PMJAY} \\ I_2 = \sum_{i=1}^{n}\left(\frac{\sum_{j=1}^{m_i} c_{ij}\, a_{ij}}{\sum_{j=1}^{m_i} a_{ij}}\right) * t_i, \qquad \textit{Else} \end{cases}$$

where n is total number of specialties, m_i is number of *PM-JAY* packages in i^{th} speciality, c_{ij} is the jth package cost in i^{th} specialty, a_{ij} is number of jth package claims in i^{th} speciality, t_i is number of non-PMJAY packages in i^{th} speciality,

$$\textit{IPD Cost} = I_1 + I_2$$

Details of model for estimation of responsive resource allocation for outpatient care services for a district hospital which is being upgraded to medical college

The financial pay-out for providing outpatient care for a hospital which is being upgraded to a medical college is estimated based on the multiple linear regression model using district specific indicators.

Supplementary Table 1: Model for estimation of responsive resource allocation for outpatient care services for a district hospital which is being upgraded to medical college

		Unstandardized Coefficient	SE	95% CIs
**Intercept**		91700000**	39000000	(14300000, 169000000)
**Type of City (Ref. Tier 1)**	**Tier 2**	-41500000**	13200000	(-67700000, -15300000)
	**Tier 3**	-75300000**	13700000	(-103000000, -48100000)
**District Population**		13276	145980	(-276653, 303205)
**Bed Strength**		122687**	21463	(80060,165314)
**Infant Mortality Rate**		401199*	213407	(-22645,825043)
**Population under 5 and above 60(%)**		-1033882	1339457	(-3694159,1626395)
**Utilization of Public health services (%)**		-53087	207728	(-465652, 359478)
R^2		0.6291		
**Adjusted** R^2		0.6009		

All coefficient values in ₹; SE= Standard error; CI= Confidence interval; * p value is significant when $\leqslant$0.1; ** p value is significant when $\leqslant$0.05

Supplementary Material 2					
**Annual financial payout for providing outpatient care in different district hospitals of India**					
**State Name**	**District**	**District Hospital (DH)**	**Payout for OPD Services (₹)**	**Lowest Payout for OPD Services (₹)**	**Highest Payout for OPD Services (₹)**
Andaman and Nicobar Islands	Nicobars	BJR Hospital	41115407	11498366	70732447
Andaman and Nicobar Islands	North & Middle Andaman	Dr R.P. Hospital	41798754	10421202	73176306
Andaman and Nicobar Islands	South Andaman	G.B. Pant Hospital	75250837	32260940	118240735
Andhra Pradesh	Anantapur	Ggh Anantapur	89501649	38431867	140571431
Andhra Pradesh	Chittoor	GovernmentMaternity Hospl.Th	66130874	25027075	107234674
Andhra Pradesh	Chittoor	Sri.Venkateshwara Ram Narayana Ruia Gen. Hospl.Th	137249432	69691135	204807730
Andhra Pradesh	Cuddapah	DH Proddutur	76699476	30261958	123136994
Andhra Pradesh	East Godavari	DH Rajahmundry	61391836	22115780	100667892
Andhra Pradesh	Guntur	DH Tenali	79059085	40285011	117733160
Andhra Pradesh	Krishna	DH Machilipatnam	69681361	27464630	111898093
Andhra Pradesh	Kurnool	DH Nandyal	91211150	46007908	136314392
Andhra Pradesh	Nellore	Government General Hospital Nellore	156892512	88075375	225609649
Andhra Pradesh	Prakasam	Rims Ongole Th	85879396	36988085	134770708
Andhra Pradesh	Srikakulam	Rims Srikakulam Th	87973511	37822771	138124251
Andhra Pradesh	Visakhapatnam	King George Hospital Th	224096346	123609652	324583040
Andhra Pradesh	Vizianagaram	DH Vizianagaram	74394139	28320704	120467573
Andhra Pradesh	West Godavari	DH Eluru	70983108	27983489	113982728
Arunachal Pradesh	East Siang	GH Pasighat	47499138	14533579	80464698
Arunachal Pradesh	Lohit	GH Tezu	40643998	10124512	71163484
Arunachal Pradesh	Lower Dibang Valley	DH Roing	39585539	9784414	69386665
Arunachal Pradesh	Papum Pare	Tomo Riba Institute Of Medical Science & Hospital	57956274	20828200	95084348
Arunachal Pradesh	Tawang	DH Tawang	37064805	8084466	66045143
Arunachal Pradesh	West Siang	General Hospital Aalo	38374186	9465165	67283206
Assam	Baksa	Dr Ravi Boro Civil Hospital Baksa	49028906	13507521	84550292
Assam	Barpeta	Barpeta Civil Hospital Kalgachia	44819962	11318703	78321221
Assam	Bongaigaon	Bongaigaon Ch	55238670	17822930	92654410
Assam	Cachar	S.M.Deb Civil Hospital Silchar	67473852	21104805	113842900
Assam	Chirang	J.S.B Civil Hospital Chirang	49608422	14351935	84864909
Assam	Darrang	Mangaldai Civil Hospital	66727188	23220004	110234372
Assam	Dhemaji	DHemaji Civil Hospital	59483498	19514863	99452133
Assam	Dhubri	DHubri Civil Hospital	61803188	21257362	102349014
Assam	Dima Hasao	Haflong Civil Hospital	71097835	23653444	118542225
Assam	Goalpara	200 Bedded Civil Hospital	59199694	20219644	98179744
Assam	Golaghat	Kushal Konwar Civil Hospital	78829474	30599759	127059189

Assam	Hailakandi	S.K.Roy Civil Hospital	70275031	22630269	117919793
Assam	Kamrup Metropolitan	Sonapur District Hospital	45152372	11553435	78751309
Assam	Kamrup	Trb Civil Hospital	45110127	11332121	78888133
Assam	Karbi Anglong	Diphu Civil Hospital	70292843	24641227	115944459
Assam	Karimganj	Karimganj Civil Hospital	76642322	26538172	126746471
Assam	Kokrajhar	Rnb Civil Hospital Kokrajhar	60388246	20693385	100083106
Assam	Lakhimpur	North Lakhimpur Civil Hospital	65792918	23256567	108329270
Assam	Morigaon	Morigaon Civil Hospital	65384012	21662255	109105769
Assam	Nagaon	B.P. Civil Hospital	79269990	30305049	128234930
Assam	Nalbari	Smk Civil Hospital	61922745	22020680	101824810
Assam	Sivasagar	Sivasagar Civil Hospital	78195906	30020066	126371745
Assam	Sonitpur	Kanaklata Civil Hospital	69309610	25517071	113102148
Assam	Tinsukia	LGB Civil Hospital	57743322	18616776	96869868
Assam	Udalguri	Udalguri Civil Hospital	57762307	18031365	97493250
Bihar	Araria	Sardar Hospital Araria	53570261	15931072	91209450
Bihar	Arwal	Sadar Hospital Arwal	52510530	15447334	89573725
Bihar	Aurangabad	Sadar Hospital Aurangabad	55214138	16872565	93555712
Bihar	Banka	Sadar Hospital Banka	55878958	17342029	94415886
Bihar	Begusarai	Sadar Hospital Begusarai	52302309	15589263	89015355
Bihar	Bhagalpur	LNJP Sadar Hospital Bhagalpur	47048802	11900376	82197228
Bihar	Bhojpur	Sadar Hospital Ara Bhojpur	56328380	18052799	94603961
Bihar	Buxar	Sadar Hospital Buxar	52494904	15502449	89487360
Bihar	East Champaran	Sadar Hospital Motihari Purbi Champaran	58408141	19086240	97730043
Bihar	Gaya	Sadar Hospital Pilgrim Gaya	49835627	13624594	86046661
Bihar	Gopalganj	Sadar Hospital Gopalganj	51582164	15240880	87923449
Bihar	Jamui	Sadar Hospital Jamui	53060881	15728040	90393723
Bihar	Jehanabad	Sadar Hospital Jehanabad	52494904	15502449	89487360
Bihar	Kaimur	Sadar Hospital Bhabua Kaimur	54314480	16268603	92360357
Bihar	Katihar	Sadar Hospital Katihar	57729784	17997954	97461614
Bihar	Khagaria	Sadar Hospital Khagaria	52042123	15321976	88762269
Bihar	Kishanganj	Sadar Hospital Kishanganj	53683456	15976190	91390722
Bihar	Lakhisarai	Sadar Hospital Lakhisarai	49814098	13922726	85705469
Bihar	Madhepura	Sadar Hospital Madhepura	51278461	14608640	87948283
Bihar	Madhubani	Sadar Hospital Madhubani	55480229	16896835	94063623
Bihar	Munger	Sadar Hospital Munger	56653613	18386908	94920318
Bihar	Muzaffarpur	Sadar Hospital Muzaffarpur	57236487	18701024	95771951
Bihar	Nalanda	Sadar Hospital Biharsharif Nalanda	69922920	26538535	113307306
Bihar	Nawada	Sadar Hospital Nawada	51173715	14607784	87739645
Bihar	Purnia	Sadar Hospital Purnia	73148989	27824404	118473575
Bihar	Rohtas	Sadar Hospital Rohtas Sasaram	53230674	15795717	90665632
Bihar	Saharsa	Sadar Hospital Saharsa	68286219	25497664	111074774
Bihar	Samastipur	Sadar Hospital Samastipur	51524294	15074681	87973908
Bihar	Saran	Sadar Hospital Saran	53689817	16694389	90685246
Bihar	Sheikhpura	Sadar Hospital Sheikhpura	50923250	14528400	87318100

Bihar	Sheohar	Sadar Hospital Sheohar	51770943	14723146	88818739
Bihar	Sitamarhi	Sadar Hospital Sitamarhi	53654292	15412482	91896103
Bihar	Siwan	Sadar Hospital Siwan	50910169	14870794	86949543
Bihar	Supaul	Sadar Hospital Supaul	51521750	14787401	88256098
Bihar	Vaishali	Sadar Hospital Hajipur Vaishali	54175122	16908273	91441972
Bihar	West Champaran	Sadar Hospital M.J.K Bettiah Paschim Champaran	85639467	36279026	134999909
Chandigarh	Chandigarh	GMSH 16	106053484	58017235	153989734
Chhattisgarh	Balod	DH Balod	55324789	16630404	94019175
Chhattisgarh	Baloda Bazar	DH Baloda Bazar	55268192	16607845	93928539
Chhattisgarh	Bemetara	DH Bemetara	53457880	15068377	91847384
Chhattisgarh	Bilaspur	Bilaspur DH	80281790	39157009	121306572
Chhattisgarh	Dantewada	Dantewada	62008864	20828154	103189575
Chhattisgarh	Dhamtari	DHamtari	65106569	23085235	107127904
Chhattisgarh	Durg	District Hospital Durg	80744989	33101299	128388679
Chhattisgarh	Gariaband	DH Gariaband	53967260	15271409	92663111
Chhattisgarh	Janjgir-Champa	District Hospital	54757540	16261173	93253907
Chhattisgarh	Jashpur	Jashpur	57245751	17927710	96563793
Chhattisgarh	North Bastar Kanker	Kanker DH	65482854	21906127	109059580
Chhattisgarh	Kawardha	District Hospital Kawardha	59286628	18209541	100363716
Chhattisgarh	Kondagaon	Ravindra Tagore DH Kondagaon	69032883	23382465	114683302
Chhattisgarh	Korba	Indira Gandhi Dstt Hospital Korba	56685679	17459102	95912255
Chhattisgarh	Mahasamund	Mahasamund	60551677	20042863	101060492
Chhattisgarh	Mungeli	DH Mungeli	54080455	15316527	92844383
Chhattisgarh	Narayanpur	Narayanpur	64720007	20375214	109064801
Chhattisgarh	Raipur	Raipur	70217479	33550599	106784359
Chhattisgarh	Sukma	DH Sukma	59343226	18232100	100454352
Dadra and Nagar Haveli	Dadra & Nagar Haveli	Shri Vinoba Bhave Civil Hospital	66877638	25651886	108103390
Daman and Diu	Daman	Government Hospital Daman	50753099	16239516	85266682
Daman and Diu	Diu	Government Hospital Diu	42817513	10827266	74807759
Delhi	Central	Aruna Asaf Ali Hospital	100952281	88590592	113313970
Delhi	Central	Girdhari Lal Maternity Hospital	96496231	85792092	107200370
Delhi	Central	Kasturba Hospital	127955944	105549502	150362386
Delhi	East	Lal Bahadur Shastri Hospital	104153460	90704889	117602032
Delhi	North	Babu Jagjeevan Ram Memorial Hospital Jahgirpuri	96084422	85689293	106479550
Delhi	North	Maharishi Valmiki Hospital	100540472	88487793	112593150
Delhi	North	Satyawati Raja Harishchandra Hospital	104996522	91286293	118706750
Delhi	North East	DH Jpc Hospital	107183574	92607869	121759280
Delhi	North West	Deep Chand Bandhu Hospital	104883326	91241175	118525478
Delhi	North West	Sanjay Gandhi Memorial Hospital Mangolpuri	113795426	96838175	130752678
Delhi	North West	Bhagwan Mahavir Hospital Pitampura	116023451	98237425	133809478
Delhi	South	Pt. Madan Mohan Malviya Hospital	96861164	86060235	107662093
Delhi	South West	Rao Tula Ram Hospital	96367410	85802088	106932732
Delhi	West	Guru Govind Singh Govt Hospital	96027824	85666734	106388914
Delhi	West	Acharya Shree Bhikshu Hospital	103157504	90144334	116170674

Delhi	West	Deendayal Upadhyay Hospital	144153164	115890534	172415794
Delhi	Shahdara	Hedgewar Hospital	104996522	91286293	118706750
Delhi	Shahdara	DH SDN Hospital	120147092	100801193	139492990
Goa	North Goa	North Goa District Hospital	53772676	18772170	88773183
Goa	South Goa	South Goa District Hospital	52874290	18311847	87436732
Gujarat	Amreli	General Hospital Amreli	58944597	20526916	97362277
Gujarat	Anand	S.S.Hospital Petlad	75476433	36035252	114817614
Gujarat	Banaskantha	General Hospital Palanpur	77817892	30094318	125541466
Gujarat	Bharuch	General Hospital Bahruch	62298214	21659149	102937280
Gujarat	Botad	Botad	48720571	13261938	84179205
Gujarat	Chhotaudepur	Chhotaudepur	57815088	17623004	98007172
Gujarat	Dahod	General Hospital Dahod	97844532	49940082	145648981
Gujarat	Devbhumi Dwarka	Jam Khambhalia	51913759	16293189	87534329
Gujarat	Kheda	General Hospital Nadiad	60615452	19966045	101264859
Gujarat	Mahesana	General Hospital Mehsana	62315112	21747674	102882550
Gujarat	Mahisagar	Lunawada	51321521	14012376	88630665
Gujarat	Morbi	Morbi	59677822	20532905	98822740
Gujarat	Narmada	General Hospital Rajpipla	54820042	16040715	93599369
Gujarat	Navsari	M.G.G.Hospital Navsari	57968083	20342166	95593999
Gujarat	Panchmahal	General Hospital Godhra	70450013	26012516	114887510
Gujarat	Porbandar	Bhavsinhji General Hospital	58325839	20709686	95941992
Gujarat	Rajkot	PK General Hospital	64649375	31637939	97560811
Gujarat	Surendranagar	M.G. General Hospital	52989930	15904235	90075626
Gujarat	Tapi	General Hospital Vyara	64413044	23013280	105812807
Gujarat	Dang	General Hospital Dang	60558039	20761062	100355015
Gujarat	Vadodara	Jamnabai General Hospital	80657717	39756694	121458739
Haryana	Ambala	Civil Hospital	57841349	19678225	96004473
Haryana	Bhiwani	Civil Hospital	69300346	26290385	112310306
Haryana	Faridabad	B.K. Civil Hospital	79299372	39215276	119283468
Haryana	Fatehabad	Civil Hospital Fatehabad	52777893	15615244	89940541
Haryana	Hisar	Civil Hospital	59041709	19665932	98417487
Haryana	Jhajjar	Civil Hospital Jhajjar	52551502	15525008	89577996
Haryana	Jind	Civil Hospital Jind	52452660	15281135	89624184
Haryana	Kaithal	IGMS Civil Hospital	58839393	19564844	98113942
Haryana	Kurukshetra	LNJP Civil Hospital	50027408	14355357	85699459
Haryana	Mahendragarh	Civil Hospital	48872651	14058666	83686637
Haryana	Mewat	Civil Hospital Mandikhera	56463463	16020992	96905933
Haryana	Palwal	Civil Hospital	59796008	18412573	101179443
Haryana	Panchkula	Civil Hospital	66470461	25162430	107778491
Haryana	Panipat	Civil Hospital	50527066	14370486	86683645
Haryana	Rewari	Civil Hospital Rewari	53014005	15893383	90134626
Haryana	Rohtak	Civil Hospital	52494904	15502449	89487360
Haryana	Sirsa	Civil Hospital Sirsa	52239807	15809721	88669893
Haryana	Sonipat	Civil Hospital Sonepat	61803188	21257362	102349014

Himachal Pradesh	Bilaspur	Bilaspur RH	88480922	44306249	132555596
Himachal Pradesh	Chamba	Chamba RH MCH Centre	68168392	25839203	110497580
Himachal Pradesh	Hamirpur	Hamirpur RH	86441676	42389265	130394086
Himachal Pradesh	Kangra	DHaramshala ZH	58668785	20314743	97022826
Himachal Pradesh	Kullu	Kullu RH	69753127	26470858	113035397
Himachal Pradesh	Lahul and Spiti	Keylong RH	43837086	10415753	77258420
Himachal Pradesh	Mandi	Mandi ZH	65451702	24756366	106147038
Himachal Pradesh	Shimla	DDU ZH	82868029	41884996	123751062
Himachal Pradesh	Sirmaur	Nahan RH	63089767	22792553	103386981
Himachal Pradesh	Solan	Solan RH	53342240	17475989	89208491
Himachal Pradesh	Una	Una RH	54954866	18527711	91382022
Jammu and Kashmir	Anantnag	MCH Anantnag	51857976	15453053	88262900
Jammu and Kashmir	Anantnag	DH Anantnag	57918204	19259013	96577396
Jammu and Kashmir	Badgam	District Hospital Budgam	47022640	12585165	81460116
Jammu and Kashmir	Bandipore	Bandipora	45704810	11160203	80249418
Jammu and Kashmir	Baramulla	Baramula	62425763	21505512	103346013
Jammu and Kashmir	Doda	Doda	55182072	17800371	92563774
Jammu and Kashmir	Ganderbal	District Hospital	47713621	12369840	83057403
Jammu and Kashmir	Jammu	Gandhinagar Hospital	69657863	34043207	105172520
Jammu and Kashmir	Jammu	Sarwal Hospital	62795546	29733517	95757576
Jammu and Kashmir	Kathua	Kathua	54389705	17484544	91294866
Jammu and Kashmir	Kulgam	Kulgam	43395298	10566823	76223774
Jammu and Kashmir	Kupwara	DH Handwara	48435038	12861862	84008214
Jammu and Kashmir	Pulwama	Pulwama DH	46471016	12160819	80781214
Jammu and Kashmir	Ramban	Ramban	43525391	10700466	76350317
Jammu and Kashmir	Reasi	DH Reasi	43341245	10831543	75850947
Jammu and Kashmir	Samba	Samba	47444171	12875866	82012475
Jammu and Kashmir	Shopian	Shopain	43604791	10568534	76641049
Jammu and Kashmir	Srinagar	District Hospital Jnlm	71390862	35040671	107641054
Jammu and Kashmir	Udhampur	Udhampur	56935786	19317280	94554292
Jharkhand	Bokaro	Bokaro Sadar Hospital	67557387	32490321	102524453
Jharkhand	Chatra	Chatra Sadar Hospital	47404014	11980616	82827411
Jharkhand	Deoghar	Deoghar Sadar Hospital	52664697	15570126	89759269
Jharkhand	Dumka	Dumka Sadar Hospital	53271647	15873391	90669903
Jharkhand	Garhwa	Garhwa Sadar Hospital	55041801	16517608	93565993
Jharkhand	Giridih	Giridih Sadar Hospital	51872330	15254299	88490361
Jharkhand	Godda	Godda Sadar Hospital	53683456	15976190	91390722
Jharkhand	Gumla	Gumla Sadar Hospital	63368481	21513212	105223751
Jharkhand	Hazaribagh	Hazaribagh Sadar Hospital	71277905	27610250	114945561
Jharkhand	Jamtara	Jamtara Sadar Hospital	54023042	16111544	91934540
Jharkhand	Khunti	Khunti Sadar Hospital	50401604	13850185	86953024
Jharkhand	Koderma	Kodrma Sadar Hospital	50910169	14870794	86949543
Jharkhand	Latehar	Latehar Sadar Hospital	52325926	14617195	90034658
Jharkhand	Lohardaga	Lohardaga Sadar Hospital	51095130	13922140	88268120

Jharkhand	Pakur	Pakur Sadar Hospital	53004284	15705481	90303087
Jharkhand	Palamu	Palamau Sadar Hospital	63840705	22069490	105611921
Jharkhand	West Singhbhum	Pashchimi Singhbhum Sadar Hospital	54702215	16382254	93022176
Jharkhand	East Singhbhum	Purbi Singhbhum Sadar Hospital	47004927	13314216	80695639
Jharkhand	Ramgarh	Ramgarh Sadar Hospital	44317759	11016294	77619225
Jharkhand	Ranchi	Ranchi Sadar Hospital	77488246	38493385	116383106
Jharkhand	Sahebganj	Sahibganj Sadar Hospital	50118616	13737390	86499842
Jharkhand	Saraikela Kharsawan	Saraikela Sadar Hospital	48108990	13345330	82872650
Jharkhand	Simdega	Simdega Sadar Hospital	55607778	16743199	94472356
Karnataka	Bagalkot	Bagalkote District Hospital FRU	68790966	26087353	111494580
Karnataka	Bengaluru Urban	HSIS Goshiya	99225186	87350111	111100262
Karnataka	Bengaluru Urban	Indiranagar General Hospital	106354866	91827711	120882022
Karnataka	Bengaluru Urban	Jayanagar General Hospital	113930151	96585161	131275142
Karnataka	Bengaluru Urban	Vanivilas Hospital	136299522	110633631	161965414
Karnataka	Bengaluru Urban	Victoria Hospital	156619110	123394791	189843430
Karnataka	Bengaluru Urban	Kc General Hospital	120614226	100782911	140445542
Karnataka	Bengaluru Urban	Bowring Lady Curzon	180325296	138282811	222367782
Karnataka	Belagavi	Belgaum District Hospital	120576390	66709425	174343356
Karnataka	Ballari	Bellary District Hospital FRU	65071502	22764545	107378459
Karnataka	Ballari	Vims Bellary Medical College	136992149	67932335	206051963
Karnataka	Bidar	Bidar District Hospital	77914289	32790920	123037657
Karnataka	Bijapur	Bijapur District Hospital FRU	69032525	25161258	112903793
Karnataka	Chamarajanagar	Chamarajnagar District Hospital FRU	66244070	25072193	107415946
Karnataka	Chikballapur	Chikkaballapur District Hospital FRU	53909032	16884003	90934060
Karnataka	Chikkamagaluru	Chickmagalur District Hospital FRU	71873503	29360766	114386241
Karnataka	Chitradurga	Chitradurga District Hospital FRU	82159116	34482853	129835380
Karnataka	Dakshina Kannada	Lady Goshan Hospital Mangalore DH FRU	54636452	19873018	89399886
Karnataka	Dakshina Kannada	Wenlock Hospital Mangalore DH	111050045	55302028	166798062
Karnataka	Davanagere	Davangere Women And Children DH FRU	50570582	14735439	86405726
Karnataka	Davanagere	Davanagere District Hospital	124541012	61190539	187891486
Karnataka	Dharwad	DHarwad District Hospital FRU	80529353	40727907	120230799
Karnataka	Dharwad	Hubli Kims District Hospital	165194303	93899407	236389199
Karnataka	Gadag	Gadag District Hospital FRU	68353353	24890549	111816157
Karnataka	Gulbarga	Gulbarga District Hospital FRU	107138105	57672544	156503666
Karnataka	Hassan	Hassan District Hospital	103971416	49311211	158631622
Karnataka	Haveri	Haveri District Hospital FRU	69073955	26200148	111947761
Karnataka	Kodagu	Kodagu District Hospital FRU	69595242	28657156	110533328
Karnataka	Kolar	Kolar District Hospital FRU	74759986	30511280	119008692
Karnataka	Koppal	Koppal District Hospital FRU	74054552	28185349	119923756
Karnataka	Mandya	Mandya District Hospital	97436420	44661693	150211146
Karnataka	Mysuru	Cheluvamba Hospital Mysore DH	91223058	48261884	134084232
Karnataka	Mysuru	KR Hospital Mysore DH	145586868	82403584	208670152
Karnataka	Raichur	Raichur District Hospital	103110543	46718849	159502237
Karnataka	Ramanagara	Ramanagara District Hospital FRU	48929249	14081225	83777273

Karnataka	Shivamogga	Shimoga District Hospital	121116444	60234502	181998386
Karnataka	Tumakuru	Tumkur District Hospital FRU	76797503	31323407	122271599
Karnataka	Udupi	Udupi District Hospital FRU	54034135	19183097	88885173
Karnataka	Uttara Kannada	Uttara Kannada District Hospital FRU	64783523	24817199	104749847
Karnataka	Yadgir	Yadgir District Hospital FRU	52721295	15592685	89849905
Kerala	Alappuzha	W And C Hospital Alappuzha	51197073	18154519	84239628
Kerala	Alappuzha	General Hospital Alappuzha	64119618	26270169	101969068
Kerala	Ernakulam	DH Aluva	48362099	16452004	80272194
Kerala	Ernakulam	GH Ernakulam	85079951	39511644	130648258
Kerala	Idukki	District Hospital Thodupuzha	44021332	13024686	75017978
Kerala	Kannur	GH Thalassery	94974231	52435680	137412782
Kerala	Kasaragod	DH Kanhangad	61760228	24593635	98926820
Kerala	Kollam	W&C Hospital Kollam	70467228	37187570	103646887
Kerala	Kollam	DH Kollam	93995172	51963650	135926695
Kerala	Kottayam	General Hospital Kottayam	60837767	24144164	97531369
Kerala	Kozhikode	General Hospital Calicut	93321904	51449924	135093885
Kerala	Malappuram	DH Tirur	61319016	31312431	91225602
Kerala	Malappuram	GH Manjeri	91352793	50174321	132431266
Kerala	Palakkad	W & C Palakkad	51770227	18280733	85259721
Kerala	Palakkad	District Hospital Palakkad	77971801	34735913	121207689
Kerala	Pathanamthitta	General Hosp Pathanamthitta	65518935	26889261	104148610
Kerala	Thiruvananthapuram	District Model Hospital Peroorkada	72816898	38676201	106857594
Kerala	Thiruvananthapuram	General Hospital Thiruvananthapuram	112653985	63694791	161513178
Kerala	Thiruvananthapuram	W&C Hospital Thiruvananthapuram	84224386	45840361	122508410
Kerala	Thrissur	GH Thrissur	66620672	34979614	98161730
Kerala	Wayanad	DH Mananthavady	57341333	21441889	93240777
Ladakh	Kargil	Kargil	61804003	20439786	103168221
Ladakh	Leh	Leh DH	63655744	23018144	104293344
Lakshadweep	Lakshadweep	Indira Gandhi Hospital	33549843	6928819	60170867
Madhya Pradesh	Agar Malwa	DH Agar	56739732	17194381	96285082
Madhya Pradesh	Alirajpur	DH Alirajpur	59102482	18340618	99864346
Madhya Pradesh	Anuppur	DH Anuppur	58890444	18051627	99729262
Madhya Pradesh	Ashoknagar	DH Ashoknagar	59456421	18277218	100635625
Madhya Pradesh	Balaghat	DH Balaghat	71790644	27282985	116298304
Madhya Pradesh	Barwani	DH Barwani	71620851	27215308	116026395
Madhya Pradesh	Betul	DH Betul	67885403	25726407	110044399
Madhya Pradesh	Bhind	DH Bhind	79812350	31502709	128121991
Madhya Pradesh	Bhopal	DH Bhopal J.P	86413884	44709207	128018560
Madhya Pradesh	Burhanpur	DH Burhanpur	62255970	21437835	103074104
Madhya Pradesh	Chhatarpur	DH Chhatarpur	82487610	31546655	133428564
Madhya Pradesh	Chhindwara	DH Chhindwara	77023894	31413644	122634144
Madhya Pradesh	Damoh	DH Damoh	83110184	31794805	134425564
Madhya Pradesh	Datia	DH Datia	79585959	31412472	127759445
Madhya Pradesh	Dewas	DH Dewas	77137089	31458762	122815417

Madhya Pradesh	Dhar	DH DHar	70432300	26741567	114123032
Madhya Pradesh	Dindori	DH Dindori	62965479	19675882	106255075
Madhya Pradesh	Guna	DH Guna	85570147	34820068	136320225
Madhya Pradesh	Gwalior	DH Gwalior	83487602	40884649	125990554
Madhya Pradesh	Harda	DH Harda	53909847	16066426	91753267
Madhya Pradesh	Hoshangabad	DH Hoshangabad	68677771	26042235	111313307
Madhya Pradesh	Indore	DH Indore	64444514	31249571	97539457
Madhya Pradesh	Jabalpur	DH Jabalpur	111186063	58059154	164212971
Madhya Pradesh	Jhabua	DH Jhabua	64916062	22498113	107334010
Madhya Pradesh	Katni	DH Katni	74424475	26288042	122560909
Madhya Pradesh	Khandwa	DH Khandwa	81664905	33263490	130066321
Madhya Pradesh	Khargone	DH Khargone	71734047	27260426	116207667
Madhya Pradesh	Mandla	DH Mandla	76941035	29335863	124546207
Madhya Pradesh	Mandsaur	DH Mandsaur	90916592	38995845	142837339
Madhya Pradesh	Morena	DH Morena	77846598	29696809	125996388
Madhya Pradesh	Narsinghpur	DH Narsinghpur	72186828	27440899	116932758
Madhya Pradesh	Neemuch	DH Neemuch	64463280	22317640	106608920
Madhya Pradesh	Panna	DH Panna	76801579	27235524	126367633
Madhya Pradesh	Raisen	DH Raisen	62765349	21640867	103889831
Madhya Pradesh	Rajgarh	DH Rajgarh	74507334	28365822	120648846
Madhya Pradesh	Ratlam	DH Ratlam	104281143	55306944	153155343
Madhya Pradesh	Rewa	DH Rewa	62286306	19405173	105167440
Madhya Pradesh	Sagar	DH Sagar	78921955	30125432	127718477
Madhya Pradesh	Satna	DH Satna	89984767	36579678	143389857
Madhya Pradesh	Sehore	DH Sehore	63613499	22796830	104430169
Madhya Pradesh	Seoni	DH Seoni	79740584	32496481	126984687
Madhya Pradesh	Shahdol	DH Shahdol	81299058	31072914	131525202
Madhya Pradesh	Shajapur	DH Shajapur	60614636	20783621	100445652
Madhya Pradesh	Sheopur	DH Sheopur	66587732	21119665	112055799
Madhya Pradesh	Shivpuri	DH Shivpuri	78129587	29809604	126449569
Madhya Pradesh	Sidhi	DH Sidhi	81751840	31253387	132250292
Madhya Pradesh	Singrauli	DH Singrauli	64238519	20592250	107884789
Madhya Pradesh	Tikamgarh	DH Tikamgarh	72273763	25430796	119116729
Madhya Pradesh	Ujjain	DH Ujjain	119728240	65553462	173803018
Madhya Pradesh	Umaria	DH Umaria	66304743	21006869	111602617
Madhya Pradesh	Vidisha	DH Vidisha	76827840	29290745	124364934
Maharashtra	Ahmednagar	Ahmednagar	60474464	22240868	98708060
Maharashtra	Amravati	District General Hospital Amravati	89365870	46887760	131743981
Maharashtra	Amravati	District Women Hospital Amravati	72432880	36253460	108512301
Maharashtra	Beed	District Hospital Beed	65253203	25086198	105420208
Maharashtra	Bhandara	Bhandara	83596046	35709915	131482176
Maharashtra	Buldhana	DH Buldana	65986428	25092186	106880670
Maharashtra	Gadchiroli	District Hospital Gadchiroli	66607273	24235480	108979067
Maharashtra	Hingoli	DH Hingoli	47967088	13697721	82236456

Maharashtra	Jalgaon	District Hospital Jalgaon	87995260	45871159	130019360
Maharashtra	Jalna	Women Hospital Jalna	44345651	11436362	77254940
Maharashtra	Jalna	District Hospital Jalna	56822591	19272162	94373020
Maharashtra	Nandurbar	Nandurbar	60501441	20738503	100264379
Maharashtra	Nashik	District Hospital Nashik	102275334	55345804	149104865
Maharashtra	Osmanabad	District Hospital Osmanabad	56918073	20046331	93789815
Maharashtra	Osmanabad	WH Osmanabad	41232777	10195611	72269943
Maharashtra	Parbhani	General Hospital Parbhani	75577700	30959895	120195506
Maharashtra	Parbhani	Women Hospital Parbhani	44741834	11594275	77889394
Maharashtra	Pune	Aundh	115321477	98121207	132521746
Maharashtra	Raigarh	Alibag	69917831	25963975	113871687
Maharashtra	Ratnagiri	District Hospital Ratnagiri	51841993	17286961	86397025
Maharashtra	Satara	Lt Karntisigh Nana Patil Civil Hospital Satara	55245489	19502346	90988631
Maharashtra	Sindhudurg	Sindhudurg	51672200	17219283	86125116
Maharashtra	Thane	District Hospital Thane	60264971	22239157	98290785
Maharashtra	Wardha	Wardha	64470099	23997056	104943141
Maharashtra	Washim	Washim	55860430	18888657	92832202
Manipur	Bishnupur	Bishnupur District Hospital	39039820	9117052	68962588
Manipur	Chandel	Chandel District Hospital	38526624	8483100	68570147
Manipur	Churachandpur	Churachandpur District Hospital	48621371	14796858	82445884
Manipur	Senapati	Senapati District Hospital	40171774	9568234	70775314
Manipur	Tamenglong	Tamenglong District Hospital	42206747	10093082	74320412
Manipur	Thoubal	Thoubal District Hospital	39495146	9584804	69405488
Manipur	Ukhrul	Ukhrul District Hospital	39209613	9184729	69234497
Meghalaya	East Garo Hills	Williamnagar Civil Hospital	46438950	13088625	79789276
Meghalaya	East Khasi Hills	Ganesh Das Hospital	75776200	30630064	120922336
Meghalaya	East Khasi Hills	Shillong Civil Hospital	94848094	42607644	147088544
Meghalaya	Ri Bhoi	Nongpoh DH	51026724	14385615	87667832
Meghalaya	West Garo Hills	Tura Maternity And Child Hospital	43624234	10944339	76304128
Meghalaya	West Garo Hills	Tura Civil Hospital	56992384	19339839	94644928
Meghalaya	West Jaintia Hills	Jowai Civil Hospital	59320424	18386591	100254256
Meghalaya	West Khasi Hills	Nongstoin DH	48872651	14058666	83686637
Meghalaya	West Khasi Hills	Mairang DH	48872651	14058666	83686637
Mizoram	Aizawl	Aizawl Civil Hospital	64178661	23839998	104517325
Mizoram	Champhai	Champhai DH	44994844	11960940	78028749
Mizoram	Kolasib	Kolasib DH	45081421	11729630	78433211
Mizoram	Lawngtlai	Lawngtlai DH	46273332	11673074	80873590
Mizoram	Lunglei	Lunglei DH	53553820	16803763	90303877
Mizoram	Mamit	Mamit DistrictHospital	44485008	10796691	78173324
Mizoram	Saiha	Saiha DH	49291180	13100872	85481489
Mizoram	Serchhip	Serchhip DH	43826550	11045427	76607673
Nagaland	Dimapur	District Hospital	53385299	16879725	89890873
Nagaland	Mokokchung	Ongpangkong DH	52649529	16586457	88712601
Nagaland	Wokha	Wokha DH	43850624	11034575	76666674

Odisha	Angul	Angul	63486309	21171673	105800944
Odisha	Balangir	Balangir	71859866	26001933	117717798
Odisha	Balasore	Balasore	73345859	29129726	117561992
Odisha	Bargarh	Bargarh	61690808	20394667	102986948
Odisha	Boudh	Boudh	58719379	17840310	99598448
Odisha	Bhadrak	Bhadrak	66480997	24532756	108429238
Odisha	Cuttack	City Hospital	68306695	33402412	103110979
Odisha	Deogarh	Deogarh	61895212	19821819	103968604
Odisha	Dhenkanal	DHenkanal	70947583	26701582	115193585
Odisha	Gajapati	Paralakhemundi	61455511	19298952	103612070
Odisha	Ganjam	City Hospital	60829577	19581099	102078055
Odisha	Jagatsinghapur	Jagatsinghpur	49322073	14769436	83874711
Odisha	Jajapur	Jajpur	70934502	27043976	114825029
Odisha	Jharsuguda	Jharsuguda	55618771	17074742	94162801
Odisha	Kalahandi	Bhawanipatna	71701424	24487005	118915843
Odisha	Kandhamal	Phulbani	69611126	24083238	115139014
Odisha	Kendrapara	Kendrapada	58131514	19691644	96571385
Odisha	Kenduajhar	Keonjhar	63736774	21251058	106222490
Odisha	Khordha	Capital Hospital	98079709	45429289	150730130
Odisha	Khordha	Khordha	50934700	15821159	86048242
Odisha	Koraput	DHh Koraput	74941031	26391696	123490366
Odisha	Malkangiri	Malkangiri	67514009	22000055	113027964
Odisha	Mayurbhanj	Baripada	67270820	24561303	109980337
Odisha	Nabarangpur	Nabarangpur	68178829	22469519	113888138
Odisha	Nayagarh	Nayagarh	58724925	19376086	98073765
Odisha	Nuapada	Nuapada	62314198	19825241	104803154
Odisha	Puri	Puri	71223037	28692548	113753527
Odisha	Rayagada	Rayagada	68536128	21875822	115196433
Odisha	Sambalpur	Sambalpur	67144087	23897363	110390810
Odisha	Sonepur	Subarnapur	58876091	18250381	99501801
Odisha	Sundargarh	Rgh Rourkela	76514873	29431819	123597927
Odisha	Sundargarh	Sundargarh	66176837	22939299	109414375
Puducherry	Karaikal	Government General Hospital	79961984	34752167	125171802
Puducherry	Mahe	Government General Hospital	43937300	13543275	74331325
Puducherry	Puducherry	RGGW & CH	90712864	48876429	132449298
Puducherry	Yanam	Government General Hospital	43495870	11915552	75076188
Punjab	Amritsar	Amritsar DH	72960430	36688657	109132202
Punjab	Barnala	Barnala DH	54502900	17529662	91476138
Punjab	Bathinda	Bathinda DH	56426407	19114248	93738565
Punjab	Faridkot	Faridkot DH	49212238	14194021	84230454
Punjab	Fatehgarh Sahib	Fatehgarh Sahib DH	43581174	11540602	75621746
Punjab	Fazilka	Fazilka DH	45739063	12298471	79179656
Punjab	Ferozepur	Ferozepur DH	53088773	16148108	90029438
Punjab	Gurdaspur	Gurdaspur DH	46286055	13109473	79462637

Punjab	Hoshiarpur	Hoshiarpur DH	54162499	18211884	90113113
Punjab	Jalandhar	Jalandhar DH	96683513	51665202	141601825
Punjab	Kapurthala	Kapurthala DH	49070336	14546411	83594260
Punjab	Ludhiana	Ludhiana DH	78689520	40403525	116875516
Punjab	Mansa	Mansa DH	48589663	13945871	83233455
Punjab	Moga	Moga DH	51673830	15584130	87763530
Punjab	Sahibzada Ajit Singh Nagar	Mohali DH	53143740	17805820	88481660
Punjab	Sri Mukhtar Sahib	Muktsar DH	47287916	13427011	81148820
Punjab	Shahid Bhagat Singh Nagar	Nawanshahar DH	45929571	12885593	78973549
Punjab	Pathankot	Pathankot DH	48687690	15007320	82368060
Punjab	Patiala	M.K.H. Patiala DH	54558683	18369798	90747568
Punjab	Rupnagar	Rupnagar DH	44854215	12456970	77251459
Punjab	Sangrur	Sangrur DH	48363272	13855634	82870910
Punjab	Tarn Taran	Tarn Taran DH	49778215	14419612	85136817
Rajasthan	Ajmer	A K Hospital Beawar Ajmer	99613871	51401876	147725865
Rajasthan	Alwar	Rajeev Gandhi Govt Genaral Hospital Alwar	109678878	50584201	168773555
Rajasthan	Banswara	District Hospital Banswara	88401761	37052879	139750643
Rajasthan	Baran	District Hospital Baran	82145936	32085237	132206635
Rajasthan	Barmer	District Hospital Barmar	81320588	30774782	131866393
Rajasthan	Bharatpur	RBM Hospital, Bharatpur	98238410	42425414	154051406
Rajasthan	Bhilwara	M G Hospital Bhilwara	99313766	42854036	155773495
Rajasthan	Bundi	Pandit Briz Sundar Sharma General Hospital Bundi	80336897	30689409	129984385
Rajasthan	Chittorgarh	District Hospital Chittaurgarh	103442038	45031148	161852929
Rajasthan	Churu	D B Government Hospital Churu	65847886	24914280	106781492
Rajasthan	Dausa	District Hospital Dausa	72315192	26469686	118160698
Rajasthan	Dholpur	Sadar Hospital DHolpur	88286836	35902905	140670768
Rajasthan	Dungarpur	Shri Hari Dev Joshi Genaral Hospital Dungarpur	74038470	27279247	120797692
Rajasthan	Sri Ganganagar	Govt Hospitls Sriganganagar	74463459	29779662	119147257
Rajasthan	Hanumangarh	DH Hanumangarh Town	66924515	25486543	108362487
Rajasthan	Jaisalmer	Jawahar Hospital Jaisalmer	62327736	20444063	104211408
Rajasthan	Jalore	District Hospital Jalor	70194816	23579778	116809854
Rajasthan	Jhunjhunu	B.D.K. Hospital Jhunjhunun	56935786	19317280	94554292
Rajasthan	Karauli	General Hospital Karauli	82600805	31591773	133609837
Rajasthan	Nagaur	District Hospital Nagaur	66510161	25096464	107923858
Rajasthan	Pali	Govt Bangur Hopital Pali	79828790	30630017	129027563
Rajasthan	Pratapgarh	District Hospital Pratapgarh	66798954	22226232	111371676
Rajasthan	Rajsamand	RK District Hospital Rajsamand	73207217	26211809	120202625
Rajasthan	Sawai Madhopur	General Hospital Sawai Madhopur	84952562	32406467	137498656
Rajasthan	Sikar	S K Hospital, Sikar	73514837	30014980	117014694
Rajasthan	Sirohi	General Hospital Sirohi	73292521	25836860	120748183
Rajasthan	Tonk	District Sahadat Hospital Tonk	71481395	25114968	117847821
Sikkim	East Sikkim	Singtam Hospital	42300142	11541188	73059095
Sikkim	North Sikkim	Mangan Hospital	40454762	9681029	71228496
Sikkim	South Sikkim	Namchi District Hospital	42250721	11419252	73082190

Sikkim	West Sikkim	District Hospital Gyalshing	42336025	11044302	73627747
Tamil Nadu	Ariyalur	Ariyalur	56957773	19980365	93935181
Tamil Nadu	Chennai	Kilpauk Hospital	180499364	139742625	221256102
Tamil Nadu	Coimbatore	Pollachi	76498293	40429822	112466763
Tamil Nadu	Cuddalore	Cuddalore	101933899	48499084	155368715
Tamil Nadu	Dharmapuri	Pennagaram	49092323	15209496	82975149
Tamil Nadu	Dindigul	Dindigul	91897022	42351524	141442519
Tamil Nadu	Erode	Erode	114294861	63387788	165101933
Tamil Nadu	Kancheepuram	Kancheepuram	87674082	38582667	136765497
Tamil Nadu	Kanniyakumari	Padhmanabapuram	43271209	12930172	73612245
Tamil Nadu	Karur	Kulithalai	43480244	11970666	74989823
Tamil Nadu	Krishnagiri	Krishnagiri	74060556	30682342	117438770
Tamil Nadu	Madurai	Usilampatti	68963523	34788829	103038216
Tamil Nadu	Nagapattinam	Nagapattinam	82871726	36709406	129034047
Tamil Nadu	Namakkal	Namakkal	75556628	32219243	118894012
Tamil Nadu	The Nilgiris	Uthagamandalam	65683639	26382379	104984900
Tamil Nadu	Perambalur	Perambalur	77603866	33219274	121988459
Tamil Nadu	Pudukkottai	Aranthangi	50348466	16037339	84659592
Tamil Nadu	Ramanathapuram	Ramanathapuram	87743403	40041625	135445180
Tamil Nadu	Salem	Mettur Dam	78420070	40909552	115830588
Tamil Nadu	Sivaganga	Karaikudi	54142241	18653655	89630827
Tamil Nadu	Thanjavur	Kumbakonam	99353784	53874599	144732968
Tamil Nadu	Theni	Periakulam	61303172	23021026	99585318
Tamil Nadu	Thiruvallur	Thiruvallur	65521023	26215324	104826721
Tamil Nadu	Thiruvarur	Mannargudi	66641983	26334964	106949003
Tamil Nadu	Tiruchirappalli	Manapparai	70625571	35962488	105188653
Tamil Nadu	Tirunelveli	Tenkasi	72121643	37499390	106643896
Tamil Nadu	Tiruppur	Tiruppur	97757478	46343676	149171280
Tamil Nadu	Tiruvannamalai	Cheyyar	72221300	36925685	107416914
Tamil Nadu	Thoothukudi	Kovilpatti	70145594	28937862	111353326
Tamil Nadu	Vellore	Walajapet	78703058	41022347	116283770
Tamil Nadu	Viluppuram	Kallakurichi	62299129	23581582	101016675
Tamil Nadu	Virudhunagar	Virudhunagar	69663648	28193681	111133616
Telangana	Hyderabad	Kingkoti	104713533	91173497	118253569
Telangana	Karimnagar	Karimnagar	81860960	35386389	128335531
Telangana	Khammam	DH Khammam	62184204	22431607	101936800
Telangana	Nalgonda	Nalgonda	79385829	33377457	125394201
Telangana	Sangareddy	DH Sangareddy	60825859	21890189	99761529
Telangana	Vikarabad	Tandur	72481268	28846454	116116082
Tripura	Dhalai	DHalai District Hospital	58592288	18955163	98229413
Tripura	Gomati	District Hospital Gomati District	53215506	16812048	89618964
Tripura	Khowai	Khowai District Hospital	50570582	14735439	86405726
Tripura	North Tripura	District Hospital North Tripura	57758490	17600445	97916536
Tripura	South Tripura	District Hospital South	47401111	13472130	81330093

Tripura	Unakoti	District Hospital Unakoti District	59158265	19180754	99135776
Uttar Pradesh	Agra	DH Male	80693143	38298592	122987693
Uttar Pradesh	Agra	DH Female	87109855	42328432	131791277
Uttar Pradesh	Aligarh	Pt Deen Dayal District Combined Hospital	82612375	38491042	126633709
Uttar Pradesh	Aligarh	Mohan Lal Gautam District Female Hospital	81721165	37931342	125410989
Uttar Pradesh	Aligarh	Malkhan Singh District Hospital	94376347	45879082	142773613
Uttar Pradesh	Ambedkar Nagar	Mahatma Jyotiba Phule District Hospital	55268192	16607845	93928539
Uttar Pradesh	Auraiya	District Combined Hospital Auraiya	55339958	15614073	95065843
Uttar Pradesh	Auraiya	District Combined Hospital Chicholi	59796008	18412573	101179443
Uttar Pradesh	Azamgarh	District Women Hospital	55551180	16720640	94381720
Uttar Pradesh	Azamgarh	District Hospital Azamgarh	66156579	23381070	108932088
Uttar Pradesh	Baghpat	District Combined Hospital	56966123	17284618	96647628
Uttar Pradesh	Bahraich	District Women Hosp	75423334	25357084	125489585
Uttar Pradesh	Bahraich	District Male Hosp	94851712	37558544	152144881
Uttar Pradesh	Ballia	District Male Hospitol Ballia	60683043	20320146	101045939
Uttar Pradesh	Ballia	District Fimale Hospitol Ballia	50790612	14107476	87473747
Uttar Pradesh	Balrampur	District Women Hospital	63348940	18397397	108300482
Uttar Pradesh	Balrampur	District Memeorial Male Hospital	67270264	20860077	113680450
Uttar Pradesh	Balrampur	District Combined Hospital	69587410	22315297	116859522
Uttar Pradesh	Banda	DWH Banda	57802365	16186605	99418125
Uttar Pradesh	Banda	DH Banda	64308198	20272415	108343981
Uttar Pradesh	Barabanki	DWH Barabanki	62605178	19021082	106189273
Uttar Pradesh	Barabanki	DH Barabanki	68398043	22659132	114136953
Uttar Pradesh	Bareilly	District Male Hospital	104815313	51941537	157589088
Uttar Pradesh	Bareilly	District Female Hospital	86010782	40131867	131789696
Uttar Pradesh	Basti	District Female Hospital	61967435	19789263	104145606
Uttar Pradesh	Basti	District Male Hospital	70077446	24882533	115272358
Uttar Pradesh	Basti	Opec Hospital Kaily	77563610	29584013	125543206
Uttar Pradesh	Bijnor	Pandit Deendayal Upadyaya District Combined Hospital [illegible]	59852605	18435132	101270079
Uttar Pradesh	Bijnor	Pandit Deendayal Upadyaya District Combined Hospital [illegible]	57624580	17035882	98213279
Uttar Pradesh	Budaun	District Female Hospital Budaun	73998213	23643987	124352440
Uttar Pradesh	Budaun	District Hospital Badaun	87811968	32319337	143304600
Uttar Pradesh	Bulandshahr	K.M.C Bulandshahr	60589190	17910823	103267558
Uttar Pradesh	Bulandshahr	Joint Hospital Sikandrabad	59697980	17351123	102044838
Uttar Pradesh	Bulandshahr	Ssmj Hospital Khurja	59876222	17463063	102289382
Uttar Pradesh	Bulandshahr	B.B.D.Government Hospital	71016347	24459313	117573382
Uttar Pradesh	Chandauli	Pt K P T DistrictCombined Hospital Chandauli	58154674	17758359	98550990
Uttar Pradesh	Chandauli	Rajkiya Mahila Chikitsalaya Mughalsarai	51916204	13840459	89991950
Uttar Pradesh	Chandauli	Combined Hospital Chakiya Chandauli	58154674	17758359	98550990
Uttar Pradesh	Chitrakoot	District Combined Hospital	66417938	21051987	111783890
Uttar Pradesh	Deoria	District Hospital Female	61397283	20911546	101883020
Uttar Pradesh	Deoria	District Hospital Male	64873002	23094376	106651628
Uttar Pradesh	Etah	Distt Male Hospital	69644007	22337856	116950159
Uttar Pradesh	Etah	District Women Hospital	62336085	17748316	106923855

Uttar Pradesh	Etawah	District Women Hospital F	56697030	16011851	97382209
Uttar Pradesh	Etawah	District Male Hospital	73362657	26478241	120247073
Uttar Pradesh	Faizabad	DistrictFemale Hospital	61779472	19489421	104069522
Uttar Pradesh	Faizabad	Sri Ram Ayodya	59194963	17866291	100523634
Uttar Pradesh	Faizabad	Distt Male Hospital	70513330	24974481	116052178
Uttar Pradesh	Farrukhabad	Dr Ram Manohar Lohiya Female	66185186	20243551	112126821
Uttar Pradesh	Farrukhabad	Dr Ram Manohar Lohiya Male	78216521	27799501	128633541
Uttar Pradesh	Farrukhabad	Civil Hospital Linziganj	63065951	18284601	107847301
Uttar Pradesh	Fatehpur	District Hospital Female	59224941	17612422	100837461
Uttar Pradesh	Fatehpur	District Hospital Male	65819895	21754202	109885589
Uttar Pradesh	Firozabad	District Women Hospital	61516283	17973637	105058930
Uttar Pradesh	Firozabad	Rnm District Joint Hospital	64635518	19932587	109338450
Uttar Pradesh	Gautam Buddha Nagar	Combined Distt Hospital Noida	58339278	18588498	98090057
Uttar Pradesh	Ghaziabad	District Women Hospital	51680549	14523536	88837563
Uttar Pradesh	Ghaziabad	District Combined Hospital Sanjay Nagar	54532421	16314576	92750267
Uttar Pradesh	Ghaziabad	District Mmg Male Hospital	60414407	20008596	100820219
Uttar Pradesh	Ghazipur	District Woman Hospital	55013909	16097540	93930279
Uttar Pradesh	Ghazipur	District Hospital	61252379	20015440	102489319
Uttar Pradesh	Gonda	DWH	70353616	23315913	117391318
Uttar Pradesh	Gonda	DH	73918456	25554713	122282198
Uttar Pradesh	Gorakhpur	Neta Ji Subhash Chandra Bose District Hospital	89958824	45610985	134206663
Uttar Pradesh	Gorakhpur	District Women Hospital	75342980	36431905	114154055
Uttar Pradesh	Hamirpur	District Women Hospital	69865170	31978845	107651494
Uttar Pradesh	Hamirpur	District Men Hospital	73251768	34105705	112297830
Uttar Pradesh	Hardoi	District Women Hospital	65982870	20142463	111823276
Uttar Pradesh	Hardoi	District Male Hospita	76677390	26858863	126495916
Uttar Pradesh	Hathras	Bagala Joint District Hospital, Hathras	62782148	18989383	106574913
Uttar Pradesh	Hathras	District Female Hospital Hathras	59217308	16750583	101684033
Uttar Pradesh	Jalaun	District Women Hospital	53854064	15226290	92481838
Uttar Pradesh	Jalaun	District Hospital	57775388	17688970	97861806
Uttar Pradesh	Jaunpur	District Femail Hospital	59626214	18344895	100907534
Uttar Pradesh	Jaunpur	District Male Hospital	63636659	20863545	106409774
Uttar Pradesh	Jhansi	District Women Hospital	72398985	33336399	111361572
Uttar Pradesh	Jhansi	District Hospital	83539110	40332649	126645572
Uttar Pradesh	Amroha	Amroha	63814444	20014269	107614620
Uttar Pradesh	Kannauj	Combined District Hospital Kannauj	64154030	20149623	108158438
Uttar Pradesh	Kanpur Dehat	District Combined Hospital	75028283	35468122	114488445
Uttar Pradesh	Kanpur Nagar	UHM Male Hospital	101324068	52553832	149994303
Uttar Pradesh	Kanpur Nagar	Kpm Hospital Kanpur Nagar	69686113	32684482	106587743
Uttar Pradesh	Kanpur Nagar	Manyawar Kashiram Hospital	72537985	34475522	110500447
Uttar Pradesh	Kanpur Nagar	Distric Women Hospital	80113270	39232972	120893567
Uttar Pradesh	Kashi Ram Nagar	WH	67027790	19863738	114191842
Uttar Pradesh	Kaushambi	District Combined Hospital	66474536	21074546	111874526
Uttar Pradesh	Khushinagar	District Combined Hospital Kushinagar	55381387	16652963	94109811

Uttar Pradesh	Lakhimpur Kheri	DFH	65909374	20031379	111787370
Uttar Pradesh	Lakhimpur Kheri	DH	75445321	26020169	124870474
Uttar Pradesh	Lalitpur	District Female Hospital	60985374	18068737	103902012
Uttar Pradesh	Lalitpur	District Male Hospital	68115054	22546337	113683772
Uttar Pradesh	Lucknow	Shyama Prasad Mukherjee	98889909	50622574	147057245
Uttar Pradesh	Lucknow	Balrampur Hospital Lucknow	123843789	66294174	181293405
Uttar Pradesh	Lucknow	Awanti Bai Mahila Hospitals	94433859	47824074	140943645
Uttar Pradesh	Lucknow	Ram Manohar Lohiya	106910799	55659874	158061725
Uttar Pradesh	Lucknow	Rani Laxmi Bai Combined Hospital	75183723	35734554	114532893
Uttar Pradesh	Lucknow	Lokbandhu Raj Narain	74292513	35174854	113310173
Uttar Pradesh	Lucknow	Jhalkari Bai Mahila Hospitals	72688335	34167394	111109277
Uttar Pradesh	Lucknow	RSM Combined Hospital	74292513	35174854	113310173
Uttar Pradesh	Lucknow	Bhau Rao Devras Hospital Mahanagar	74292513	35174854	113310173
Uttar Pradesh	Maharajganj	District Combined Hospital	58211272	17780918	98641626
Uttar Pradesh	Mahoba	DWH Mahoba	56387423	15622628	97152218
Uttar Pradesh	Mahoba	DH Mahoba	62625893	19540528	105711258
Uttar Pradesh	Mainpuri	District Female Hospital	59726687	16953614	102499759
Uttar Pradesh	Mainpuri	District Male Hospital	65965157	20871514	111058799
Uttar Pradesh	Mathura	District Women Hospital	75003752	34517757	115389746
Uttar Pradesh	Mathura	District Combined Hospital	79103318	37092377	121014258
Uttar Pradesh	Mathura	District Male Hospital	76786172	35637157	117835186
Uttar Pradesh	Maunathbhanjan	District Women Hospital	48010963	12283881	83738045
Uttar Pradesh	Maunathbhanjan	District Hospital	51575803	14522681	88628925
Uttar Pradesh	Meerut	District Women Hospital	76963599	36566674	117260523
Uttar Pradesh	Meerut	P.L. Sharma Hospital	88905813	44066654	133644971
Uttar Pradesh	Mirzapur	DistrictWoman Hospital Mzp	62235613	19139597	105331629
Uttar Pradesh	Mirzapur	District Hospital Mirzapur	68206720	22889587	113523853
Uttar Pradesh	Moradabad	Male District Hospital	89630132	43067163	136093101
Uttar Pradesh	Moradabad	Female District Hospital	79915943	36966433	122765453
Uttar Pradesh	Muzaffarnagar	Female District Hospital Muzaffar Nagar	61692895	19720731	103665060
Uttar Pradesh	Muzaffarnagar	Swami Kalyan Dev District Hospital Muzaffar Nagar	65970703	22407291	109534116
Uttar Pradesh	Pilibhit	District Male Hospital	65525913	21309864	109741963
Uttar Pradesh	Pilibhit	District Women Hospital	60178653	17951664	102405643
Uttar Pradesh	Pratapgarh	District Women Hospital	58956306	17300872	100611740
Uttar Pradesh	Pratapgarh	District Male Hospital	69472584	23905332	115039836
Uttar Pradesh	Prayagraj	Moti Lal Nehru District Hospital	86754186	41286975	132121396
Uttar Pradesh	Prayagraj	District Women Hospital	87467154	41734735	133099572
Uttar Pradesh	Prayagraj	Tej Bahadur Sapru Hospital	90586389	43693685	137379092
Uttar Pradesh	Rae Bareli	District Female Hospital	62629709	19971447	105287972
Uttar Pradesh	Rae Bareli	District Hospital	73948076	27079637	120816516
Uttar Pradesh	Rampur	District Male Hospital	67987506	22699973	113275038
Uttar Pradesh	Rampur	District Woman Hospital	59253648	17214913	101292382
Uttar Pradesh	Saharanpur	District Women Hospital	64158305	21541760	106774850
Uttar Pradesh	Saharanpur	SBD District Hospital	77704697	30049200	125360194

Uttar Pradesh	Sambhal	District Combined Hospital	68498515	21267852	115729179
Uttar Pradesh	Sant Kabir Nagar	District Combined Hospital Sant Kabir Nagar	57362306	17442531	97282082
Uttar Pradesh	Sant Ravidas Nagar	Maharaja Chet Singh District Hospital	62456099	19472850	105439349
Uttar Pradesh	Sant Ravidas Nagar	Maharaja Balavant Singh Distric Hospital	58000049	16674350	99325749
Uttar Pradesh	Shahjahanpur	District Women Hospital	71998309	23439830	120556787
Uttar Pradesh	Shahjahanpur	District Male Hospital	80553925	28812950	132294899
Uttar Pradesh	Shravasti	Combined District Hospital	72643685	23533488	121753883
Uttar Pradesh	Siddharth Nagar	District Combined Hospital	63418260	19856355	106980166
Uttar Pradesh	Sitapur	District Women Hospital	73112550	24620063	121605036
Uttar Pradesh	Sitapur	District Hospital Male Sitapur	78103326	27754383	128452268
Uttar Pradesh	Sonbhadra	District Combined Hospital Robertsganj	60475180	18683282	102267078
Uttar Pradesh	Sultanpur	District Women Hospital	56663692	16796017	96531366
Uttar Pradesh	Sultanpur	District Hospital	68071180	23960177	112182182
Uttar Pradesh	Unnao	Uma Shankar Female Hospital	57872501	16827986	98917015
Uttar Pradesh	Unnao	Uma Shanker Male Hospital	62328551	19626486	105030615
Uttar Pradesh	Varanasi	District Women Hospital Varanasi	81761780	39787809	123635751
Uttar Pradesh	Varanasi	Pt. Deen Dayal Upadhyay Govt Hospital	76860125	36709459	116910791
Uttar Pradesh	Varanasi	Lbs Ramnagar Hospital	79355513	38276619	120334407
Uttar Pradesh	Varanasi	S.S.P.G. Div. Dist. Hospital	91208606	45720629	136596583
Uttarakhand	Almora	Distt Female Hosptial	38471298	8604181	68338416
Uttarakhand	Almora	Distt Hosptial Almora	40521081	9891491	71150672
Uttarakhand	Bageshwar	Shyam Lal Shah DH	39839364	9333502	70345227
Uttarakhand	Chamoli	District Hospital	40983584	10259866	71707302
Uttarakhand	Champawat	DH Champawat	41310905	9920039	72701771
Uttarakhand	Pauri Garhwal	DH Pauri	49404018	14924783	83883252
Uttarakhand	Pauri Garhwal	DH Female Pauri	40491918	9327783	71656052
Uttarakhand	Haridwar	Cr Women Govt Hospital	46120437	11693922	80546951
Uttarakhand	Haridwar	Hmg Hospital Hardwar	48972309	13484962	84459655
Uttarakhand	Nainital	B.D.Pandey Male Hospital	42632094	10814703	74449485
Uttarakhand	Nainital	B.D.Pandey Female Hospital	41740884	10255003	73226765
Uttarakhand	Pithoragarh	H G Pant District Female Hospital	40033232	9390327	70676136
Uttarakhand	Pithoragarh	B D Pandey District Male Hospital	46806428	13644047	79968808
Uttarakhand	Rudraprayag	District Hospital Rudraprayag	40454762	9681029	71228496
Uttarakhand	Tehri Garhwal	DH Bauradi	44356644	11767904	76945384
Uttarakhand	Udham Singh Nagar	J.L.N. District Hospital	51440263	15593271	87287254
Uttarakhand	Uttarkashi	District Hospital	48906447	14235717	83577177
Uttarakhand	Uttarkashi	District Female Hospital	43737429	10989457	76485401
West Bengal	Alipurduar	Alipurduar District Hospital	63677731	23681229	103674233
West Bengal	Birbhum	Rampuhat DH & SSH	68335640	25619600	111051680
West Bengal	Dakshin Dinajpur	Balurghat DH & SSH	79131547	32867153	125395941
West Bengal	Darjeeling	Siliguri DH	71244567	28394416	114094718
West Bengal	Darjeeling	Darjeeling DH	69283905	27163076	111404734
West Bengal	Howrah	Howrah District Hospital	90868542	41757558	139979526
West Bengal	Hooghly	Imambara District Hospital	91642740	41841220	141444259

West Bengal	Jalpaiguri	Jalpaiguri DH & SSH	101609481	47347398	155871565
West Bengal	Jhargram	Jhargram DH & SSH	80277039	33937157	126616921
West Bengal	Cooch Behar	Mjn District Hospital	85313419	36762494	133864345
West Bengal	Nadia	District Hospital Nadia	113756556	55235746	172277367
West Bengal	North 24 Parganas	Barasat DH	88848738	40216379	137481096
West Bengal	North 24 Parganas	Basirhat DH & SSH	84303567	37361909	131245224
West Bengal	Paschim Burdwan	Asansol DH & SSH	93550740	49087430	137914051
West Bengal	Purba Medinipur	Tamluk District Hospital	73323514	30245434	116401593
West Bengal	Purba Medinipur	Nandigram DH & SSH	42576769	10935784	74217753
West Bengal	Puruliya	D.M.Sadar DH & SSH	112924608	60592394	165156822
West Bengal	South 24 Parganas	M. R. Bangur DH & SSH	91925728	41954016	141897441
West Bengal	South 24 Parganas	Diamond Harbour DH & SSH	74279770	30871956	117687585
West Bengal	Uttar Dinajpur	Raiganj DH & SSH	82722549	34421164	131023933
Bihar	Darbhanga		54998741	17113871	92883611
Bihar	Patna		86233097	44309987	128056207
Madhya Pradesh	Niwari		72429660	26658444	118200875
Jharkhand	Dhanbad		72919000	35649767	110088234
Uttar Pradesh	Amethi		62051467	19270674	104832259
Uttar Pradesh	Hapur		60693122	18729255	102656988
Uttar Pradesh	Shamli		58429214	17826891	99031536
Rajasthan	Bikaner		90331391	46740967	133821814
Rajasthan	Jaipur		90161598	46673290	133549905
Rajasthan	Jhalawar		80373237	31153740	129592735
Rajasthan	Jodhpur		92368908	47553095	137084721
Rajasthan	Kota		78279122	30319053	126239192
Rajasthan	Udaipur		84335076	32732877	135937276
Chhattisgarh	Bastar		62044747	20331268	103758227
Chhattisgarh	Korea		62157943	20376386	103939499
Chhattisgarh	Rajnandgaon		60686402	19789849	101582956
Chhattisgarh	Surajpur		61931552	20286149	103576954
Chhattisgarh	Surguja		59837437	19451463	100223411
Assam	Biswanath		59378751	19514007	99243495
Assam	Charaideo		65830889	22085744	109576033
Assam	Dibrugarh		62038843	20574285	103503401
Assam	Hojai		71434061	24319095	118549027
Assam	Jorhat		65208314	21837594	108579034
Assam	Majuli		64415946	21521767	107310126
Assam	South Salmara Mancachar		57567625	18792116	96343133
Andhra Pradesh	YSR		77707698	31297696	124117701
West Bengal	Bankura		84853461	36681398	133025524
West Bengal	Kalimpong		80552036	34966906	126137165
West Bengal	Kolkata		125725832	104824189	146627474
West Bengal	Maldah		89381277	38486126	140276428
West Bengal	Murshidabad		86551392	37358171	135744613

West Bengal	Paschim Medinipur		82872542	35891830	129853254
West Bengal	Purba Burdwan		99914672	53525631	146203713
Tripura	Sepahijala		49006562	14623230	83389894
Tripura	West Tripura		49063159	14645789	83480530
Arunachal Pradesh	Anjaw		44953057	12700843	77205271
Arunachal Pradesh	Changlang		47443356	13693443	81193268
Arunachal Pradesh	East Kameng		44160689	12385015	75936363
Arunachal Pradesh	Kamle		43877701	12272220	75483181
Arunachal Pradesh	Kra Daddi		42915540	11888715	73942364
Arunachal Pradesh	Kurung Kumey		44330482	12452693	76208272
Arunachal Pradesh	Longding		50160045	14776280	85543811
Arunachal Pradesh	Lower Siang		46367999	13264820	79471179
Arunachal Pradesh	Lower Subansiri		43141931	11978952	74304910
Arunachal Pradesh	Namsai		45462436	12903875	78020998
Arunachal Pradesh	Pakke Kessang		43707908	12204543	75211273
Arunachal Pradesh	Shi Yomi		42519356	11730801	73307910
Arunachal Pradesh	Tirap		49594068	14550689	84637448
Arunachal Pradesh	Upper Siang		40368643	10873556	69863731
Arunachal Pradesh	Upper Subansiri		42406160	11685683	73126638
Arunachal Pradesh	West Kameng		43481517	12114306	74848727
Meghalaya	East Jaintia Hills		70079076	23247380	116910772
Meghalaya	North Garo Hills		50496272	15441931	85550612
Meghalaya	South Garo Hills		49251122	14945631	83556614
Meghalaya	South West Garo Hills		51684824	15915673	87453975
Meghalaya	South West Khasi Hills		52533789	16254059	88813519
Karnataka	Bengaluru Rural		134486667	108806884	160166450
Karnataka	Vijaypura		86470481	44731766	128109197
Telangana	Adilabad		72481268	28846454	116116082
Telangana	Bhadradri Kothagudem		75084762	29884172	120285351
Telangana	Jagitial		70839934	28192240	113487629
Telangana	Jangoan		69990969	27853853	112128084
Telangana	Jayashankar Bhupalpally		72537865	28869013	116206718
Telangana	Jogulamba Gadwal		76952486	30628622	123276349
Telangana	Kamareddy		71066325	28282476	113850174
Telangana	Kumuram Bheem Asifabad		72424670	28823894	116025445
Telangana	Mahabubnagar		74179199	29523227	118835171
Telangana	Mahuababad		71236118	28350153	114122083
Telangana	Mancherial		71462509	28440390	114484628
Telangana	Medak		71236118	28350153	114122083
Telangana	Medchal Malkajgiri		68066647	27086844	109046450
Telangana	Nagarkurnool		75311153	29974409	120647897
Telangana	Nirmal		71858693	28598303	115119082
Telangana	Nizamabad		70839934	28192240	113487629
Telangana	Peddapalli		70160762	27921530	112399993

Telangana	Rajanna Sircilla		70500348	28056885	112943811
Telangana	Rangareddy		70047566	27876412	112218721
Telangana	Siddipet		70273957	27966649	112581266
Telangana	Suryapet		71236118	28350153	114122083
Telangana	Wanaparthy		75877130	30200000	121554260
Telangana	Warangal Rural		82893833	42979000	122708665
Telangana	Warangal Urban		81535488	42437582	120533394
Telangana	Yadadri Bhuvanagiri		70330555	27989208	112671902
Gujarat	Ahmedabad		134045694	108140377	159951011
Gujarat	Arvalli		62736185	21077159	104395212
Gujarat	Bhavnagar		77172734	38347178	115898289
Gujarat	Gandhinagar		56850025	18731013	94969036
Gujarat	Gir Somnath		53680553	17467703	89893404
Gujarat	Jamnagar		73154297	36745482	109463112
Gujarat	Junagadh		52152416	16858608	87446224
Gujarat	Kachchh		59849703	19926645	99772760
Gujarat	Patan		62283404	20896686	103670121
Gujarat	Sabar Kantha		61943818	20761332	103126303
Gujarat	Surat		72871309	36632687	109009930
Gujarat	Valsad		53397565	17354908	89440222
Manipur	Imphal East		39195260	9383483	69007036
Manipur	Imphal West		38742478	9203011	68281946
Manipur	Jiribam		48477283	13083176	83871389
Manipur	Kakching		39534846	9518838	69550854
Manipur	Kamjong		40610202	9947461	71272944
Manipur	Kangpokpi		39591444	9541397	69641490
Manipur	Noney		44402248	11458921	77345576
Manipur	Pherzawl		44289053	11413802	77164303
Manipur	Tengnoupal		40666800	9970020	71363580
Jammu and Kashmir	Kishtwar		52777893	15615244	89940541
Jammu and Kashmir	PoJK		55381387	16652963	94109811
Jammu and Kashmir	Poonch		50966766	14893353	87040180
Jammu and Kashmir	Rajouri		49212238	14194021	84230454
Haryana	Charkhi Dadri		55721430	17749534	93693327
Haryana	Gurugram		79129579	39147599	119011559
Haryana	Karnal		78789993	39012244	118467741
Haryana	Yamunanagar		55834626	17794652	93874599
Uttarakhand	Dehradun		83629603	43272269	123886937
Maharashtra	Akola		60616366	21888478	99344254
Maharashtra	Chandrapur		63559446	23061551	104057342
Maharashtra	Dhule		61521929	22249423	100794435
Maharashtra	Gondia		64238619	23332260	105144977
Maharashtra	Kolhapur		81679119	43190048	120068191
Maharashtra	Latur		58352458	20986114	95718802

Maharashtra	Mumbai City		115830856	98324239	133337473
Maharashtra	Mumbai Suburban		115661063	98256562	133065564
Maharashtra	Nagpur		87169096	45378281	128859912
Maharashtra	Nanded		86999303	45310603	128588003
Maharashtra	Palghar		59767400	21550091	97984710
Maharashtra	Sangli		82188499	43393080	120883917
Maharashtra	Solapur		83433648	43889380	122877916
Maharashtra	Yavatmal		61974711	22429896	101519525
Nagaland	Kiphire		46878651	13611492	80145810
Nagaland	Kohima		47218237	13746847	80689628
Nagaland	Longleng		50557502	15077834	86037170
Nagaland	Mon		51576260	15483897	87668623
Nagaland	Peren		51293272	15371102	87215441
Nagaland	Phek		46142881	13318224	78967538
Nagaland	Tuensang		48180398	14130351	82230445
Nagaland	Zunheboto		47274835	13769406	80780264
Himachal Pradesh	Kinnaur		61757584	21566346	101948822
Delhi	New Delhi		107992840	93012222	122973458
Delhi	South East		108445622	93192695	123698548

Supplementary Material 3		
**Cost of delivering inpatient care under health benefit packages specified in AB PM-JAY**		
**Name of Speciality**	**Health Benefit Package Name**	**Package Cost in ₹ (Inclusive of full recurrent cost and 20% capital cost)**
Pediatrics	Febrile Seizures	4082.1
Pediatrics	Flury of seizures	5758.5
Pediatrics	Neurocysticercosis	17090.8
Pediatrics	Epilepsy	4771.4
Pediatrics	Epileptic encephalopathy	17672.0
Pediatrics	Infectious - uncomplicated	17672.0
Pediatrics	Immune-mediated - uncomplicated	49994.2
Pediatrics	Acute encephalitis syndrome	17672.0
Pediatrics	Acute meningo encephalitis	17672.0
Pediatrics	Aseptic meningitis	7006.3
Pediatrics	Febrile encephalopathy	17672.0
Pediatrics	Hypertensive encehalopathy	12655.4
Pediatrics	Metabolic encephalopathy	28329.4
Pediatrics	Hepatic encephalopathy	16430.9
Pediatrics	Brain Abscess	7339.7
Pediatrics	Chronic meningitis	17672.0
Pediatrics	Partially treated pyogenic meningitis	17666.6
Pediatrics	Neuro tuberculosis	17404.4
Pediatrics	Complicated bacterial meningitis	17672.0
Pediatrics	Acute meningitis	17672.0
Pediatrics	Optic neuritis	21116.9
Pediatrics	Intracranial Hemorrhage	18762.7
Pediatrics	Intracranial space occupying lesion	17301.6
Pediatrics	Intracranial ring enhancing lesion with complication (tuberc	17698.4
Pediatrics	Cerebral herniation	17672.0
Pediatrics	Acute neuroregression / Acute worsening in neuro metabol	17672.0
Pediatrics	Acute demyelinating myelopathy	21725.8
Pediatrics	Juvenile Myasthenia	5466.1
Pediatrics	Acute Ataxia	14924.5
Pediatrics	Acute ischemic stroke	17672.0
Pediatrics	Wheezing	4618.4
Pediatrics	Chronic Cough	9230.9
Pediatrics	Acute urticaria	1482.0
Pediatrics	Anaphylaxis acute asthma	9052.1
Pediatrics	Acute Abdomen	5993.1
Pediatrics	Unexplained hepatosplenomegaly	5003.4
Pediatrics	Infantile Cholestasis	10186.3
Pediatrics	Acute glomerulonephritis	6858.8
Pediatrics	Nephrotic syndrome with peritonitis	5063.2
Pediatrics	Haemolytic uremic syndrome	15930.6
Pediatrics	Global Developmental delay	6529.1
Pediatrics	Intellectual Disability of unknown etiology	6522.0

Pediatrics	Rickets - requiring admission for work up	7249.0
Pediatrics	Acute Severe Malnutrition	5199.0
Pediatrics	Developmental and behavioral disorders	6529.1
Pediatrics	Short stature	5404.0
Pediatrics	Dysmorphic children	6529.1
Pediatrics	Floppy infant	9006.8
Pediatrics	Inborn errors of metabolism	28141.3
Pediatrics	Wilson's disease	15285.5
Pediatrics	Rheumatoid arthritis	11157.5
Pediatrics	Rheumatic fever	10883.0
Pediatrics	Cyanotic spells	6902.9
Pediatrics	Cyanotic spells with CHD	6902.9
Pediatrics	Cyanotic Spells with Chest Infection	7492.8
Pediatrics	Cyanotic Spells with sepsis	7492.8
Pediatrics	Immune haemolytic anemia	25376.7
Pediatrics	Idiopathic Thrombocytopenic Purpura	25462.6
Pediatrics	Kawasaki Disease	47833.1
Pediatrics	Steven Johnson syndrome	18633.5
Pediatrics	Ketogenic diet initiation in refractory epilepsy	7309.2
Medicine	Acute febrile illness- Acute febrile illness	6962.6
Medicine	Severe sepsis - Severe sepsis	11276.8
Medicine	Severe sepsis - Septic shock	11294.8
Medicine	Malaria - Malaria	7127.2
Medicine	Malaria - Complicated malaria	6906.5
Medicine	Dengue fever - Dengue fever	6870.4
Medicine	Dengue fever - Dengue hemorrhagic fever	6870.4
Medicine	Dengue fever - Dengue shock syndrome	6870.4
Medicine	Chikungunya fever - Chikungunya fever	6960.7
Medicine	Enteric fever - Enteric fever	6931.3
Medicine	HIV with complications - HIV with complications	11622.0
Medicine	Leptospirosis- Leptospirosis	7104.9
Medicine	Acute gastroenteritis with dehydration - Acute gastroenteri	6752.6
Medicine	Acute gastroenteritis with dehydration - Acute gastroenteri	6752.6
Medicine	Diarrohea - Chronic diarrohea	6663.9
Medicine	Diarrohea - Persistent diarrohea	6591.7
Medicine	Dysentery - Dysentery	6659.6
Medicine	Acute viral hepatitis- Acute viral hepatitis	18881.0
Medicine	Chronic hepatitis- Chronic Hepatitis	18771.5
Medicine	Liver abscess - Liver abscess	18519.2
Medicine	Visceral leishmaniasis - Visceral leishmaniasis	12066.6
Medicine	Pneumonia - Pneumonia	10549.8
Medicine	Severe pneumonia - Severe pneumonia	10544.3
Medicine	Empyema - Empyema	5631.6
Medicine	Lung abscess - Lung abscess	8487.8
Medicine	Pericardial / Pleural tuberculosis - Pericardial tuberculosis	8956.7
Medicine	Pericardial / Pleural tuberculosis - Pleural tuberculosis	8992.2
Medicine	Urinary Tract Infection - Urinary Tract Infection	3177.8
Medicine	Viral encephalitis - Viral encephalitis	7114.2
Medicine	Septic Arthritis - Septic Arthritis	6085.8

Medicine	Recurrent vomiting with dehydration- Recurrent vomiting w	6681.5
Medicine	Pyrexia of unknown origin- Pyrexia of unknown origin	5781.1
Medicine	Bronchiectasis - Bronchiectasis	16356.4
Medicine	Acute Bronchitis- Acute bronchitis	16283.3
Medicine	Acute excaberation of COPD - Acute excaberation of COPD	18585.2
Medicine	Acute Exacerbation of Interstitial lung Disease- Acute excab	14413.4
Medicine	Endocarditis - Bacterial Endocarditis	16348.3
Medicine	Endocarditis - Fungal Endocarditis	17114.9
Medicine	Vasculitis - Vasculitis	8795.2
Medicine	Pancreatitis - Acute pancreatitis	20396.8
Medicine	Pancreatitis - Chronic pancreatitis	14774.6
Medicine	Acute transverse myelitis- Acute transverse myelitis	35625.7
Medicine	Congestive heart failure- Congestive heart failure	22222.5
Medicine	Asthma- Acute asthmatic attack	13469.6
Medicine	Asthma - Status asthmaticus	14872.3
Medicine	Respiratory failure (type 1)	10560.4
Medicine	Respiratory failure (type 2)	11913.8
Medicine	Respiratory failure- Due to any cause (pneumonia, asthma,	8953.0
Medicine	Upper GI bleeding (conservative)	16373.1
Medicine	Upper GI bleeding (endoscopic)	16373.1
Medicine	Lower GI hemorrhage - Lower GI hemorrhage	9052.0
Medicine	Renal colic - Renal colic	10949.0
Medicine	AKI / Renal failure - AKI / Renal failure	19650.1
Medicine	Seizures - Seizures	7080.0
Medicine	Status epilepticus - Status epilepticus	16309.7
Medicine	Cerebrovascular accident - Cerebrovascular accident	19431.8
Medicine	Cerebral sino-venous thrombosis / Stroke - Cerebral sino-ve	19414.1
Medicine	Cerebral sino-venous thrombosis / Stroke - Acute stroke	19542.0
Medicine	Cerebral sino-venous thrombosis / Stroke - Acute ischemic s	19367.7
Medicine	Cerebral sino-venous thrombosis / Stroke -Acute heamorrh	19367.7
Medicine	Immune mediated CNS disorders- Immune mediated CNS d	10542.9
Medicine	Hydrocephalus- Hydrocephalus	3441.8
Medicine	Pneumothroax - Pneumothroax	10035.8
Medicine	Neuromuscular disorders - Neuromuscular disorders	10049.5
Medicine	Diabetic Foot - Diabetic Foot	12782.1
Medicine	Diabetic ketoacidosis - Diabetic ketoacidosis	12120.3
Medicine	Hyperosmolar Non-Ketotic coma- Hyperosmolar Non-Ketot	6011.4
Medicine	Accelerated hypertension - Accelerated hypertension	26451.8
Medicine	Hypertensive emergencies - Hypertensive emergencies	26305.8
Medicine	Severe anemia - Severe anemia	7930.4
Medicine	Sickle cell Anemia - Sickle cell Anemia	7274.0
Medicine	Heat stroke - Heat stroke	3503.3
Medicine	Systematic lupus erythematosus - Systematic lupus erythen	8873.4
Medicine	Guillian Barre Syndrome - Guillian Barre Syndrome	38075.5
Medicine	Snake bite- Snake bite	14763.8
Medicine	Poisoning (Organophosphorus)	18870.3
Medicine	Poisoning - Other poisonings	11309.9
Medicine	Haemodialysis / Peritoneal Dialysis (only for ARF) - Haemod	21711.2
Medicine	Haemodialysis / Peritoneal Dialysis (only for ARF) - Peritone	20666.7

Medicine	Plasmapheresis - Plasmapheresis	14469.0
Medicine	Blood transfusion - Whole Blood transfusion	1373.3
Medicine	Blood transfusion- Blood component including platelet tran	1843.3
Medicine	High end radiological diagnostic (CT, MRI, Imaging including	2778.1
Medicine	High end histopathology (Biopsies) and advanced serology -	2778.1
OBG	Closure of Burst Abdomen	12528.3
OBG	Abdominal Myomectomy	9321.4
OBG	Caesarean Delivery	13904.5
OBG	Caesarean hysterectomy	12350.8
OBG	D&C (Dilatation & Curretage)	4831.1
OBG	Dilation and Evacuation (D&E)	5248.4
OBG	Electro Cauterisation / Cryo Surgery	7790.2
OBG	Excision of Vaginal Septum (vaginal route)	13281.3
OBG	Hysteroscopic IUCD removal	4214.6
OBG	Hysteroscopic polypectomy	7244.8
OBG	Laparoscopic cystectomy	10034.2
OBG	Laparoscopic tubal surgeries	12783.3
OBG	Laparotomy for benign disorders – Ectopic	13765.4
OBG	Laparotomy for broad ligament haematoma	13992.8
OBG	Manual removal of placenta	10394.7
OBG	McDonald's stitch	9444.7
OBG	High Risk Delivery (eclampsia/ severe anaemia)	22197.5
OBG	MTP > 12 weeks	8631.3
OBG	MTP 8 to 12 weeks	8700.8
OBG	MTP upto 8 weeks	7468.9
OBG	Non descent vaginal hysterectomy	13390.0
OBG	High Risk Delivery (other maternal and fetal conditions as p	14179.4
OBG	Pre-mature delivery	14840.9
OBG	Procedure on Fallopian Tube for establishing Tubal Patency	10661.4
OBG	Secondary suturing of episiotomy	8022.8
OBG	Shirodkar's stitch	6239.8
OBG	Surgeries for Prolapse - Sling Surgeries	13667.6
OBG	Trans-obturator tape	9723.0
OBG	Vaginal hysterectomy with anterior and posterior colpoperi	12254.0
OBG	Vaginoplasty (McIndoe procedure)	10072.8
OBG	Vulvo vaginal cyst enucleation	6692.9
OBG	Hymenectomy for imperforate hymen	6822.3
OBG	Laparoscopic hysterectomy (TLH)	21151.8
OBG	Diagnostic / Staging laparoscopy	10720.4
OBG	Abdominal Hysterectomy + Salpingo-oophorectomy	14660.4
OBG	Laparotomy and proceed for Ovarian Cancers. Omentomy v	19663.8
OBG	Vaginal repair for vesico-vaginal fistula	21771.3
OBG	Cervix Cancer screening (PAP + Colposcopy)	2195.3
ENT	Adenoidectomy	13411.8
ENT	Biopsy	4836.3
ENT	Branchial sinus excision	10774.0
ENT	Endoscopic DCR	13562.7
ENT	Fracture - setting nasal bone	13814.8
ENT	Functional Endoscopic Sinus (FESS)	12401.5

ENT	Functional septo rhinoplasty	15927.7
ENT	Inferior turbinate reduction under GA	12685.7
ENT	Open sinus surgery	26298.5
ENT	Removal of Submandibular Lymph node biopsy	6134.7
ENT	Removal of Submandibular Salivary gland	7765.0
ENT	Septoplasty	15039.9
ENT	Stapedectomy	16056.5
ENT	Thyroglossal cyst excision	13549.3
ENT	Tonsillectomy - U/L	13614.1
ENT	Tonsillectomy - B/L	16043.0
ENT	Tympanoplasty	17662.7
ENT	Tracheostomy	10699.1
ENT	Deep neck abscess drainage	5234.0
ENT	Post traumatic neck exploration	4406.0
ENT	Uvulopalatopharyngoplasty (UPPP)	24861.2
ENT	Excision of tumour of paranasal sinus	9609.1
ENT	Mastoidectomy – Simple	13000.6
ENT	Mastoidectomy – Radical	13032.3
ENT	Myringotomy with or without Grommet – B/L	10371.5
ENT	Myringotomy with or without Grommet – U/L	8299.0
ENT	Pinna surgery for tumour	8032.1
ENT	Superficial Parotidectomy	17473.6
ENT	Total Parotidectomy	17389.8
General Surgey	Foreign Body Removal	8341.4
General Surgey	Hemi thyroidectomy	18910.8
General Surgey	Total thyroidectomy	16846.9
General Surgey	Bleeding Ulcer - Partial Gastrectomy without Vagotomy	37298.8
General Surgey	Debridement of Ulcer	8436.3
General Surgey	Excision Filarial Scrotum	7857.5
General Surgey	Excision Mammary Fistula	9652.6
General Surgey	Excision of cyst / Sebaceous Cysts over scrotum - Single Cys	8341.7
General Surgey	Exicision of Sinus and Curettage	6625.8
General Surgey	Groin Hernia Repair(Femoral-Open)	15186.5
General Surgey	Haemorroidectomy without Stapler	11579.7
General Surgey	Lipoma Excision (Other cutaneous swellings Excision)	7940.7
General Surgey	Lymphatics Excision of Subcutaneous Tissues In Lymphoede	12814.2
General Surgey	Management of Pilonidal Sinus	9212.4
General Surgey	Management of Varicose Veins	18764.3
General Surgey	Microlaryngoscopic Surgery	11952.4
General Surgey	Perineal Procedure for Rectal Prolapse	12686.6
General Surgey	Procedure for Fissure in Ano	16612.1
General Surgey	Abdominal Hydatid Cyst (Single Organ)	19365.0
General Surgey	Appendicectomy – Lap.	12295.0
General Surgey	Appendicectomy – Open	12789.9
General Surgey	CystoJejunostomy – Open	18945.9
General Surgey	Epididymal Cyst exision	7365.6
General Surgey	Epididymal Nodule excision	12867.9
General Surgey	Hernia - Ventral(Epigastric)	12745.1
General Surgey	Incision & Drainage of Abscess	7136.1

General Surgey	Inguinal Node (dissection) - U/L	14340.8
General Surgey	Operation for Hydrocele (U/L)	10238.7
General Surgey	Operative Cholecystostomy – Lap.	13750.1
General Surgey	Operative Cholecystostomy – Open	18223.8
General Surgey	Operative drainage of Appendicular Abscess	12831.7
General Surgey	Pyloroplasty	23113.3
General Surgey	Repair of Incisional Hernia	13454.9
General Surgey	Hernia - Ventral(Spigelian)	19294.0
General Surgey	Splenectomy - Open	20147.2
General Surgey	Hernia - Ventral(Umbilical)	12451.0
General Surgey	Intercostal drainage Only	6637.9
General Surgey	Foreign Body Removal	8341.4
General Surgey	Biopsy - Lymph Node	5064.6
General Surgey	Closure of Burst Abdomen	12431.1
General Surgey	Free Grafts - Wolfe Grafts	10951.8
General Surgey	Split thickness skin grafts(Large (> 8% TBSA)	22064.9
General Surgey	Split thickness skin grafts(Small (< 4% TBSA)	10622.6
General Surgey	Split thickness skin grafts (Medium (4 - 8% TBSA)	17292.2
General Surgey	Breast Lump Excision (Benign)	10558.2
General Surgey	Flap Reconstructive Surgery	6574.0
General Surgey	Gastrojejunostomy	15100.3
General Surgey	Oesophagectomy	10469.3
General Surgey	PancreaticoDuodenectomy (Whipple's)	26223.5
General Surgey	Simple Mastectomy	12799.7
General Surgey	Rectal Polyp Excision	8345.0
General Surgey	Radical / Modified Radical Mastectomy	24331.7
General Surgey	Radical Neck Dissection	7068.6
General Surgey	Colostomy	11507.7
General Surgey	Distal Pancreatectomy with Pancreatico Jejunostomy	43055.3
General Surgey	Orchidectomy	9258.3
General Surgey	Hemi thyroidectomy	18910.8
General Surgey	Total thyroidectomy	16846.9
General Surgey	Foreign Body Removal with scope	7976.2
General Surgey	Superficial Parotidectomy	17476.3
Opthal	Canaliculo Dacryocystorhinostomy without Silicon Tube / St	15051.2
Opthal	Canaliculo Dacryocystorhinostomy with Silicon Tube / Stent	7114.3
Opthal	Capsulotomy (YAG)	15561.8
Opthal	Cataract with foldable / non-foldable IOL using SICS techniq	9934.0
Opthal	Cataract with foldable hydrophobic acrylic IOL by Phaco em	9476.2
Opthal	Conjunctival tumour excision including Amniotic Membrane	6090.5
Opthal	Corneo / Scleral / Corneo scleral tear repair	3123.6
Opthal	Entropion correction	13080.7
Opthal	Enucleation without implant	5810.5
Opthal	Evisceration	14156.0
Opthal	Glaucoma Surgery (Trabeculectomy only) with or without M	11111.4
Opthal	Iridectomy	13475.1
Opthal	IRIS Prolapse – Repair	5976.3
Opthal	Lid Tear Repair	2960.1
Opthal	Lid Tumor excision + Lid Reconstruction	6892.2

Opthal	Limbal Dermoid Removal	4793.6
Opthal	Paediatric lensectomy	4925.0
Opthal	Pterygium + Conjunctival Autograft	9005.5
Opthal	Exenteration	8078.7
Ortho	Single Stage Amputation Above Knee	28689.0
Ortho	Amputation – Fingers	17659.2
Ortho	Amputation - Toes	16126.1
Ortho	Application of P.O.P. casts for Upper Limbs	9700.1
Ortho	Application of P.O.P. Spikas & Jackets	9762.8
Ortho	Application of Skeletal Tractions with pin	7212.8
Ortho	Application of Skin Traction	4103.6
Ortho	Arthorotomy of any joint	11260.6
Ortho	Arthroscopic Meniscus Repair / Meniscectomy	12643.1
Ortho	Single Stage Amputation Below Elbow	19435.2
Ortho	Single Stage Amputation Below Knee	18939.6
Ortho	Bipolar Hemiarthroplasty(Non Modular)	13687.2
Ortho	Bone grafting for Non union	8750.1
Ortho	Bone Tumour Excision (malignant) including GCT + Joint rep	15789.6
Ortho	Closed Reduction and Percutaneous Screw Fixation (neck/fe	16187.4
Ortho	Comminuted Fracture - Olecranon of Ulna – Plating	17262.0
Ortho	Correction of club foot per cast	14122.6
Ortho	Fixation of Diaphyseal Fracture - Long Bone (Closed Reducti	7620.9
Ortho	Diaphyseal Fracture - Long Bone (Open reduction internal fi	13214.2
Ortho	Disarticulation Hind quarter	8360.5
Ortho	Displaced Clavicle Fracture (Open Reduction Internal Fixatic	14130.0
Ortho	Dorsal and lumber spine fixation(Posterior)	12362.4
Ortho	Duputryen's Contracture release + rehabilitation	12045.3
Ortho	Arthrolysis of joint Elbow	23083.1
Ortho	Elbow replacement	26723.7
Ortho	Excision Arthoplasty of Femur head	3881.0
Ortho	Exploration and Ulnar nerve Repair	10288.7
Ortho	Single Stage Amputation Foot	7185.1
Ortho	External fixation of fracture - Both bones(forearm)	23492.3
Ortho	Fracture - Acetabulum - Single Approach	23266.7
Ortho	Fracture - Both Bones - Forearm - ORIF - Plating / Nailing	16387.0
Ortho	Fracture - Long Bones - Metaphyseal – ORIF	23396.3
Ortho	Fracture - Single Bone - Forearm - ORIF - Plating / Nailing	17151.5
Ortho	Fracture Head radius – Excision	14008.3
Ortho	Fracture intercondylar Humerus + olecranon osteotomy	20998.3
Ortho	Closed reduction of joint dislocation(hip)	15735.0
Ortho	Fracture Condyle - Humerus - ORIF(Lateral Condyle)	14964.4
Ortho	Internal Fixation of Small Bones	18670.9
Ortho	External fixation of Fracture Long Bone	15939.5
Ortho	Open Reduction of Small Joint	12572.7
Ortho	Patellectomy	25421.2
Ortho	Total Hip Replacement Cemented	22657.4
Ortho	Primary - Total Knee Replacement	25104.8
Ortho	Closed reduction of joint dislocation Shoulder	9565.7
Ortho	Osteotomy Small Bone	23746.4

Ortho	External fixation of Fracture Small Bone	10687.7
Ortho	Tendon Grafting	25286.3
Ortho	Tendon Release / Tenotomy	17663.3
Ortho	Tendon Repair	7278.1
Ortho	Tenolysis	5891.1
Ortho	Tension Band Wiring	16344.5
Ortho	Ankle / Triple without implant	8281.3
Ortho	Unipolar Hemiarthroplasty	22071.7
Ortho	Single Stage Amputation Wrist	16790.6
Ortho	Open Reduction Internal Fixation(Ankle Fractures)	21892.0
Ortho	Bone Tumour Excision + reconstruction	12606.6
Ortho	Bone Tumour (benign) curettage	34477.2
Ortho	Anti-biotic + dressing - minimum of 5 sessions	13924.6
Ortho	Intertrochanteric Fracture with Dynamic Hip Screw	20425.2
Ortho	Excision of Osteochondroma	13897.5
Ortho	External fixation of Fracture Pelvis	17822.5

Supplementary Material 4				
**Annual financial pay-out for district hospitals when upgraded to medical college**				
**State Name**	**District**	**District Hospital (DH)**	**Annual pay-out (₹) when upgraded to Medical College**	**Annual pay-out (USD) when upgraded to Medical College**
Andaman and Nicobar Islands	Nicobars	BJR Hospital	361057144	4374329
Andaman and Nicobar Islands	North & Middle Andaman	Dr R.P. Hospital	182502962	2211085
Andaman and Nicobar Islands	South Andaman	G.B. Pant Hospital	1270386268	15391159
Andhra Pradesh	Anantapur	Ggh Anantapur	1341882560	16257361
Andhra Pradesh	Chittoor	GovernmentMaternity Hospl.Th	813695531	9858196
Andhra Pradesh	Chittoor	Sri.Venkateshwara Ram Narayana Ruia Gen. Hospital	2905474093	35200801
Andhra Pradesh	Cuddapah	DH Proddutur	949092300	11498574
Andhra Pradesh	East Godavari	DH Rajahmundry	682431112	8267884
Andhra Pradesh	Guntur	DH Tenali	734539927	8899199
Andhra Pradesh	Krishna	DH Machilipatnam	944117432	11438302
Andhra Pradesh	Kurnool	DH Nandyal	853152437	10336230
Andhra Pradesh	Nellore	Government General Hospital Nellore	2887356641	34981302
Andhra Pradesh	Prakasam	Rims Ongole Th	1339314886	16226253
Andhra Pradesh	Srikakulam	Rims Srikakulam Th	1340799323	16244237
Andhra Pradesh	Visakhapatnam	King George Hospital Th	5381884764	65203353
Andhra Pradesh	Vizianagaram	DH Vizianagaram	819553036	9929162
Andhra Pradesh	West Godavari	DH Eluru	945040190	11449481
Arunachal Pradesh	East Siang	GH Pasighat	416772921	5049345
Arunachal Pradesh	Lohit	GH Tezu	202149216	2449106
Arunachal Pradesh	Lower Dibang Valley	DH Roing	211631324	2563985
Arunachal Pradesh	Papum Pare	Tomo Riba Institute Of Medical Science & Hospital	690228184	8362348
Arunachal Pradesh	Tawang	DH Tawang	122869006	1488600
Arunachal Pradesh	West Siang	General Hospital Aalo	231237459	2801520
Assam	Baksa	Dr Ravi Boro Civil Hospital Baksa	213209154	2583101
Assam	Barpeta	Barpeta Civil Hospital Kalgachia	146273048	1772147
Assam	Bongaigaon	Bongaigaon Ch	447840193	5425735
Assam	Cachar	S.M.Deb Civil Hospital Silchar	256981280	3113415
Assam	Chirang	J.S.B Civil Hospital Chirang	290363010	3517846
Assam	Darrang	Mangaldai Civil Hospital	558308035	6764091
Assam	Dhemaji	DHemaji Civil Hospital	450849185	5462190
Assam	Dhubri	DHubri Civil Hospital	554817603.4	6721803
Assam	Dima Hasao	Haflong Civil Hospital	397687687.7	4818121
Assam	Goalpara	200 Bedded Civil Hospital	552972088	6699444
Assam	Golaghat	Kushal Konwar Civil Hospital	886649622.2	10742060
Assam	Hailakandi	S.K.Roy Civil Hospital	310128966.5	3757317
Assam	Kamrup Metropolitan	Sonapur District Hospital	159299190.2	1929964
Assam	Kamrup	Trb Civil Hospital	133688224.7	1619678
Assam	Karbi Anglong	Diphu Civil Hospital	560835588.4	6794713
Assam	Karimganj	Karimganj Civil Hospital	486035324.1	5888482

Assam	Kokrajhar	Rnb Civil Hospital Kokrajhar	553814605.9	6709651
Assam	Lakhimpur	North Lakhimpur Civil Hospital	608807807.4	7375912
Assam	Morigaon	Morigaon Civil Hospital	429450809.5	5202942
Assam	Nagaon	B.P. Civil Hospital	828125540.8	10033021
Assam	Nalbari	Smk Civil Hospital	644435922.5	7807559
Assam	Sivasagar	Sivasagar Civil Hospital	845270878.5	10240742
Assam	Sonitpur	Kanaklata Civil Hospital	718740936.3	8707789
Assam	Tinsukia	LGB Civil Hospital	424034623	5137323
Assam	Udalguri	Udalguri Civil Hospital	349863123	4238710
Bihar	Araria	Sardar Hospital Araria	293171402.9	3551871
Bihar	Arwal	Sadar Hospital Arwal	284745895.1	3449793
Bihar	Aurangabad	Sadar Hospital Aurangabad	330150111.7	3999880
Bihar	Banka	Sadar Hospital Banka	356202396	4315512
Bihar	Begusarai	Sadar Hospital Begusarai	312737417.5	3788920
Bihar	Bhagalpur	LNJP Sadar Hospital Bhagalpur	109481455.8	1326405
Bihar	Bhojpur	Sadar Hospital Ara Bhojpur	423031625.5	5125171
Bihar	Buxar	Sadar Hospital Buxar	292409124.8	3542635
Bihar	East Champaran	Sadar Hospital Motihari Purbi Champaran	450086907.4	5452955
Bihar	Gaya	Sadar Hospital Pilgrim Gaya	188199987.4	2280106
Bihar	Gopalganj	Sadar Hospital Gopalganj	304552629.1	3689758
Bihar	Jamui	Sadar Hospital Jamui	292810323.8	3547496
Bihar	Jehanabad	Sadar Hospital Jehanabad	292409124.8	3542635
Bihar	Kaimur	Sadar Hospital Bhabua Kaimur	298815154.7	3620247
Bihar	Katihar	Sadar Hospital Katihar	347281966.5	4207438
Bihar	Khagaria	Sadar Hospital Khagaria	292088165.6	3538747
Bihar	Kishanganj	Sadar Hospital Kishanganj	293251642.7	3552843
Bihar	Lakhisarai	Sadar Hospital Lakhisarai	226556255.7	2744806
Bihar	Madhepura	Sadar Hospital Madhepura	240384795.9	2912343
Bihar	Madhubani	Sadar Hospital Madhubani	320106325	3878196
Bihar	Munger	Sadar Hospital Munger	448843190.5	5437887
Bihar	Muzaffarpur	Sadar Hospital Muzaffarpur	459488775.7	5566862
Bihar	Nalanda	Sadar Hospital Biharsharif Nalanda	816383564.2	9890763
Bihar	Nawada	Sadar Hospital Nawada	245426749.2	2973428
Bihar	Purnia	Sadar Hospital Purnia	818670398.5	9918469
Bihar	Rohtas	Sadar Hospital Rohtas Sasaram	292930683.5	3548954
Bihar	Saharsa	Sadar Hospital Saharsa	766619432.6	9287854
Bihar	Samastipur	Sadar Hospital Samastipur	286604893.4	3472315
Bihar	Saran	Sadar Hospital Saran	382789721.9	4637627
Bihar	Sheikhpura	Sadar Hospital Sheikhpura	247807306.2	3002269
Bihar	Sheohar	Sadar Hospital Sheohar	230501488.8	2792603
Bihar	Sitamarhi	Sadar Hospital Sitamarhi	224162215.8	2715801
Bihar	Siwan	Sadar Hospital Siwan	291285767.6	3529026
Bihar	Supaul	Sadar Hospital Supaul	250789661.7	3038402
Bihar	Vaishali	Sadar Hospital Hajipur Vaishali	385691837.6	4672787
Bihar	West Champaran	Sadar Hospital M.J.K Bettiah Paschim Champaran	1262401750	15294424
Chandigarh	Chandigarh	GMSH 16	1625987983	19699394
Chhattisgarh	Balod	DH Balod	294415119.8	3566939

Chhattisgarh	Baloda Bazar	DH Baloda Bazar	294374999.9	3566453
Chhattisgarh	Bemetara	DH Bemetara	190767661	2311215
Chhattisgarh	Bilaspur	Bilaspur DH	533316596.9	6461311
Chhattisgarh	Dantewada	Dantewada	491010849.3	5948762
Chhattisgarh	Dhamtari	DHamtari	621111791.4	7524979
Chhattisgarh	Durg	District Hospital Durg	1105446126	13392853
Chhattisgarh	Gariaband	DH Gariaband	191128740.1	2315589
Chhattisgarh	Janjgir-Champa	District Hospital	276106304.9	3345121
Chhattisgarh	Jashpur	Jashpur	362287466.7	4389235
Chhattisgarh	North Bastar Kanker	Kanker DH	455101894.9	5513713
Chhattisgarh	Kawardha	District Hospital Kawardha	297223512.8	3600963
Chhattisgarh	Kondagaon	Ravindra Tagore DH Kondagaon	465292678.3	5637178
Chhattisgarh	Korba	Indira Gandhi Dstt Hospital Korba	331193229.1	4012518
Chhattisgarh	Mahasamund	Mahasamund	464396886.4	5626325
Chhattisgarh	Mungeli	DH Mungeli	191208979.9	2316561
Chhattisgarh	Narayanpur	Narayanpur	301075023.2	3647626
Chhattisgarh	Raipur	Raipur	326650443.7	3957481
Chhattisgarh	Sukma	DH Sukma	297263632.7	3601449
Dadra and Nagar Haveli	Dadra & Nagar Haveli	Shri Vinoba Bhave Civil Hospital	855154514.4	10360486
Daman and Diu	Daman	Government Hospital Daman	470241566.4	5697136
Daman and Diu	Diu	Government Hospital Diu	183225119.8	2219834
Delhi	Central	Aruna Asaf Ali Hospital	485854007.9	5886286
Delhi	Central	Girdhari Lal Maternity Hospital	354790188.2	4298403
Delhi	Central	Kasturba Hospital	1280100755	15508853
Delhi	East	Lal Bahadur Shastri Hospital	593005380.8	7184461
Delhi	North	Babu Jagjeevan Ram Memorial Hospital Jahgirpuri	362172578.6	4387843
Delhi	North	Maharishi Valmiki Hospital	493236398.2	5975726
Delhi	North	Satyawati Raja Harishchandra Hospital	624300217.9	7563608
Delhi	North East	DH Jpc Hospital	682128778.2	8264221
Delhi	North West	Deep Chand Bandhu Hospital	624219978.1	7562636
Delhi	North West	Sanjay Gandhi Memorial Hospital Mangolpuri	886347617.5	10738401
Delhi	North West	Bhagwan Mahavir Hospital Pitampura	951879527.3	11532342
Delhi	South	Pt. Madan Mohan Malviya Hospital	370397486.8	4487491
Delhi	South West	Rao Tula Ram Hospital	362373178.1	4390274
Delhi	West	Guru Govind Singh Govt Hospital	362132458.7	4387357
Delhi	West	Acharya Shree Bhikshu Hospital	571834570.2	6927969
Delhi	West	Deendayal Upadhyay Hospital	1777621711	21536488
Delhi	Shahdara	Hedgewar Hospital	624300217.9	7563608
Delhi	Shahdara	DH SDN Hospital	1069917205	12962409
Goa	North Goa	North Goa District Hospital	638658656.9	7737566
Goa	South Goa	South Goa District Hospital	625231315.7	7574889
Gujarat	Amreli	General Hospital Amreli	603953299.5	7317098
Gujarat	Anand	S.S.Hospital Petlad	378982248.8	4591498
Gujarat	Banaskantha	General Hospital Palanpur	873142041.3	10578411
Gujarat	Bharuch	General Hospital Bahruch	580749528.1	7035977
Gujarat	Botad	Botad	197641975.6	2394499
Gujarat	Chhotaudepur	Chhotaudepur	296180395.4	3588326

Gujarat	Dahod	General Hospital Dahod	1019015008	12345711
Gujarat	Devbhumi Dwarka	Jam Khambhalia	419902273.3	5087258
Gujarat	Kheda	General Hospital Nadiad	451651583.5	5471912
Gujarat	Mahesana	General Hospital Mehsana	590993914.2	7160091
Gujarat	Mahisagar	Lunawada	163672259.3	1982945
Gujarat	Morbi	Morbi	568659626.5	6889504
Gujarat	Narmada	General Hospital Rajpipla	245453386.2	2973751
Gujarat	Navsari	M.G.G.Hospital Navsari	628842106.7	7618635
Gujarat	Panchmahal	General Hospital Godhra	724665527.4	8779568
Gujarat	Porbandar	Bhavsinhji General Hospital	657234828.2	7962622
Gujarat	Rajkot	PK General Hospital	361074961.8	4374545
Gujarat	Surendranagar	M.G. General Hospital	318341049.4	3856809
Gujarat	Tapi	General Hospital Vyara	646201198.1	7828946
Gujarat	Dang	General Hospital Dang	553934965.6	6711109
Gujarat	Vadodara	Jamnabai General Hospital	589861320.3	7146369
Haryana	Ambala	Civil Hospital	552009210.4	6687778
Haryana	Bhiwani	Civil Hospital	815942245.3	9885416
Haryana	Faridabad	B.K. Civil Hospital	588898442.7	7134704
Haryana	Fatehabad	Civil Hospital Fatehabad	292609724.3	3545066
Haryana	Hisar	Civil Hospital	491465651.1	5954272
Haryana	Jhajjar	Civil Hospital Jhajjar	292449244.7	3543121
Haryana	Jind	Civil Hospital Jind	266798159.3	3232350
Haryana	Kaithal	IGMS Civil Hospital	488764134.9	5921543
Haryana	Kurukshetra	LNJP Civil Hospital	270195196.7	3273506
Haryana	Mahendragarh	Civil Hospital	289841451.2	3511527
Haryana	Mewat	Civil Hospital Mandikhera	162200977.1	1965120
Haryana	Palwal	Civil Hospital	297584591.9	3605338
Haryana	Panchkula	Civil Hospital	813936250.3	9861113
Haryana	Panipat	Civil Hospital	247526466.9	2998867
Haryana	Rewari	Civil Hospital Rewari	315800012.8	3826024
Haryana	Rohtak	Civil Hospital	292409124.8	3542635
Haryana	Sirsa	Civil Hospital Sirsa	343390336.2	4160290
Haryana	Sonipat	Civil Hospital Sonepat	554817603.4	6721803
Himachal Pradesh	Bilaspur	Bilaspur RH	774474025.1	9383015
Himachal Pradesh	Chamba	Chamba RH MCH Centre	815139847.3	9875695
Himachal Pradesh	Hamirpur	Hamirpur RH	634890974.9	7691919
Himachal Pradesh	Kangra	DHaramshala ZH	590967277.2	7159768
Himachal Pradesh	Kullu	Kullu RH	816263204.5	9889305
Himachal Pradesh	Lahul and Spiti	Keylong RH	81623775.97	988900
Himachal Pradesh	Mandi	Mandi ZH	813214092.1	9852364
Himachal Pradesh	Shimla	DDU ZH	747472346	9055880
Himachal Pradesh	Sirmaur	Nahan RH	683634708.7	8282466
Himachal Pradesh	Solan	Solan RH	497657927.4	6029294
Himachal Pradesh	Una	Una RH	549963095.5	6662989
Jammu and Kashmir	Anantnag	MCH Anantnag	317538651.4	3847088
Jammu and Kashmir	Anantnag	DH Anantnag	495785446.2	6006608
Jammu and Kashmir	Badgam	District Hospital Budgam	196438378.6	2379917

Jammu and Kashmir	Bandipore	Bandipora	82947732.67	1004940
Jammu and Kashmir	Baramulla	Baramula	555258922.3	6727150
Jammu and Kashmir	Doda	Doda	447800073.1	5425249
Jammu and Kashmir	Ganderbal	District Hospital	135533740.1	1642037
Jammu and Kashmir	Jammu	Gandhinagar Hospital	415787323.9	5037404
Jammu and Kashmir	Jammu	Sarwal Hospital	213949041.6	2592065
Jammu and Kashmir	Kathua	Kathua	447238394.5	5418444
Jammu and Kashmir	Kulgam	Kulgam	122240241.6	1480982
Jammu and Kashmir	Kupwara	DH Handwara	161626144.4	1958155
Jammu and Kashmir	Pulwama	Pulwama DH	170466334.1	2065257
Jammu and Kashmir	Ramban	Ramban	132564867.5	1606068
Jammu and Kashmir	Reasi	DH Reasi	158015353.4	1914409
Jammu and Kashmir	Samba	Samba	212085797.2	2569491
Jammu and Kashmir	Shopian	Shopain	112156335	1358812
Jammu and Kashmir	Srinagar	District Hospital Jnlm	455387309.1	5517171
Jammu and Kashmir	Udhampur	Udhampur	551367292	6680001
Jharkhand	Bokaro	Bokaro Sadar Hospital	324764808.4	3934635
Jharkhand	Chatra	Chatra Sadar Hospital	102058945.5	1236479
Jharkhand	Deoghar	Deoghar Sadar Hospital	292529484.5	3544094
Jharkhand	Dumka	Dumka Sadar Hospital	300634033	3642283
Jharkhand	Garhwa	Garhwa Sadar Hospital	294214520.3	3564508
Jharkhand	Giridih	Giridih Sadar Hospital	291967805.9	3537289
Jharkhand	Godda	Godda Sadar Hospital	293251642.7	3552843
Jharkhand	Gumla	Gumla Sadar Hospital	509881342.7	6177385
Jharkhand	Hazaribagh	Hazaribagh Sadar Hospital	883854712.1	10708199
Jharkhand	Jamtara	Jamtara Sadar Hospital	293492362.1	3555759
Jharkhand	Khunti	Khunti Sadar Hospital	188601186.4	2284967
Jharkhand	Koderma	Kodrma Sadar Hospital	291285767.6	3529026
Jharkhand	Latehar	Latehar Sadar Hospital	189965263	2301493
Jharkhand	Lohardaga	Lohardaga Sadar Hospital	163511779.7	1981000
Jharkhand	Pakur	Pakur Sadar Hospital	292770203.9	3547010
Jharkhand	Palamu	Palamau Sadar Hospital	556261919.8	6739301
Jharkhand	West Singhbhum	Pashchimi Singhbhum Sadar Hospital	293973800.9	3561592
Jharkhand	East Singhbhum	Purbi Singhbhum Sadar Hospital	288517494.5	3495487
Jharkhand	Ramgarh	Ramgarh Sadar Hospital	133126546.1	1612873
Jharkhand	Ranchi	Ranchi Sadar Hospital	587614605.9	7119150
Jharkhand	Sahebganj	Sahibganj Sadar Hospital	188400586.9	2282537
Jharkhand	Saraikela Kharsawan	Saraikela Sadar Hospital	238138081.5	2885123
Jharkhand	Simdega	Simdega Sadar Hospital	294615719.3	3569369
Karnataka	Bagalkot	Bagalkote District Hospital FRU	815581166.2	9881042
Karnataka	Bengaluru Urban	HSIS Goshiya	415560984	5034662
Karnataka	Bengaluru Urban	Indiranagar General Hospital	625263095.5	7575274
Karnataka	Bengaluru Urban	Jayanagar General Hospital	848071589	10274674
Karnataka	Bengaluru Urban	Vanivilas Hospital	1506011964	18245844
Karnataka	Bengaluru Urban	Victoria Hospital	2103662982	25486588
Karnataka	Bengaluru Urban	Kc General Hospital	1044667319	12656498
Karnataka	Bengaluru Urban	Bowring Lady Curzon	2800922502	33934123

Karnataka	Belagavi	Belgaum District Hospital	1999533187	24225020
Karnataka	Ballari	Bellary District Hospital FRU	582715403.2	7059794
Karnataka	Ballari	Vims Bellary Medical College	2698085453	32688217
Karnataka	Bidar	Bidar District Hospital	1205763633	14608234
Karnataka	Bijapur	Bijapur District Hospital FRU	687847298.2	8333503
Karnataka	Chamarajanagar	Chamarajnagar District Hospital FRU	813775770.7	9859169
Karnataka	Chikballapur	Chikkaballapur District Hospital FRU	395735624.3	4794471
Karnataka	Chikkamagaluru	Chickmagalur District Hospital FRU	1073576456	13006742
Karnataka	Chitradurga	Chitradurga District Hospital FRU	1208772625	14644689
Karnataka	Dakshina Kannada	Lady Goshan Hospital Mangalore DH FRU	733920727.8	8891698
Karnataka	Dakshina Kannada	Wenlock Hospital Mangalore DH	2393188685	28994290
Karnataka	Davanagere	Davangere Women And Children DH FRU	291045048.2	3526109
Karnataka	Davanagere	Davanagere District Hospital	2466704455	29884958
Karnataka	Dharwad	DHarwad District Hospital FRU	717675428.1	8694880
Karnataka	Dharwad	Hubli Kims District Hospital	3207888002	38864647
Karnataka	Gadag	Gadag District Hospital FRU	687365859.4	8327670
Karnataka	Gulbarga	Gulbarga District Hospital FRU	1529548952	18531003
Karnataka	Hassan	Hassan District Hospital	1991665112	24129696
Karnataka	Haveri	Haveri District Hospital FRU	815781765.7	9883472
Karnataka	Kodagu	Kodagu District Hospital FRU	1097542505	13297098
Karnataka	Kolar	Kolar District Hospital FRU	1075622571	13031531
Karnataka	Koppal	Koppal District Hospital FRU	819312316.9	9926246
Karnataka	Mandya	Mandya District Hospital	1731222508	20974346
Karnataka	Mysuru	Cheluvamba Hospital Mysore DH	1134552097	13745482
Karnataka	Mysuru	KR Hospital Mysore DH	2733530698	33117648
Karnataka	Raichur	Raichur District Hospital	1709663653	20713153
Karnataka	Ramanagara	Ramanagara District Hospital FRU	289881571.1	3512013
Karnataka	Shivamogga	Shimoga District Hospital	2515438952	30475393
Karnataka	Tumakuru	Tumkur District Hospital FRU	1077066887	13049029
Karnataka	Udupi	Udupi District Hospital FRU	677215524.7	8204695
Karnataka	Uttara Kannada	Uttara Kannada District Hospital FRU	853670078.1	10342502
Karnataka	Yadgir	Yadgir District Hospital FRU	292569604.4	3544580
Kerala	Alappuzha	W And C Hospital Alappuzha	687994952.5	8335291
Kerala	Alappuzha	General Hospital Alappuzha	1068080030	12940151
Kerala	Ernakulam	DH Aluva	614358494.1	7443161
Kerala	Ernakulam	GH Ernakulam	1694324368	20527312
Kerala	Idukki	District Hospital Thodupuzha	398959028	4833523
Kerala	Kannur	GH Thalassery	1472322519	17837685
Kerala	Kasaragod	DH Kanhangad	974315877.9	11804166
Kerala	Kollam	W&C Hospital Kollam	769379126.6	9321288
Kerala	Kollam	DH Kollam	1461396094	17705308
Kerala	Kottayam	General Hospital Kottayam	963429573.3	11672275
Kerala	Kozhikode	General Hospital Calicut	1430221617	17327618
Kerala	Malappuram	DH Tirur	484061198.7	5864565
Kerala	Malappuram	GH Manjeri	1367431343	16566893
Kerala	Palakkad	W & C Palakkad	675610728.7	8185252
Kerala	Palakkad	District Hospital Palakkad	1446265988	17522001

Kerala	Pathanamthitta	General Hosp Pathanamthitta	1076746257	13045145
Kerala	Thiruvananthapuram	District Model Hospital Peroorkada	840113469.3	10178259
Kerala	Thiruvananthapuram	General Hospital Thiruvananthapuram	2011824017	24373928
Kerala	Thiruvananthapuram	W&C Hospital Thiruvananthapuram	1175636848	14243238
Kerala	Thrissur	GH Thrissur	682235087.2	8265509
Kerala	Wayanad	DH Mananthavady	797232560.2	9658742
Ladakh	Kargil	Kargil	452494101.4	5482119
Ladakh	Leh	Leh DH	684035907.7	8287326
Lakshadweep	Lakshadweep	Indira Gandhi Hospital	151074610.7	1830320
Madhya Pradesh	Agar Malwa	DH Agar	295418117.3	3579090
Madhya Pradesh	Alirajpur	DH Alirajpur	322673998.6	3909305
Madhya Pradesh	Anuppur	DH Anuppur	296942673.5	3597561
Madhya Pradesh	Ashoknagar	DH Ashoknagar	297343872.5	3602422
Madhya Pradesh	Balaghat	DH Balaghat	817707520.9	9906803
Madhya Pradesh	Barwani	DH Barwani	817587161.2	9905345
Madhya Pradesh	Betul	DH Betul	814939247.8	9873264
Madhya Pradesh	Bhind	DH Bhind	951298894.3	11525308
Madhya Pradesh	Bhopal	DH Bhopal J.P	926494901.8	11224799
Madhya Pradesh	Burhanpur	DH Burhanpur	555138562.6	6725691
Madhya Pradesh	Chhatarpur	DH Chhatarpur	825290182	9998670
Madhya Pradesh	Chhindwara	DH Chhindwara	1077227367	13050974
Madhya Pradesh	Damoh	DH Damoh	825731500.9	10004016
Madhya Pradesh	Datia	DH Datia	951138414.7	11523363
Madhya Pradesh	Dewas	DH Dewas	1077307607	13051946
Madhya Pradesh	Dhar	DH DHar	816744643.3	9895137
Madhya Pradesh	Dindori	DH Dindori	299831306.3	3632558
Madhya Pradesh	Guna	DH Guna	1083285472	13124370
Madhya Pradesh	Gwalior	DH Gwalior	591867315.3	7170673
Madhya Pradesh	Harda	DH Harda	293412122.3	3554787
Madhya Pradesh	Hoshangabad	DH Hoshangabad	815500926.4	9880069
Madhya Pradesh	Indore	DH Indore	322558213.9	3907902
Madhya Pradesh	Jabalpur	DH Jabalpur	1378932272	16706231
Madhya Pradesh	Jhabua	DH Jhabua	557024197.9	6748536
Madhya Pradesh	Katni	DH Katni	563764341.1	6830196
Madhya Pradesh	Khandwa	DH Khandwa	1080517199	13090831
Madhya Pradesh	Khargone	DH Khargone	817667401	9906317
Madhya Pradesh	Mandla	DH Mandla	821358431.8	9951035
Madhya Pradesh	Mandsaur	DH Mandsaur	1342885557	16269512
Madhya Pradesh	Morena	DH Morena	822000350.2	9958812
Madhya Pradesh	Narsinghpur	DH Narsinghpur	817988360.2	9910205
Madhya Pradesh	Neemuch	DH Neemuch	556703238.7	6744648
Madhya Pradesh	Panna	DH Panna	565449376.9	6850610
Madhya Pradesh	Raisen	DH Raisen	555499641.7	6730066
Madhya Pradesh	Rajgarh	DH Rajgarh	819633276.1	9930134
Madhya Pradesh	Ratlam	DH Ratlam	1374037644	16646931
Madhya Pradesh	Rewa	DH Rewa	299349867.5	3626725
Madhya Pradesh	Sagar	DH Sagar	822762628.3	9968047

Madhya Pradesh	Satna	DH Satna	1086414824	13162283
Madhya Pradesh	Sehore	DH Sehore	658424942.3	7977041
Madhya Pradesh	Seoni	DH Seoni	1079153122	13074305
Madhya Pradesh	Shahdol	DH Shahdol	824447664.1	9988462
Madhya Pradesh	Shajapur	DH Shajapur	553975085.5	6711595
Madhya Pradesh	Sheopur	DH Sheopur	302398979.9	3663666
Madhya Pradesh	Shivpuri	DH Shivpuri	822200949.7	9961242
Madhya Pradesh	Sidhi	DH Sidhi	824768623.3	9992351
Madhya Pradesh	Singrauli	DH Singrauli	351895755	4263336
Madhya Pradesh	Tikamgarh	DH Tikamgarh	562239784.9	6811725
Madhya Pradesh	Ujjain	DH Ujjain	1896607887	22978046
Madhya Pradesh	Umaria	DH Umaria	302198380.4	3661236
Madhya Pradesh	Vidisha	DH Vidisha	821278192	9950063
Maharashtra	Ahmednagar	Ahmednagar	743175270.6	9003820
Maharashtra	Amravati	District General Hospital Amravati	1053934448	12768772
Maharashtra	Amravati	District Women Hospital Amravati	555891932.8	6734819
Maharashtra	Beed	District Hospital Beed	864235423.5	10470504
Maharashtra	Bhandara	Bhandara	1291650472	15648782
Maharashtra	Buldhana	DH Buldana	828941750.5	10042910
Maharashtra	Gadchiroli	District Hospital Gadchiroli	691244335.6	8374659
Maharashtra	Hingoli	DH Hingoli	289199532.8	3503750
Maharashtra	Jalgaon	District Hospital Jalgaon	994126529.3	12044179
Maharashtra	Jalna	Women Hospital Jalna	184308357.1	2232958
Maharashtra	Jalna	District Hospital Jalna	551287052.2	6679029
Maharashtra	Nandurbar	Nandurbar	553894845.7	6710623
Maharashtra	Nashik	District Hospital Nashik	1477497986	17900388
Maharashtra	Osmanabad	District Hospital Osmanabad	643446407.9	7795571
Maharashtra	Osmanabad	WH Osmanabad	182101762.6	2206224
Maharashtra	Parbhani	General Hospital Parbhani	1091550829	13224507
Maharashtra	Parbhani	Women Hospital Parbhani	184589196.4	2236361
Maharashtra	Pune	Aundh	971846741	11774252
Maharashtra	Raigarh	Alibag	744753100.8	9022936
Maharashtra	Ratnagiri	District Hospital Ratnagiri	547756501	6636255
Maharashtra	Satara	Lt Karntisigh Nana Patil Civil Hospital Satara	657609390.2	7967160
Maharashtra	Sindhudurg	Sindhudurg	547636141.3	6634797
Maharashtra	Thane	District Hospital Thane	753259177.1	9125990
Maharashtra	Wardha	Wardha	766472435.9	9286073
Maharashtra	Washim	Washim	550605013.9	6670766
Manipur	Bishnupur	Bishnupur District Hospital	154966241	1877468
Manipur	Chandel	Chandel District Hospital	100882314.3	1222223
Manipur	Churachandpur	Churachandpur District Hospital	394545510.2	4780052
Manipur	Senapati	Senapati District Hospital	155768639	1887190
Manipur	Tamenglong	Tamenglong District Hospital	121397723.7	1470774
Manipur	Thoubal	Thoubal District Hospital	191102431.9	2315271
Manipur	Ukhrul	Ukhrul District Hospital	155086600.7	1878927
Meghalaya	East Garo Hills	Williamnagar Civil Hospital	288116295.5	3490626
Meghalaya	East Khasi Hills	Ganesh Das Hospital	1040529497	12606367

Meghalaya	East Khasi Hills	Shillong Civil Hospital	1601482645	19402504
Meghalaya	Ri Bhoi	Nongpoh DH	224857737	2724227
Meghalaya	West Garo Hills	Tura Maternity And Child Hospital	158215952.9	1916840
Meghalaya	West Garo Hills	Tura Civil Hospital	551407411.9	6680487
Meghalaya	West Jaintia Hills	Jowai Civil Hospital	317712285.1	3849192
Meghalaya	West Khasi Hills	Nongstoin DH	289841451.2	3511527
Meghalaya	West Khasi Hills	Mairang DH	289841451.2	3511527
Mizoram	Aizawl	Aizawl Civil Hospital	761149643.3	9221585
Mizoram	Champhai	Champhai DH	218023871.1	2641433
Mizoram	Kolasib	Kolasib DH	184829915.8	2239277
Mizoram	Lawngtlai	Lawngtlai DH	119164163.4	1443714
Mizoram	Lunglei	Lunglei DH	403158134.6	4884397
Mizoram	Mamit	Mamit DistrictHospital	97431674.14	1180418
Mizoram	Saiha	Saiha DH	149442520.1	1810547
Mizoram	Serchhip	Serchhip DH	160917469.1	1949570
Nagaland	Dimapur	District Hospital	420945390.7	5099896
Nagaland	Mokokchung	Ongpangkong DH	420423832	5093577
Nagaland	Wokha	Wokha DH	158376432.5	1918784
Odisha	Angul	Angul	461360928.1	5589544
Odisha	Balangir	Balangir	654038061.5	7923892
Odisha	Balasore	Balasore	972296071.2	11779696
Odisha	Bargarh	Bargarh	452413861.6	5481147
Odisha	Boudh	Boudh	278914697.9	3379146
Odisha	Bhadrak	Bhadrak	734642557.2	8900443
Odisha	Cuttack	City Hospital	402039023.6	4870839
Odisha	Deogarh	Deogarh	370699491.6	4491150
Odisha	Dhenkanal	DHenkanal	786412683.8	9527655
Odisha	Gajapati	Paralakhemundi	326900071	3960505
Odisha	Ganjam	City Hospital	392967022.4	4760928
Odisha	Jagatsinghapur	Jagatsinghpur	356670680.7	4321186
Odisha	Jajapur	Jajpur	829891145.2	10054412
Odisha	Jharsuguda	Jharsuguda	335553144.1	4065340
Odisha	Kalahandi	Bhawanipatna	472300506.7	5722080
Odisha	Kandhamal	Phulbani	524538917.9	6354966
Odisha	Kendrapara	Kendrapada	539424387.2	6535309
Odisha	Kenduajhar	Keonjhar	458980371.1	5560702
Odisha	Khordha	Capital Hospital	1795631061	21754677
Odisha	Khordha	Khordha	408975848.9	4954881
Odisha	Koraput	DHh Koraput	551339997.5	6679670
Odisha	Malkangiri	Malkangiri	367008132	4446428
Odisha	Mayurbhanj	Baripada	699389004.1	8473334
Odisha	Nabarangpur	Nabarangpur	393060416.3	4762060
Odisha	Nayagarh	Nayagarh	470776279.2	5703614
Odisha	Nuapada	Nuapada	350531678.4	4246810
Odisha	Puri	Puri	1021953326	12381310
Odisha	Rayagada	Rayagada	301222019.9	3649407
Odisha	Sambalpur	Sambalpur	622556107.8	7542478

Odisha	Sonepur	Subarnapur	322513519	3907360
Odisha	Sundargarh	Rgh Rourkela	854311667.7	10350275
Odisha	Sundargarh	Sundargarh	550243606.1	6666387
Puducherry	Karaikal	Government General Hospital	1350468876	16361387
Puducherry	Mahe	Government General Hospital	467968215	5669593
Puducherry	Puducherry	RGGW & CH	1236514520	14980791
Puducherry	Yanam	Government General Hospital	286030060.7	3465351
Punjab	Amritsar	Amritsar DH	584405013.9	7080264
Punjab	Barnala	Barnala DH	447318634.3	5419416
Punjab	Bathinda	Bathinda DH	551006212.9	6675627
Punjab	Faridkot	Faridkot DH	290082170.6	3514444
Punjab	Fatehgarh Sahib	Fatehgarh Sahib DH	234928489.5	2846238
Punjab	Fazilka	Fazilka DH	223667622.9	2709809
Punjab	Ferozepur	Ferozepur DH	343992134.7	4167581
Punjab	Gurdaspur	Gurdaspur DH	298240321.9	3613282
Punjab	Hoshiarpur	Hoshiarpur DH	549401416.9	6656184
Punjab	Jalandhar	Jalandhar DH	1291908921	15651913
Punjab	Kapurthala	Kapurthala DH	341143621.8	4133070
Punjab	Ludhiana	Ludhiana DH	767533282.4	9298925
Punjab	Mansa	Mansa DH	289640851.7	3509097
Punjab	Moga	Moga DH	342989137.2	4155429
Punjab	Sahibzada Ajit Singh Nagar	Mohali DH	548679258.7	6647435
Punjab	Sri Mukhtar Sahib	Muktsar DH	288718094	3497917
Punjab	Shahid Bhagat Singh Nagar	Nawanshahar DH	287755216.4	3486252
Punjab	Pathankot	Pathankot DH	417615439	5059552
Punjab	Patiala	M.K.H. Patiala DH	549682256.2	6659586
Punjab	Rupnagar	Rupnagar DH	286992938.3	3477016
Punjab	Sangrur	Sangrur DH	289480372.1	3507153
Punjab	Tarn Taran	Tarn Taran DH	290483369.6	3519304
Rajasthan	Ajmer	A K Hospital Beawar Ajmer	1114918997	13507621
Rajasthan	Alwar	Rajeev Gandhi Govt Genaral Hospital Alwar	1870363911	22660091
Rajasthan	Banswara	District Hospital Banswara	1223430200	14822270
Rajasthan	Baran	District Hospital Baran	909465348.8	11018480
Rajasthan	Barmer	District Hospital Barmar	786091395.8	9523763
Rajasthan	Bharatpur	RBM Hospital, Bharatpur	1412028258	17107200
Rajasthan	Bhilwara	M G Hospital Bhilwara	1412790536	17116435
Rajasthan	Bundi	Pandit Briz Sundar Sharma General Hospital Bundi	823765625.8	9980199
Rajasthan	Chittorgarh	District Hospital Chittaurgarh	1482227559	17957688
Rajasthan	Churu	D B Government Hospital Churu	813494931.4	9855766
Rajasthan	Dausa	District Hospital Dausa	690174252.4	8361694
Rajasthan	Dholpur	Sadar Hospital DHolpur	1085211227	13147701
Rajasthan	Dungarpur	Shri Hari Dev Joshi Genaral Hospital Dungarpur	706744428.7	8562448
Rajasthan	Sri Ganganagar	Govt Hospitls Sriganganagar	998669314.8	12099216
Rajasthan	Hanumangarh	DH Hanumangarh Town	832164825.4	10081958
Rajasthan	Jaisalmer	Jawahar Hospital Jaisalmer	427284334.9	5176694
Rajasthan	Jalore	District Hospital Jalor	432861001	5244257
Rajasthan	Jhunjhunu	B.D.K. Hospital Jhunjhunun	551367292	6680001

Rajasthan	Karauli	General Hospital Karauli	825370421.8	9999642
Rajasthan	Nagaur	District Hospital Nagaur	803731984	9737485
Rajasthan	Pali	Govt Bangur Hopital Pali	841312162.6	10192781
Rajasthan	Pratapgarh	District Hospital Pratapgarh	430453807	5215093
Rajasthan	Rajsamand	RK District Hospital Rajsamand	614063514.3	7439587
Rajasthan	Sawai Madhopur	General Hospital Sawai Madhopur	811688878.3	9833885
Rajasthan	Sikar	S K Hospital, Sikar	1074739933	13020838
Rajasthan	Sirohi	General Hospital Sirohi	562961943.1	6820474
Rajasthan	Tonk	District Sahadat Hospital Tonk	561678106.3	6804920
Sikkim	East Sikkim	Singtam Hospital	297972965.5	3610043
Sikkim	North Sikkim	Mangan Hospital	155969238.5	1889620
Sikkim	South Sikkim	Namchi District Hospital	285147422.9	3454657
Sikkim	West Sikkim	District Hospital Gyalshing	234045851.7	2835545
Tamil Nadu	Ariyalur	Ariyalur	633242141.6	7671943
Tamil Nadu	Chennai	Kilpauk Hospital	2974996827	36043092
Tamil Nadu	Coimbatore	Pollachi	878536494.5	10643767
Tamil Nadu	Cuddalore	Cuddalore	1990220796	24112198
Tamil Nadu	Dharmapuri	Pennagaram	423018471.4	5125012
Tamil Nadu	Dindigul	Dindigul	1714505336	20771812
Tamil Nadu	Erode	Erode	1892756376	22931383
Tamil Nadu	Kancheepuram	Kancheepuram	1450585455	17574333
Tamil Nadu	Kanniyakumari	Padhmanabapuram	424008314.8	5137004
Tamil Nadu	Karur	Kulithalai	293693290.3	3558193
Tamil Nadu	Krishnagiri	Krishnagiri	1131405016	13707354
Tamil Nadu	Madurai	Usilampatti	543200232.7	6581054
Tamil Nadu	Nagapattinam	Nagapattinam	1452297456	17595075
Tamil Nadu	Namakkal	Namakkal	1250138215	15145847
Tamil Nadu	The Nilgiris	Uthagamandalam	1005236153	12178776
Tamil Nadu	Perambalur	Perambalur	1274612340	15442359
Tamil Nadu	Pudukkottai	Aranthangi	464838534	5631676
Tamil Nadu	Ramanathapuram	Ramanathapuram	1629701733	19744387
Tamil Nadu	Salem	Mettur Dam	844085339.4	10226379
Tamil Nadu	Sivaganga	Karaikudi	605665301.1	7337840
Tamil Nadu	Thanjavur	Kumbakonam	1437055483	17410413
Tamil Nadu	Theni	Periakulam	800040953.2	9692767
Tamil Nadu	Thiruvallur	Thiruvallur	992330370.6	12022418
Tamil Nadu	Thiruvarur	Mannargudi	952195343.8	11536168
Tamil Nadu	Tiruchirappalli	Manapparai	608330943.6	7370135
Tamil Nadu	Tirunelveli	Tenkasi	727064142.3	8808628
Tamil Nadu	Tiruppur	Tiruppur	1925865846	23332516
Tamil Nadu	Tiruvannamalai	Cheyyar	650391725.6	7879716
Tamil Nadu	Thoothukudi	Kovilpatti	1105606934	13394802
Tamil Nadu	Vellore	Walajapet	844285938.9	10228810
Tamil Nadu	Viluppuram	Kallakurichi	821211763.9	9949258
Tamil Nadu	Virudhunagar	Virudhunagar	1036196548	12553871
Telangana	Hyderabad	Kingkoti	624099618.4	7561178
Telangana	Karimnagar	Karimnagar	1336466373	16191742

Telangana	Khammam	DH Khammam	682992790.3	8274689
Telangana	Nalgonda	Nalgonda	1206806750	14620872
Telangana	Sangareddy	DH Sangareddy	682029912.7	8263023
Telangana	Vikarabad	Tandur	979357502.4	11865247
Tripura	Dhalai	DHalai District Hospital	424636421.5	5144614
Tripura	Gomati	District Hospital Gomati District	420825031	5098437
Tripura	Khowai	Khowai District Hospital	291045048.2	3526109
Tripura	North Tripura	District Hospital North Tripura	296140275.5	3587840
Tripura	South Tripura	District Hospital South	288798333.8	3498889
Tripura	Unakoti	District Hospital Unakoti District	425037620.5	5149474
Uttar Pradesh	Agra	DH Male	405703088.6	4915230
Uttar Pradesh	Agra	DH Female	594434988.9	7201781
Uttar Pradesh	Aligarh	Pt Deen Dayal District Combined Hospital	335436701.8	4063929
Uttar Pradesh	Aligarh	Mohan Lal Gautam District Female Hospital	309223937.8	3746353
Uttar Pradesh	Aligarh	Malkhan Singh District Hospital	681445185.7	8255939
Uttar Pradesh	Ambedkar Nagar	Mahatma Jyotiba Phule District Hospital	294374999.9	3566453
Uttar Pradesh	Auraiya	District Combined Hospital Auraiya	166520772.2	2017455
Uttar Pradesh	Auraiya	District Combined Hospital Chicholi	297584591.9	3605338
Uttar Pradesh	Azamgarh	District Women Hospital	294575599.4	3568883
Uttar Pradesh	Azamgarh	District Hospital Azamgarh	606507490.2	7348043
Uttar Pradesh	Baghpat	District Combined Hospital	295578596.9	3581035
Uttar Pradesh	Bahraich	District Women Hosp	398195763.5	4824276
Uttar Pradesh	Bahraich	District Male Hosp	969634017.4	11747444
Uttar Pradesh	Ballia	District Male Hospitol Ballia	492629128.2	5968368
Uttar Pradesh	Ballia	District Fimale Hospitol Ballia	201667448.5	2443269
Uttar Pradesh	Balrampur	District Women Hospital	121035987	1466392
Uttar Pradesh	Balrampur	District Memeorial Male Hospital	236372148.3	2863728
Uttar Pradesh	Balrampur	District Combined Hospital	304525334.6	3689427
Uttar Pradesh	Banda	DWH Banda	117104236.8	1418757
Uttar Pradesh	Banda	DH Banda	308457413.5	3737066
Uttar Pradesh	Barabanki	DWH Barabanki	235623353.1	2854657
Uttar Pradesh	Barabanki	DH Barabanki	406006318.7	4918904
Uttar Pradesh	Bareilly	District Male Hospital	926748446.5	11227871
Uttar Pradesh	Bareilly	District Female Hospital	373659127.5	4527007
Uttar Pradesh	Basti	District Female Hospital	363076381.8	4398793
Uttar Pradesh	Basti	District Male Hospital	601612533.6	7288739
Uttar Pradesh	Basti	Opec Hospital Kaily	821799750.7	9956382
Uttar Pradesh	Bijnor	Pandit Deendayal Upadyaya District Combined Hospita	297624711.8	3605824
Uttar Pradesh	Bijnor	Pandit Deendayal Upadyaya District Combined Hospita	232092801.9	2811883
Uttar Pradesh	Budaun	District Female Hospital Budaun	253931839.2	3076470
Uttar Pradesh	Budaun	District Hospital Badaun	660229680.2	7998906
Uttar Pradesh	Bulandshahr	K.M.C Bulandshahr	195822768.4	2372459
Uttar Pradesh	Bulandshahr	Joint Hospital Sikandrabad	169610004.5	2054883
Uttar Pradesh	Bulandshahr	Ssmj Hospital Khurja	174852557.3	2118398
Uttar Pradesh	Bulandshahr	B.B.D.Government Hospital	502512106.5	6088104
Uttar Pradesh	Chandauli	Pt K P T DistrictCombined Hospital Chandauli	296421114.8	3591242
Uttar Pradesh	Chandauli	Rajkiya Mahila Chikitsalaya Mughalsarai	112931767.2	1368207

Uttar Pradesh	Chandauli	Combined Hospital Chakiya Chandauli	296421114.8	3591242
Uttar Pradesh	Chitrakoot	District Combined Hospital	302278620.2	3662208
Uttar Pradesh	Deoria	District Hospital Female	531506955.2	6439386
Uttar Pradesh	Deoria	District Hospital Male	633736734.5	7677935
Uttar Pradesh	Etah	Distt Male Hospital	304565454.5	3689913
Uttar Pradesh	Etah	District Women Hospital	89620790.19	1085786
Uttar Pradesh	Etawah	District Women Hospital F	149576033.9	1812164
Uttar Pradesh	Etawah	District Male Hospital	639754719.5	7750845
Uttar Pradesh	Faizabad	DistrictFemale Hospital	334804020.1	4056264
Uttar Pradesh	Faizabad	Sri Ram Ayodya	258787004.7	3135292
Uttar Pradesh	Faizabad	Distt Male Hospital	591689106.7	7168514
Uttar Pradesh	Farrukhabad	Dr Ram Manohar Lohiya Female	212580061.3	2575479
Uttar Pradesh	Farrukhabad	Dr Ram Manohar Lohiya Male	566452374.4	6862762
Uttar Pradesh	Farrukhabad	Civil Hospital Linziganj	120835387.5	1463962
Uttar Pradesh	Fatehpur	District Hospital Female	225552929.4	2732650
Uttar Pradesh	Fatehpur	District Hospital Male	419527382.6	5082716
Uttar Pradesh	Firozabad	District Women Hospital	158108418.5	1915537
Uttar Pradesh	Firozabad	Rnm District Joint Hospital	249853092.3	3027055
Uttar Pradesh	Gautam Buddha Nagar	Combined Distt Hospital Noida	391201746.8	4739541
Uttar Pradesh	Ghaziabad	District Women Hospital	209972596.6	2543889
Uttar Pradesh	Ghaziabad	District Combined Hospital Sanjay Nagar	293853441.2	3560134
Uttar Pradesh	Ghaziabad	District Mmg Male Hospital	466857683.1	5656139
Uttar Pradesh	Ghazipur	District Woman Hospital	243032709.3	2944423
Uttar Pradesh	Ghazipur	District Hospital	426522056.8	5167459
Uttar Pradesh	Gonda	DWH	392043936	4749745
Uttar Pradesh	Gonda	DH	496894991.7	6020051
Uttar Pradesh	Gorakhpur	Neta Ji Subhash Chandra Bose District Hospital	865055221.8	10480436
Uttar Pradesh	Gorakhpur	District Women Hospital	435165893.2	5272182
Uttar Pradesh	Hamirpur	District Women Hospital	147333565.7	1784996
Uttar Pradesh	Hamirpur	District Men Hospital	246942068.7	2991787
Uttar Pradesh	Hardoi	District Women Hospital	209878545.1	2542750
Uttar Pradesh	Hardoi	District Male Hospita	524431712.3	6353667
Uttar Pradesh	Hathras	Bagala Joint District Hospital, Hathras	222958290.1	2701215
Uttar Pradesh	Hathras	District Female Hospital Hathras	118107234.3	1430909
Uttar Pradesh	Jalaun	District Women Hospital	191048500.3	2314617
Uttar Pradesh	Jalaun	District Hospital	306384661.6	3711954
Uttar Pradesh	Jaunpur	District Femail Hospital	297664232.2	3603880
Uttar Pradesh	Jaunpur	District Male Hospital	415421669.9	5032974
Uttar Pradesh	Jhansi	District Women Hospital	192617422.6	2333625
Uttar Pradesh	Jhansi	District Hospital	520276971.8	6303331
Uttar Pradesh	Amroha	Amroha	300433104.8	3639849
Uttar Pradesh	Kannauj	Combined District Hospital Kannauj	300673824.2	3642765
Uttar Pradesh	Kanpur Dehat	District Combined Hospital	330060635.2	3998796
Uttar Pradesh	Kanpur Nagar	UHM Male Hospital	1174967635	14235130
Uttar Pradesh	Kanpur Nagar	Kpm Hospital Kanpur Nagar	244414515	2961164
Uttar Pradesh	Kanpur Nagar	Manyawar Kashiram Hospital	328295359.6	3977409
Uttar Pradesh	Kanpur Nagar	Distric Women Hospital	551103853	6676809

Uttar Pradesh	Kashi Ram Nagar	WH	123643780.5	1497986
Uttar Pradesh	Kaushambi	District Combined Hospital	302318740.1	3662694
Uttar Pradesh	Khushinagar	District Combined Hospital Kushinagar	294455239.7	3567425
Uttar Pradesh	Lakhimpur Kheri	DFH	199594039	2418149
Uttar Pradesh	Lakhimpur Kheri	DH	480070613.1	5816218
Uttar Pradesh	Lalitpur	District Female Hospital	196103607.7	2375861
Uttar Pradesh	Lalitpur	District Male Hospital	405805719.2	4916473
Uttar Pradesh	Lucknow	Shyama Prasad Mukherjee	1053011361	12757589
Uttar Pradesh	Lucknow	Balrampur Hospital Lucknow	1786968751	21649730
Uttar Pradesh	Lucknow	Awanti Bai Mahila Hospitals	921947541.4	11169706
Uttar Pradesh	Lucknow	Ram Manohar Lohiya	1288926237	15615777
Uttar Pradesh	Lucknow	Rani Laxmi Bai Combined Hospital	355751840.4	4310054
Uttar Pradesh	Lucknow	Lokbandhu Raj Narain	329539076.5	3992477
Uttar Pradesh	Lucknow	Jhalkari Bai Mahila Hospitals	282356101.4	3420840
Uttar Pradesh	Lucknow	RSM Combined Hospital	329539076.5	3992477
Uttar Pradesh	Lucknow	Bhau Rao Devras Hospital Mahanagar	329539076.5	3992477
Uttar Pradesh	Maharajganj	District Combined Hospital	296461234.7	3591728
Uttar Pradesh	Mahoba	DWH Mahoba	116101239.3	1406606
Uttar Pradesh	Mahoba	DH Mahoba	299590586.9	3629641
Uttar Pradesh	Mainpuri	District Female Hospital	118468313.4	1435284
Uttar Pradesh	Mainpuri	District Male Hospital	301957661	3658319
Uttar Pradesh	Mathura	District Women Hospital	212370553.9	2572941
Uttar Pradesh	Mathura	District Combined Hospital	332949268	4033793
Uttar Pradesh	Mathura	District Male Hospital	264796081.7	3208094
Uttar Pradesh	Maunathbhanjan	District Women Hospital	110163494.1	1334668
Uttar Pradesh	Maunathbhanjan	District Hospital	215014549.9	2604974
Uttar Pradesh	Meerut	District Women Hospital	372362136.6	4511293
Uttar Pradesh	Meerut	P.L. Sharma Hospital	723613173.3	8766818
Uttar Pradesh	Mirzapur	DistrictWoman Hospital Mzp	268616708.9	3254382
Uttar Pradesh	Mirzapur	District Hospital Mirzapur	444242227.3	5382145
Uttar Pradesh	Moradabad	Male District Hospital	562966189.3	6820526
Uttar Pradesh	Moradabad	Female District Hospital	277247062.4	3358942
Uttar Pradesh	Muzaffarnagar	Female District Hospital Muzaffar Nagar	367997975.4	4458420
Uttar Pradesh	Muzaffarnagar	Swami Kalyan Dev District Hospital Muzaffar Nagar	493819242.3	5982787
Uttar Pradesh	Pilibhit	District Male Hospital	378389358.3	4584315
Uttar Pradesh	Pilibhit	District Women Hospital	221112774.7	2678856
Uttar Pradesh	Pratapgarh	District Women Hospital	199781484.4	2420420
Uttar Pradesh	Pratapgarh	District Male Hospital	509092098.9	6167823
Uttar Pradesh	Prayagraj	Moti Lal Nehru District Hospital	481626381.3	5835066
Uttar Pradesh	Prayagraj	District Women Hospital	502596592.5	6089128
Uttar Pradesh	Prayagraj	Tej Bahadur Sapru Hospital	594341266.2	7200645
Uttar Pradesh	Rae Bareli	District Female Hospital	353313434.4	4280512
Uttar Pradesh	Rae Bareli	District Hospital	686215536.4	8313733
Uttar Pradesh	Rampur	District Male Hospital	431296324.9	5225301
Uttar Pradesh	Rampur	District Woman Hospital	174411238.4	2113051
Uttar Pradesh	Saharanpur	District Women Hospital	474627789.6	5750276
Uttar Pradesh	Saharanpur	SBD District Hospital	873061801.5	10577439

Uttar Pradesh	Sambhal	District Combined Hospital	227010400	2750308
Uttar Pradesh	Sant Kabir Nagar	District Combined Hospital Sant Kabir Nagar	295859436.2	3584437
Uttar Pradesh	Sant Ravidas Nagar	Maharaja Chet Singh District Hospital	299470227.2	3628183
Uttar Pradesh	Sant Ravidas Nagar	Maharaja Balavant Singh Distric Hospital	168406407.5	2040301
Uttar Pradesh	Shahjahanpur	District Women Hospital	326699142.7	3958071
Uttar Pradesh	Shahjahanpur	District Male Hospital	578341676.5	7006805
Uttar Pradesh	Shravasti	Combined District Hospital	306691809.2	3715675
Uttar Pradesh	Siddharth Nagar	District Combined Hospital	300152265.5	3636446
Uttar Pradesh	Sitapur	District Women Hospital	419580656.6	5083361
Uttar Pradesh	Sitapur	District Hospital Male Sitapur	566372134.6	6861790
Uttar Pradesh	Sonbhadra	District Combined Hospital Robertsganj	298066030.7	3611171
Uttar Pradesh	Sultanpur	District Women Hospital	249318379.5	3020576
Uttar Pradesh	Sultanpur	District Hospital	584841757.9	7085556
Uttar Pradesh	Unnao	Uma Shankar Female Hospital	193897013.2	2349128
Uttar Pradesh	Unnao	Uma Shanker Male Hospital	324960832.9	3937010
Uttar Pradesh	Varanasi	District Women Hospital Varanasi	539481907.4	6536006
Uttar Pradesh	Varanasi	Pt. Deen Dayal Upadhyay Govt Hospital	395311705.7	4789335
Uttar Pradesh	Varanasi	Lbs Ramnagar Hospital	468707444.7	5678549
Uttar Pradesh	Varanasi	S.S.P.G. Div. Dist. Hospital	817337205.1	9902317
Uttarakhand	Almora	Distt Female Hosptial	118749810.3	1438694
Uttarakhand	Almora	Distt Hosptial Almora	179039167.3	2169120
Uttarakhand	Bageshwar	Shyam Lal Shah DH	142742496.8	1729374
Uttarakhand	Chamoli	District Hospital	202389935.5	2452022
Uttarakhand	Champawat	DH Champawat	143785614.2	1742011
Uttarakhand	Pauri Garhwal	DH Pauri	372077379.9	4507843
Uttarakhand	Pauri Garhwal	DH Female Pauri	109949740.5	1332078
Uttarakhand	Haridwar	Cr Women Govt Hospital	129288189.9	1566370
Uttarakhand	Haridwar	Hmg Hospital Hardwar	213169034.5	2582615
Uttarakhand	Nainital	B.D.Pandey Male Hospital	190767989.8	2311219
Uttarakhand	Nainital	B.D.Pandey Female Hospital	164555225.9	1993642
Uttarakhand	Pithoragarh	H G Pant District Female Hospital	140321819.9	1700046
Uttarakhand	Pithoragarh	B D Pandey District Male Hospital	339538825.8	4113628
Uttarakhand	Rudraprayag	District Hospital Rudraprayag	155969238.5	1889620
Uttarakhand	Tehri Garhwal	DH Bauradi	225245781.9	2728929
Uttarakhand	Udham Singh Nagar	J.L.N. District Hospital	355614080.4	4308385
Uttarakhand	Uttarkashi	District Hospital	310330223.5	3759756
Uttarakhand	Uttarkashi	District Female Hospital	158296192.7	1917812
West Bengal	Alipurduar	Alipurduar District Hospital	765910757.3	9279268
West Bengal	Birbhum	Rampuhat DH & SSH	779444975.3	9443239
West Bengal	Dakshin Dinajpur	Balurghat DH & SSH	1155464460	13998842
West Bengal	Darjeeling	Siliguri DH	983597057.7	11916611
West Bengal	Darjeeling	Darjeeling DH	925928977	11217943
West Bengal	Howrah	Howrah District Hospital	1690753368	20484049
West Bengal	Hooghly	Imambara District Hospital	1663163045	20149782
West Bengal	Jalpaiguri	Jalpaiguri DH & SSH	1862085729	22559798
West Bengal	Jhargram	Jhargram DH & SSH	1233019514	14938448
West Bengal	Cooch Behar	Mjn District Hospital	1338913687	16221392

West Bengal	Nadia	District Hospital Nadia	2251853513	27281966
West Bengal	North 24 Parganas	Barasat DH	1597229936	19350981
West Bengal	North 24 Parganas	Basirhat DH & SSH	1463544840	17731340
West Bengal	Paschim Burdwan	Asansol DH & SSH	1123411591	13610511
West Bengal	Purba Medinipur	Tamluk District Hospital	1112975842	13484079
West Bengal	Purba Medinipur	Nandigram DH & SSH	208635485.8	2527689
West Bengal	Puruliya	D.M.Sadar DH & SSH	1610393838	19510466
West Bengal	South 24 Parganas	M. R. Bangur DH & SSH	1663363644	20152213
West Bengal	South 24 Parganas	Diamond Harbour DH & SSH	1144350918	13864198
West Bengal	Uttar Dinajpur	Raiganj DH & SSH	1173358593	14215636
Bihar	Darbhanga		370927056.9	4493907
Bihar	Patna		885437117.3	10727370
Madhya Pradesh	Niwari		708162108.1	8579623
Jharkhand	Dhanbad		456470546.4	5530295
Uttar Pradesh	Amethi		294067194.8	3562723
Uttar Pradesh	Hapur		293104317.2	3551058
Uttar Pradesh	Shamli		291499521.2	3531615
Rajasthan	Bikaner		988108215.6	11971265
Rajasthan	Jaipur		987987855.9	11969807
Rajasthan	Jhalawar		880069629.9	10662341
Rajasthan	Jodhpur		989552532	11988763
Rajasthan	Kota		878585193.6	10644357
Rajasthan	Udaipur		882878022.9	10696366
Chhattisgarh	Bastar		427083735.4	5174264
Chhattisgarh	Korea		427163975.2	5175236
Chhattisgarh	Rajnandgaon		426120857.8	5162598
Chhattisgarh	Surajpur		427003495.6	5173292
Chhattisgarh	Surguja		425519059.3	5155307
Assam	Biswanath		455891138.8	5523275
Assam	Charaideo		460464807.4	5578687
Assam	Dibrugarh		457776774.1	5546120
Assam	Hojai		464436677.5	5626807
Assam	Jorhat		460023488.5	5573340
Assam	Majuli		459461809.9	5566535
Assam	South Salmara Mancachar		454607302	5507721
Andhra Pradesh	YSR		1029108151	12467993
West Bengal	Bankura		1351378151	16372403
West Bengal	Kalimpong		1348329038	16335462
West Bengal	Kolkata		1298984731	15737639
West Bengal	Maldah		1354587743	16411288
West Bengal	Murshidabad		1352581748	16386985
West Bengal	Paschim Medinipur		1349973954	16355391
West Bengal	Purba Burdwan		1365826219	16547446
Tripura	Sepahijala		353888924.7	4287484
Tripura	West Tripura		353929044.6	4287970
Arunachal Pradesh	Anjaw		312644023.6	3787788
Arunachal Pradesh	Changlang		314409299.2	3809175

Arunachal Pradesh	East Kameng		312082345	3780983
Arunachal Pradesh	Kamle		311881745.5	3778553
Arunachal Pradesh	Kra Daddi		311199707.2	3770290
Arunachal Pradesh	Kurung Kumey		312202704.7	3782441
Arunachal Pradesh	Longding		316335054.4	3832506
Arunachal Pradesh	Lower Siang		313647021.1	3799940
Arunachal Pradesh	Lower Subansiri		311360186.8	3772234
Arunachal Pradesh	Namsai		313005102.7	3792163
Arunachal Pradesh	Pakke Kessang		311761385.8	3777095
Arunachal Pradesh	Shi Yomi		310918867.9	3766887
Arunachal Pradesh	Tirap		315933855.4	3827645
Arunachal Pradesh	Upper Siang		309394311.7	3748417
Arunachal Pradesh	Upper Subansiri		310838628.1	3765915
Arunachal Pradesh	West Kameng		311600906.2	3775150
Meghalaya	East Jaintia Hills		396965529.5	4809372
Meghalaya	North Garo Hills		383084044.1	4641193
Meghalaya	South Garo Hills		382201406.3	4630499
Meghalaya	South West Garo Hills		383926562	4651400
Meghalaya	South West Khasi Hills		384528360.5	4658691
Karnataka	Bengaluru Rural		1366589393	16556692
Karnataka	Vijaypura		926535021.7	11225285
Telangana	Adilabad		979357502.4	11865247
Telangana	Bhadradri Kothagudem		981203017.8	11887606
Telangana	Jagitial		978194025.3	11851151
Telangana	Jangoan		977592226.8	11843860
Telangana	Jayashankar Bhupalpally		979397622.3	11865733
Telangana	Jogulamba Gadwal		982526974.5	11903646
Telangana	Kamareddy		978354504.9	11853096
Telangana	Kumuram Bheem Asifabad		979317382.5	11864761
Telangana	Mahabubnagar		980561099.4	11879829
Telangana	Mahuababad		978474864.6	11854554
Telangana	Mancherial		978635344.2	11856498
Telangana	Medak		978474864.6	11854554
Telangana	Medchal Malkajgiri		976228150.2	11827334
Telangana	Nagarkurnool		981363497.4	11889550
Telangana	Nirmal		978916183.5	11859900
Telangana	Nizamabad		978194025.3	11851151
Telangana	Peddapalli		977712586.5	11845318
Telangana	Rajanna Sircilla		977953305.9	11848235
Telangana	Rangareddy		977632346.7	11844346
Telangana	Siddipet		977792826.3	11846291
Telangana	Suryapet		978474864.6	11854554
Telangana	Wanaparthy		981764696.4	11894411
Telangana	Warangal Rural		883070043.2	10698692
Telangana	Warangal Urban		882107165.6	10687026
Telangana	Yadadri Bhuvanagiri		977832946.2	11846777
Gujarat	Ahmedabad		1304882357	15809091

Gujarat	Arvalli		486410214.9	5893024
Gujarat	Bhavnagar		584832849.9	7085448
Gujarat	Gandhinagar		482237745.3	5842473
Gujarat	Gir Somnath		479991030.9	5815254
Gujarat	Jamnagar		581984337	7050937
Gujarat	Junagadh		478907793.6	5802130
Gujarat	Kachchh		484364100	5868235
Gujarat	Patan		486089255.7	5889136
Gujarat	Sabar Kantha		485848536.3	5886219
Gujarat	Surat		581783737.5	7048507
Gujarat	Valsad		479790431.4	5812823
Manipur	Imphal East		180657446.2	2188726
Manipur	Imphal West		180336487	2184837
Manipur	Jiribam		187237109.8	2268441
Manipur	Kakching		180898165.6	2191642
Manipur	Kamjong		181660443.7	2200878
Manipur	Kangpokpi		180938285.5	2192128
Manipur	Noney		184348477	2233444
Manipur	Pherzawl		184268237.2	2232472
Manipur	Tengnoupal		181700563.6	2201364
Jammu and Kashmir	Kishtwar		292609724.3	3545066
Jammu and Kashmir	PoJK		294455239.7	3567425
Jammu and Kashmir	Poonch		291325887.5	3529512
Jammu and Kashmir	Rajouri		290082170.6	3514444
Haryana	Charkhi Dadri		414927077	5026982
Haryana	Gurugram		588778083	7133245
Haryana	Karnal		588537363.6	7130329
Haryana	Yamunanagar		415007316.8	5027954
Uttarakhand	Dehradun		883591601.9	10705011
Maharashtra	Akola		692113819.3	8385193
Maharashtra	Chandrapur		694200054.1	8410468
Maharashtra	Dhule		692755737.7	8392970
Maharashtra	Gondia		694681492.9	8416301
Maharashtra	Kolhapur		969184448.1	11741997
Maharashtra	Latur		690509023.3	8365750
Maharashtra	Mumbai City		972207820.1	11778626
Maharashtra	Mumbai Suburban		972087460.4	11777168
Maharashtra	Nagpur		973076078.4	11789146
Maharashtra	Nanded		972955718.7	11787687
Maharashtra	Palghar		691512020.8	8377902
Maharashtra	Sangli		969545527.2	11746372
Maharashtra	Solapur		970428165	11757065
Maharashtra	Yavatmal		693076696.9	8396858
Nagaland	Kiphire		331915716.1	4021271
Nagaland	Kohima		332156435.5	4024187
Nagaland	Longleng		334523509.6	4052865
Nagaland	Mon		335245667.8	4061615

Nagaland	Peren		335045068.3	4059184
Nagaland	Phek		331394157.4	4014952
Nagaland	Tuensang		332838473.8	4032451
Nagaland	Zunheboto		332196555.4	4024674
Himachal Pradesh	Kinnaur		595714908.3	7217287
Delhi	New Delhi		692934843	8395140
Delhi	South East		693255802.2	8399028
